# Ultrasound-enhanced co-fermentation of Yunnan Arabica coffee with rose petals: Mass transfer, microbial succession, and molecular modeling

**DOI:** 10.1016/j.ultsonch.2026.108127

**Published:** 2026-10-24

**Authors:** Lihui Yu, Shengjie Duan, Ruijuan Yang, Zezhu Du, Jinya Dong, Yunfei Ge, Li Feng, Rongxian Yu, Yanan Li, Lili Gao, Guangyan Yang, Ruixue Li, Cunchao Zhao, Xiaocui Du, Chongye Fang

**Affiliations:** a College of Food Science and Technology, Yunnan Agricultural University, Kunming 650201, China; b Key Laboratory of Development and Utilization of Food and Medicinal Resources, Ministry of Education, Yunnan Agricultural University, Kunming 650201, China; c College of Agronomy and Biotechnology, Yunnan Agricultural University, Kunming 650201, China; d Jiangnan University School of Food Science and Technology, Wuxi 214122,China; e School of Food Science and Engineering, South China University of Technology, Guangzhou, Guangdong 510640, China; f Yunnan Plateau Characteristic Agricultural Industry Research Institute, Kunming 650201, China; g Faculty of Engineering and Agro-Industry, Maejo University, Chiang mai 50290, Thailand

**Keywords:** Acoustic-cavitation-mediated multiphase mass transfer, Microbial succession, Nonequilibrium molecular dynamics, Density functional theory, Coffee aroma profile

## Abstract

Plant-based co-fermentation can diversify coffee flavor profiles, yet multiphase systems are often limited by interfacial mass-transfer resistance and stochastic microbial succession. Here, we investigated the effects of non-thermal pulsed ultrasound (20 kHz, 0.5 W mL^−1^, 50 % duty cycle, 30 ± 0.5 °C) on the co-fermentation of Yunnan Arabica coffee with *Rosa* ’Crimson Glory’ petals. Acoustic-assisted processing enhanced the apparent solid–liquid mass-transfer rate constant (*k*_obs_ = 0.082 min^−1^) and accelerated acidification kinetics. Rheological and acoustic-attenuation monitoring identified 60 % (w/w) rose loading as the operational upper bound, beyond which viscous damping impaired energy transmission. High-throughput 16S rRNA and ITS sequencing combined with untargeted LC–MS/MS metabolomics showed that rapid acidification and acoustic-assisted co-fermentation established a selective habitat filter, promoting a lactic acid bacteria guild (*Lactiplantibacillus, Lactococcus*, and *Weissella*; 72.8–80.1 % bacterial abundance) alongside inoculated *Saccharomyces*. This microbial restructuring redirected carbon and nitrogen fluxes, expanding the total free amino acid pool to 125.4μmol g^−1^ DW in RF60 and enriching floral–fruity volatiles such as linalool, *β*-myrcene, and eugenol. Complementary nonequilibrium molecular dynamics and density functional theory calculations provided simulation-supported mechanistic plausibility regarding shear-induced loop reorganization in model enzymes (Atf1 and Pep4) and barrier reductions (ΔΔ*G*^‡^ = −107.9 and − 49.9 kJ mol^−1^) in polarized model reactions. Finally, computational fluid dynamics modeling of a simulated 500 L external-loop multiprobe reactor predicted 90.2 % mass-transfer fidelity, with techno-economic analysis estimating a capital payback period of 0.88–1.18 years.

## Graphical abstract

**Figure:**
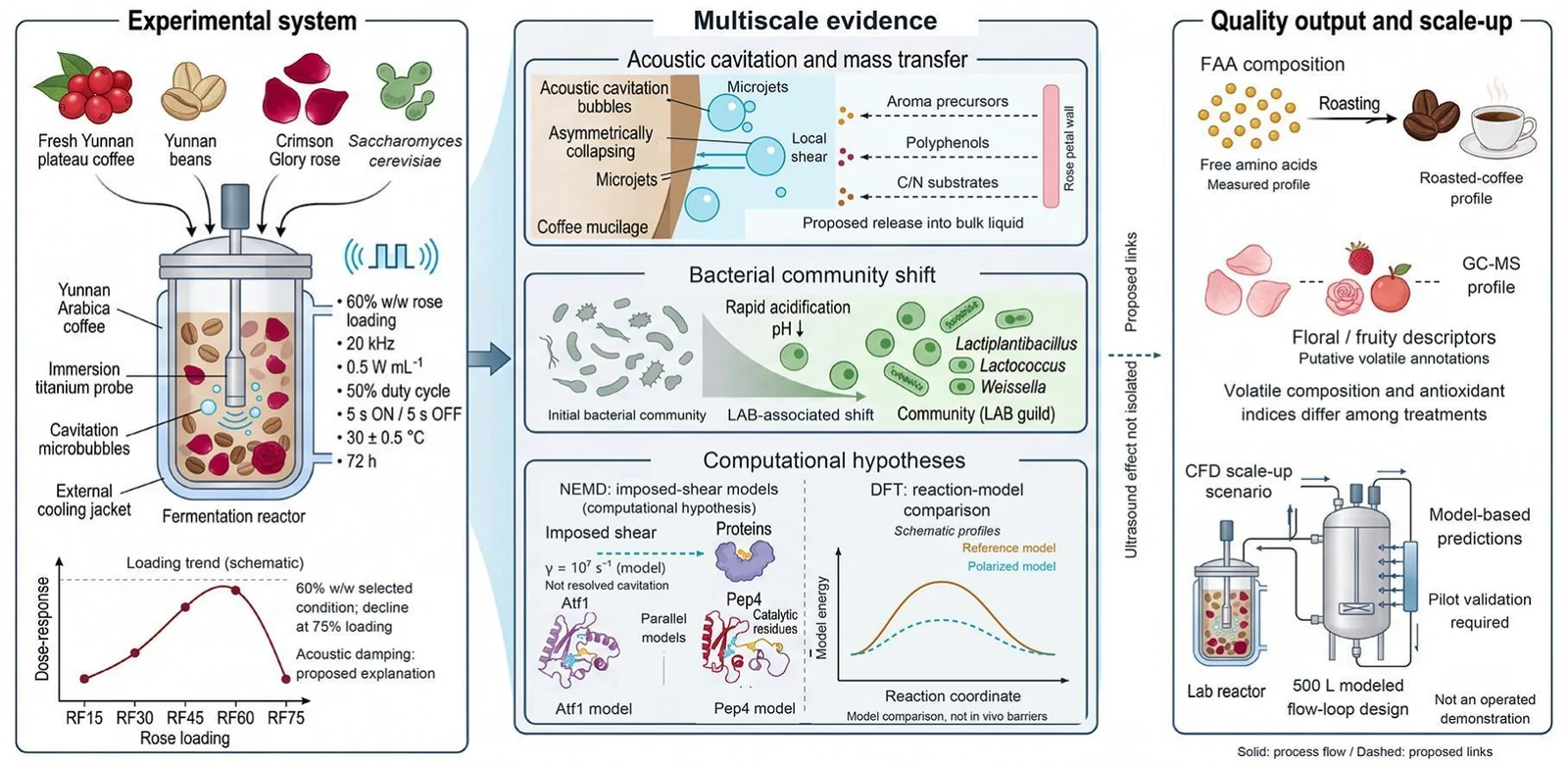

## 1 Introduction

Fermentation is a central metabolic-engineering stage in which the chemical profile of the food matrix is reshaped and distinctive sensory attributes are generated [1]. As one of the most widely consumed specialty beverages worldwide [2], coffee derives its flavor quality not only from cultivar and regional microclimate but also, to a considerable extent, from the biochemical transformations occurring during postharvest fermentation. During this stage, endogenous and exogenous microorganisms mediate complex cascade reactions that degrade proteins, polysaccharides, and lipids in the coffee pulp and beans into key flavor precursors, including low-molecular-weight reducing sugars, free amino acids, and organic acids. These precursors subsequently contribute to the sensory foundation of roasted coffee through Maillard reactions, Strecker degradation, and thermal esterification [3], [4], [5]. In recent years, Arabica coffee (*Coffea arabica*) from the Pu’er (Simao) production region of Yunnan has attracted considerable attention in the international specialty-coffee market because of its pronounced acidity and sweetness [6]. However, conventional single-substrate fermentation often has difficulty meeting the market’s increasing demand for coffee with rich, multidimensional floral and fruity profiles. Plant-based co-fermentation [7] has therefore emerged as a promising strategy for broadening the flavor dimensions of coffee and increasing product value. One candidate plant resource is Crimson Glory rose (*Rosa ‘*Crimson Glory*’*) [8], which contains characteristic aroma precursors, including geraniol, citronellol, linalool, and 2-phenylethanol, as well as bioactive polyphenols. However, this strategy faces substantial physical and biochemical constraints in conventional solid–liquid multiphase co-fermentation systems. The dense cuticular and pectin-rich layers associated with coffee beans, together with the highly lignified cell walls of rose petals, create considerable interfacial mass-transfer resistance. As a result, the transport of intracellular aroma precursors into microbial cells and their diffusion across phases remain highly limited [9]. In parallel, microbial communities undergoing spontaneous succession exhibit substantial variability and stochasticity, which can lead to non-targeted metabolic competition, prolonged fermentation cycles, and poor reproducibility of flavor development between batches [10]. Accordingly, within the interdisciplinary fields of food bioprocess engineering and synthetic biology, the development of a nonthermal, sustainable, and precisely controllable physical-field intervention is an important scientific and engineering challenge. Such a technology should overcome interfacial mass-transfer barriers and enable the controlled progression of complex co-fermentation networks toward precision-driven fermentation [11].

As an energy-field-mediated technology with considerable industrial potential, power ultrasound offers a promising approach to overcoming the mass-transfer and metabolic constraints of the multiphase systems described above [12]. When high-intensity acoustic waves propagate through a fluid medium, acoustic cavitation can induce the nucleation, growth, and asymmetric collapse of cavitation bubbles on microsecond timescales. These events generate intense hydrodynamic shear, acoustic streaming, transient shock waves, and localized microenvironments of extremely high temperature and pressure, reaching approximately 5000 K and 1000 atm under certain conditions [13]. This distinctive physicochemical coupling can generate substantial mechanical forces that disrupt plant cell-wall architectures and microbial membrane structures across multiple scales, reduce boundary-layer mass-transfer resistance, and facilitate the efficient transport of substrates and metabolites across phase boundaries [14]. Within an appropriate acoustic energy–density range, it may also induce acoustic stress adaptation in microorganisms, thereby altering cell-membrane permeability and activating otherwise latent secondary metabolic pathways [15]. Recent pioneer studies have demonstrated that power ultrasound can accelerate coffee fermentation kinetics and modulate volatile aroma compounds [16]. Nevertheless, existing literature remains largely confined to macroscopic, empirical characterizations. In complex multiphase plant-based co-fermentation ecosystems, fundamental questions regarding how non-thermal acoustic energy fields relieve interfacial boundary-layer resistance and drive the directed succession of core microbial functional guilds remain insufficiently understood. Moreover, how cavitation shear and localized polar microenvironments interface with key metabolic enzymes across atomic and quantum scales has yet to be elucidated.

A more fundamental scientific limitation is that the mechanistic understanding of acoustic-field regulation of biological fermentation remains largely confined to macroscopic physical mass transfer and has yet to reach the atomic and molecular scales. Fermentation is fundamentally governed by a series of highly specific biochemical cascade reactions catalyzed by enzymes such as esterases, proteases, and alcohol acyltransferases [17]. From the intersection of sonochemistry and mechanobiocatalysis, the effects of an acoustic field on a fermentation system are unlikely to be limited to improvements in macroscopic mass transfer across phase interfaces [18]. Nonequilibrium hydrodynamic shear stresses and energy pulses generated within cavitation microenvironments may directly perturb the three-dimensional conformations of free enzymes or key intracellular metabolic enzymes. Such perturbations could disrupt weak noncovalent interactions and thereby alter the accessibility of catalytic active sites and flexible loops [19].

At the same time, cavitation-generated radicals, such as hydroxyl radicals (· OH), together with localized polar microenvironments, may substantially influence the thermodynamic landscape of biochemical reactions [20]. However, how acoustic stress regulates the conformational flexibility of metabolic enzymes at the molecular scale remains unclear. It is also unknown how this physical intervention changes, from thermodynamic and kinetic perspectives, the activation Gibbs free-energy barriers (Δ*G*‡) of key reactions, including aroma esterification and protein degradation or amino acid release. These unresolved questions constitute a theoretical “black box” in the fields of food sonochemistry and biocatalysis. Integrating molecular dynamics simulations (*molecular dynamics, MD*) with quantum-chemical calculations, particularly density functional theory (*quantum chemistry, QC/density functional theory, DFT*), provides a necessary route for deciphering the underlying physicochemical logic of these sonochemical mechanisms.

Building on the theoretical gaps and industrial demands outlined above, this study used a co-fermentation system comprising Yunnan Arabica coffee and Crimson Glory rose as a model system and developed a custom continuous-phase sonobioreactor. We formulated the following central hypothesis: the synergistic coupling of acoustic cavitation and plant-substrate addition accelerates multiphase mass-transfer fluxes at the macroscopic level, while non-lethal acoustic shear theoretically provides mechanical stimuli that modulate the conformational dynamics of model metabolic enzymes at the molecular level [21]. These combined physical–chemical influences work in concert to restructure microbial ecological niches and reshape the resulting flavor-precursor landscape. To test this hypothesis systematically from the bottom up, we established a cross-scale analytical framework spanning macroscopic phenotypes, microscopic mechanisms, and engineering applications. First, 16S rRNA and ITS amplicon sequencing, targeted chromatographic analyses of free amino acids, GC–MS/O analysis of floral and fruity volatile odorants, and untargeted LC–MS/MS metabolomics were integrated to map the interaction topology between microbial communities and metabolic networks under acoustic-field treatment. Second, nonequilibrium molecular dynamics simulations (*nonequilibrium molecular dynamics, NEMD*) were combined with density functional theory calculations (*density functional theory, DFT*) to investigate, at the atomic and electronic scales, the mechanochemical coupling mechanism by which shear forces generated by acoustic cavitation alter the accessibility of enzyme catalytic centers and modulate the activation-energy barriers of biochemical reactions. Finally, the engineering and economic feasibility of industrial scale-up was systematically evaluated on the basis of acoustic energy yield, reactor flow-field design, and comparative gains in product value. This study provides an engineering strategy for the directed development of flavor profiles in high-quality specialty coffee and establishes a cross-scale mechanistic framework for understanding the targeted regulation of complex biological fermentation systems by ultrasonic energy fields.

## 2 Materials and methods

### 2.1 Raw materials and sonobioreactor setup

Fresh coffee cherries were collected from *Coffea arabica* L. cv. Catimor CIFC7963 cultivated by *Yunnan Simao Beigui Coffee Co., Ltd.* (Simao District, Pu’er City, Yunnan Province, China; 100.97° E, 22.78° N; elevation, 1200 m). Cherries free of disease, insect damage, and mechanical injury, and with uniform maturity, were selected, with a soluble-solids content of 18.3 ± 0.5°Brix. Within 24 h of harvest, the cherries were rinsed with sterile water and mechanically depulped to obtain fresh coffee beans retaining the pectin-rich mucilage layer.

Petals of *Rosa ‘*Crimson Glory*’* were collected at full bloom from healthy flowers cultivated at an organic farm in Kunming, Yunnan Province. The petals were transported to the laboratory at 4 °C under light-protected conditions, gently rinsed with sterile physiological saline, and subjected to mild surface decontamination using short-wave ultraviolet irradiation at 254 nm for 20 min to reduce interference from exogenous microorganisms.

The sonobioreactor consisted of a jacketed stainless-steel fermentation vessel integrated with a programmable ultrasonic-field control unit. The system was equipped with a 25 mm diameter titanium-alloy horn probe operating at 20 ± 0.5 kHz with a rated power of 500 W. The actual acoustic power density delivered to the liquid phase (0.3 to 0.8 W mL^−1^) was calibrated calorimetrically (P_cal = M·Cp·(dT/dt)_t=0_) prior to fermentation using a calibrated multi-junction thermocouple array (five measurement points across radial and axial positions) during rapid mechanical pre-recirculation. To validate the CFD acoustic field model, the calorimetrically measured net power was benchmarked against the volume-integrated acoustic dissipation rate computed from the Helmholtz acoustic wave equation in CFD; the empirical calorimetric values matched the numerical acoustic dissipation predictions within a relative deviation of 5.8 % to 7.2 % across the operating range (Supplementary Table S1).

#### 2.1.1 Experimental design and reference boundaries

The fermentation systems were prepared with different levels of rose petals relative to the mass of fresh coffee beans. Seven treatment groups were established, with three independent fermentation batches per group, corresponding to three independent fermentation vessels and three biological replicates (*n* = 3): **NF** (natural-fermentation control, code **Con**): no yeast inoculation and no rose addition (0 % rose). **YF** (yeast-fermentation control, code **Con-G**): inoculation with commercial brewing yeast, *Saccharomyces cerevisiae*, at 1.0 × 10^7^ CFU g^−1^ based on the mass of fresh coffee beans, without rose addition (0 % rose). **RF15**, **RF30**, **RF45**, **RF60**, and **RF75** (co-fermentation treatments, codes **FRC-G15** to **FRC-G75**): inoculation with the same amount of yeast as in the YF treatment, combined with the addition of *Rosa ‘*Crimson Glory*’* petals at 15 %, 30 %, 45 %, 60 %, and 75 % (w/w), respectively.

The experimental design was intended to compare natural fermentation, yeast-only fermentation, and yeast-assisted co-fermentation across a gradient of rose addition levels. Because the experimental matrix did not incorporate a parallel non-sonicated co-fermentation control at each rose level, the individual main effect of rose loading and the interaction between acoustic treatment and rose addition cannot be statistically decoupled. Consequently, phenotypic differences across the RF series reflect the combined sonochemical-assisted co-fermentation process rather than an isolated physical-field effect alone. All comparative inferences are strictly restricted to this defined operational boundary. Fermentation was conducted for 72 h. Samples collected at defined stages during fermentation were designated process samples and coded **R**, comprising 81 measured samples in total. At the end of fermentation, the fermented material was hot-air-dried at 40 °C to a moisture content of 10–12 % and then subjected to medium roasting under a standardized thermal profile at 200 °C for 12 min. The roasted material was ground, sieved to a particle size of 500 ± 10 μm, and stored in sealed containers under light-protected conditions. These samples were designated endpoint samples and coded **C**, comprising 21 formal samples in total.

### 2.2 Determination of fermentation kinetics, basic physicochemical properties, and antioxidant activity

Solid–liquid interfacial mass-transfer kinetics of total phenolics were determined within Crank’s film-theory framework: dC_t_/dt = *k*_mt_·a·(C_∞_ − C_t_), which integrates to ln[C_∞_ / (C_∞_ − C_t_)] = *k*_oβs_·t, where *k*_oβs_ = *k*_mt_·a represents the apparent volumetric mass-transfer rate constant (min^−1^), a is the specific solid–liquid interfacial area (m^2^ m^−3^), and C_t_ and C_∞_ are the liquid-phase concentrations at time t and equilibrium, respectively. Kinetic parameters were estimated via non-linear least-squares regression (*R*^2^ ≥ 0.985) of time-course release data (0–60 min) under the assumption of well-mixed bulk liquid and invariant structural interfacial area during initial extraction. Fermentation dynamics were monitored by collecting samples at key time points during fermentation (0, 24, 48, and 72 h). The pH was measured using a pH meter calibrated with standard buffer solutions (Sartorius PB-10). Total titratable acidity (TA) was determined by potentiometric titration with 0.1 mol L^−1^ NaOH to pH 8.2 and expressed as citric acid equivalents (g L^−1^). Total soluble solids (TDS) were measured using a digital refractometer (PAL-1, Atago, Japan) [22]. Fermented materials were homogenized at low temperature in phosphate buffer (0.05 mol L^−1^, pH 7.0) and centrifuged at 10,000 × *g* and 4 °C for 15 min to obtain crude enzyme extracts. Extracellular esterase activity was determined using a *p*-nitrophenyl acetate (pNPA) colorimetric assay, with one unit (U) defined as the amount of enzyme that released 1 μmol of *p*-nitrophenol per minute. Acid protease activity was determined using the Folin–Casein method, with one unit defined as the amount of enzyme that hydrolyzed substrate to release 1 μg of tyrosine equivalents per minute [23]. Fourteen basic physicochemical and antioxidant parameters were measured in the 21 endpoint coffee samples. *In vitro* antioxidant activity was systematically evaluated using ultrasound-assisted extraction at 40 kHz and 400 W for 10 min. DPPH radical-scavenging activity was measured at 517 nm. Ferric-reducing antioxidant power (FRAP) was determined using a TPTZ working solution at 593 nm. Oxygen radical absorbance capacity (ORAC) was assessed using sodium fluorescein as the fluorescent probe (70 nmol L^−1^) and AAPH as the radical initiator, with fluorescence kinetics monitored for 90 min at excitation and emission wavelengths of 485 and 528 nm, respectively. All antioxidant indices were expressed as micromoles of Trolox equivalents per gram of dry weight (μmol TE g^−1^ DW) [24].

### 2.3 Targeted profiling of amino acids (HPLC) and aroma volatiles (GC–MS/O)

For targeted absolute quantification of amino acids [25], 21 endpoint coffee-powder samples were accurately weighed, with sample masses ranging from 19.7 to 20.9 mg. The exact dry mass of each sample was recorded as *m*_sample_. Each sample was extracted ultrasonically in 80 % chromatographic-grade methanol in an ice bath for 30 min. After centrifugation at 12,000 rpm for 10 min, the supernatant was collected and subjected to fully automated online pre-column derivatization with o-phthalaldehyde (OPA) and 9-fluorenylmethyl chloroformate (FMOC-Cl). Chromatographic separation was performed using an Agilent 1290 UHPLC system equipped with a ZORBAX Eclipse AAA column (4.6 mm × 150 mm, 5 μm). The mobile phases consisted of 20 mmol L^−1^ ammonium acetate in water (pH 6.8) and acetonitrile, applied using a gradient elution program at a flow rate of 1.0 mL min^−1^. Detection was conducted at 338 and 262 nm. Calibration curves were established using 25 authentic free-amino-acid standards in combination with isotopically labeled internal standards (^13^C/^15^N-labeled). The measured concentrations were normalized to the accurately weighed dry sample masses and expressed as both mass concentrations (μg mg^−1^ DW) and molar concentrations (μmol g^−1^ DW) (Supplementary Table S2A1–S2A3).

For GC–MS/O analysis of volatile flavor compounds [26], 2.00 g of each endpoint coffee powder was placed in a 20 mL headspace vial, followed by the addition of 10 μL of 2-octanol as the internal standard (1.00 mg L^−1^). The vials were equilibrated at 50 °C for 10 min using a CTC CombiPAL autosampler. Headspace extraction was then performed for 30 min using a 50/30 μm DVB/CAR/PDMS fiber. Analyses were conducted on a Trace 1610–TSQ 9610 GC–MS system equipped with a Sniffer 9100 olfactory detection port and a polar DB-WAX capillary column (60 m × 0.25 mm × 0.25 μm). The carrier-gas flow rate was maintained at 1.0 mL min^−1^. The oven temperature program was as follows: 50 °C for 3 min; increase to 100 °C at 5 °C min^−1^; increase to 150 °C at 3 °C min^−1^; and finally increase to 240 °C at 10 °C min^−1^, followed by a 10 min hold. The column effluent was split equally between the mass spectrometer and the olfactory detection port. The mass spectrometer was operated in electron-ionization mode at 70 eV over a scan range of *m*/*z* 35–450. Compound identities were assigned by integrating retention indices determined using C8–C40 *n*-alkanes, matching mass spectra against the NIST 2020 library, and comparing sensory descriptors provided by four rigorously trained assessors. Relative abundances were determined retrospectively from the integrated peak areas in the original CDF files. Odor activity values (OAVs) were then calculated for the identified volatile compounds.

### 2.4 Global untargeted metabolomics analysis (LC-MS/MS)

Untargeted LC–MS/MS metabolomics was performed on 21 formal endpoint samples and three pooled quality-control samples (QC01–QC03). The QC samples were prepared by mixing equal volumes of all samples and were used to monitor instrumental reproducibility and correct signal drift. Samples were extracted with methanol–water (80:20, *v/v*) and filtered through 0.22 μm PTFE membrane filters. Analyses were conducted using a Vanquish UHPLC system coupled to an Orbitrap Exploris 120 mass spectrometer equipped with an electrospray ionization (ESI) source. Chromatographic separation was performed on an ACQUITY UPLC BEH C18 column (2.1 mm × 100 mm, 1.7 μm) using 0.1 % formic acid in water as mobile phase A and acetonitrile containing 0.1 % formic acid as mobile phase B, at a flow rate of 0.3 mL min^−1^ [27]. At the beginning of the injection sequence, five consecutive QC injections were used to equilibrate the system. Subsequently, one QC injection was inserted after every seven formal samples. The mass spectrometer was operated in data-dependent acquisition (DDA) mode with alternating positive- and negative-ion acquisition. The full-scan resolution was set to 60,000 FWHM, the MS/MS resolution to 15,000 FWHM, and the stepped collision energies to 20/30/40 eV. A total of 48 raw mzXML files acquired in positive- and negative-ion modes were imported into Compound Discoverer 3.1 and XCMS for peak alignment and QC-signal normalization using a LOESS-based algorithm. Unstable features with a coefficient of variation (CV) greater than 30 % in the QC samples were excluded. Metabolite annotation was based on high-resolution accurate mass measurements with a mass deviation of less than 5 ppm and MS/MS fragment matching against the BiotreeDB, HMDB, and MassBank databases. The resulting annotations met Metabolomics Standards Initiative (MSI) Level 2 identification criteria.

### 2.5 Microbial community dynamics via 16S rRNA and ITS amplicon sequencing

For the 81 measured process samples collected during fermentation, including biological replicates from the seven treatment groups at 0, 24, 48, and 72 h, total genomic DNA was extracted using the FastDNA™ Spin Kit for Soil [28]. The YF group lacked three biological replicates at 0 h; consequently, the complete 0–72 h longitudinal dataset comprised 72 samples, whereas the common dataset covering all treatments from 24 to 72 h comprised 63 samples. The V3–V4 region of the bacterial 16S rRNA gene and the ITS1–ITS2 region of the fungal rRNA gene were amplified in parallel. Bioinformatic analysis revealed that the inoculated *Saccharomyces cerevisiae* overwhelmingly dominated the fungal community across all fermentation batches (>98.7 % relative abundance; detailed in Supplementary Fig. S7 and Table S3), showing negligible ecological succession. Therefore, downstream community succession and network modeling focused strictly on bacterial guild restructuring (16S rRNA), while yeast viability was tracked to monitor primary metabolic activity. Denoising with DADA2 was used to generate an amplicon sequence variant (ASV) feature table, and taxonomic annotation was performed against the SILVA 138 and UNITE 8.0 databases. Alpha diversity was evaluated using the Chao1 richness estimator and Shannon index. Beta diversity was assessed based on Bray–Curtis dissimilarities using principal coordinates analysis (PCoA) and nonmetric multidimensional scaling (NMDS). Differentially representative microbial groups were identified using LEfSe with an LDA score > 3.5 and *p* < 0.05. Potential metabolic pathways were predicted using PICRUSt2 and FUNGuild.

### 2.6 Molecular dynamics (MD) simulations under acoustic stress models

To investigate the mechanism by which cavitation-induced shear forces affect key flavor-forming enzymes, yeast alcohol acyltransferases ATF1/2 and endogenous proteases were selected as model enzymes [29]. Protein structures were obtained from the Protein Data Bank (PDB) or predicted using AlphaFold2. All-atom simulations were performed using GROMACS 2022.4 with the OPLS-AA force field [30]. Each protein model was placed in a periodic cubic TIP3P water box with a side length of 10 nm. Na^+^ and Cl^−^ ions were added to maintain a physiological ionic strength of 0.15 mol L^−1^. Following energy minimization and sequential NVT/NPT equilibration, nonequilibrium molecular dynamics (NEMD) simulations were conducted during the production phase. A fluid shear-strain tensor was applied at a shear rate of *γ* = 10^7^ s^−1^ to approximate the transient local strain rates experienced within the nanometer boundary layer of collapsing cavitation microjets (*v* ≈ 100 m s^−1^, *δ* ≈ 10 nm). This microscopic boundary condition differs fundamentally from the bulk-averaged continuum shear fields calculated by reactor-scale CFD (>10^4^ s^−1^). While homogeneous planar shear in NEMD represents an idealized rheological surrogate rather than an exact replica of the chaotic, shock-wave-induced multiphase cavitation field, it allows atomistic evaluation of mechanical stress dissipation across enzyme loops within a feasible 200 ns trajectory. The effects of acoustic stress on enzyme conformational flexibility and active-pocket accessibility were quantitatively characterized by analyzing the root-mean-square deviation (RMSD), root-mean-square fluctuation (RMSF), solvent-accessible surface area (SASA), and free-energy landscapes (FELs) of the catalytic centers mapped by principal component analysis (PCA).

### 2.7 Quantum chemical (QC) calculations of sonochemical reaction barriers

Density functional theory (*DFT*) was used to evaluate the activation barriers of esterification reactions, including the esterification of geraniol or ethanol with acetic acid, and peptide hydrolysis under sonochemical microenvironmental conditions. All calculations were performed using Gaussian 16 [31]. Ground-state molecular geometries and transition states (*TSs*) were optimized using the M06-2X hybrid functional with the 6-31G(d) basis set. The corresponding transition states were verified by intrinsic reaction coordinate calculations (*IRCs*) to confirm their proper connection to the respective reactants and products [32]. Single-point energy calculations were subsequently performed on the optimized structures using the higher-level 6–311 + G(d,p) basis set. Zero-point vibrational energy (ZPE) and thermal corrections were incorporated to obtain standard Gibbs free energies at 298.15 K. Activation free-energy barriers are rigorously and consistently designated as Δ*G*^‡^ throughout the text, and the barrier attenuation induced by the polarized microreaction model relative to bulk water is defined as ΔΔ*G*^‡^ [33]. In combination with the SMD implicit-solvent model (*SMD*), the calculations compared the microscopic catalytic effects on the reaction potential-energy surfaces of bulk aqueous media and cavitation microreaction zones enriched with short-chain organic-acid buffer salts [34].

### 2.8 Statistical analysis and multi-omics data integration

All endpoint physicochemical, volatile-compound, and amino-acid data (*n* = 3) were reported as the mean ± standard deviation (SD). Differences among multiple groups for individual variables were assessed using one-way analysis of variance (*one-way ANOVA*) in SPSS 29.0, followed by Tukey’s honestly significant difference (*Tukey’s HSD*) post hoc multiple-comparison test. For multiple-hypothesis testing, all *p* values were adjusted using the Benjamini–Hochberg procedure to control the false discovery rate (FDR) at *q* < 0.05. Longitudinal statistical analyses of the microbial time-series data (R samples) were performed using a repeated-measures framework, with fermentation vessel included as a random effect, implemented through repeated-measures PERMANOVA or a linear mixed-effects model.

Multi-omics data integration and association analyses were conducted in R (v4.3.2) and Python (v3.10) environments: Metabolomics data were subjected to Pareto scaling, followed by unsupervised principal component analysis (PCA) and supervised orthogonal partial least-squares discriminant analysis (OPLS-DA) using SIMCA 17.0. Key differential metabolites between groups were screened using the criteria of variable importance in projection (VIP) > 1.0, |log_2_ fold change| > 1.0, and *q* < 0.05. Mantel tests and Procrustes analyses were used to evaluate the global topological concordance between microbial community succession based on 16S rRNA and ITS data and endpoint flavor metabolites, using the matched dataset covering 24–72 h. Microbe–metabolite interaction networks were constructed from significant Spearman correlations (|*r*| > 0.7, *q* < 0.01), visualized in Cytoscape (v3.9.1), and analyzed in terms of network density and node centrality. Cross-scale integration was performed using multi-block partial least-squares analysis (*multi-block PLS*) in the mixOmics package. An integrated system-level regulatory model was constructed to link acoustic-energy input, the rose-addition gradient, succession of core microbial taxa, enzyme conformational dynamics and reaction-energy barriers, and endpoint floral–fruity aroma phenotypes.

## 3 Results and discussion

### 3.1 Acoustic field characterization and its modulation of mass transfer, fermentation kinetics, and antioxidant capacity

In conventional plant-based co-fermentation systems, highly lignified cell walls and dense pectin layers impose substantial barriers to interfacial mass transfer [35]. To overcome this physical limitation, we developed a pulsed sonobioreactor specifically for this study (Fig. 1a). Forced external recirculation cooling maintained the system at 30 ± 0.5 °C, thereby decoupling thermal effects from acoustic effects at the experimental-design level. This configuration ensured that subsequent phenotypic differences could be attributed to the nonthermal physical field and sonochemical stresses. Calorimetric calibration demonstrated linear power delivery accuracy across programmed outputs (*R*^2^ = 0.993, Fig. 1c; validated in Supplementary Table S1), while CFD acoustic pressure mapping captured the inherent spatial energy gradient extending radially from the radiating horn face (Fig. 1b;Supplementary Fig. S1a,b). Numerical discretization was validated through grid independence analysis, confirming energy convergence at 8.5 × 10^5^ polyhedral elements with a Grid Convergence Index (GCI) below 0.5 % (Supplementary Fig. S1c). The apparent interfacial mass-transfer rate constant (*k*_oβs_) did not increase monotonically with acoustic power density but reached a kinetic maximum at 0.5 W mL^−1^ (0.082 min^−1^, Fig. 1d). This non-monotonic profile is governed by acoustic cavitation shielding: above 0.5 W mL^−1^, an excessively dense cloud of cavitation bubbles coalesces directly at the radiating horn tip, forming an acoustic cushion that scatters and reflects acoustic waves back toward the transducer. This shielding effect impedes acoustic wave propagation into the bulk medium, causing mechanical dissipation via bubble coalescence and resulting in the observed mass-transfer attenuation [36], [37], [38].

**Fig. 1:**
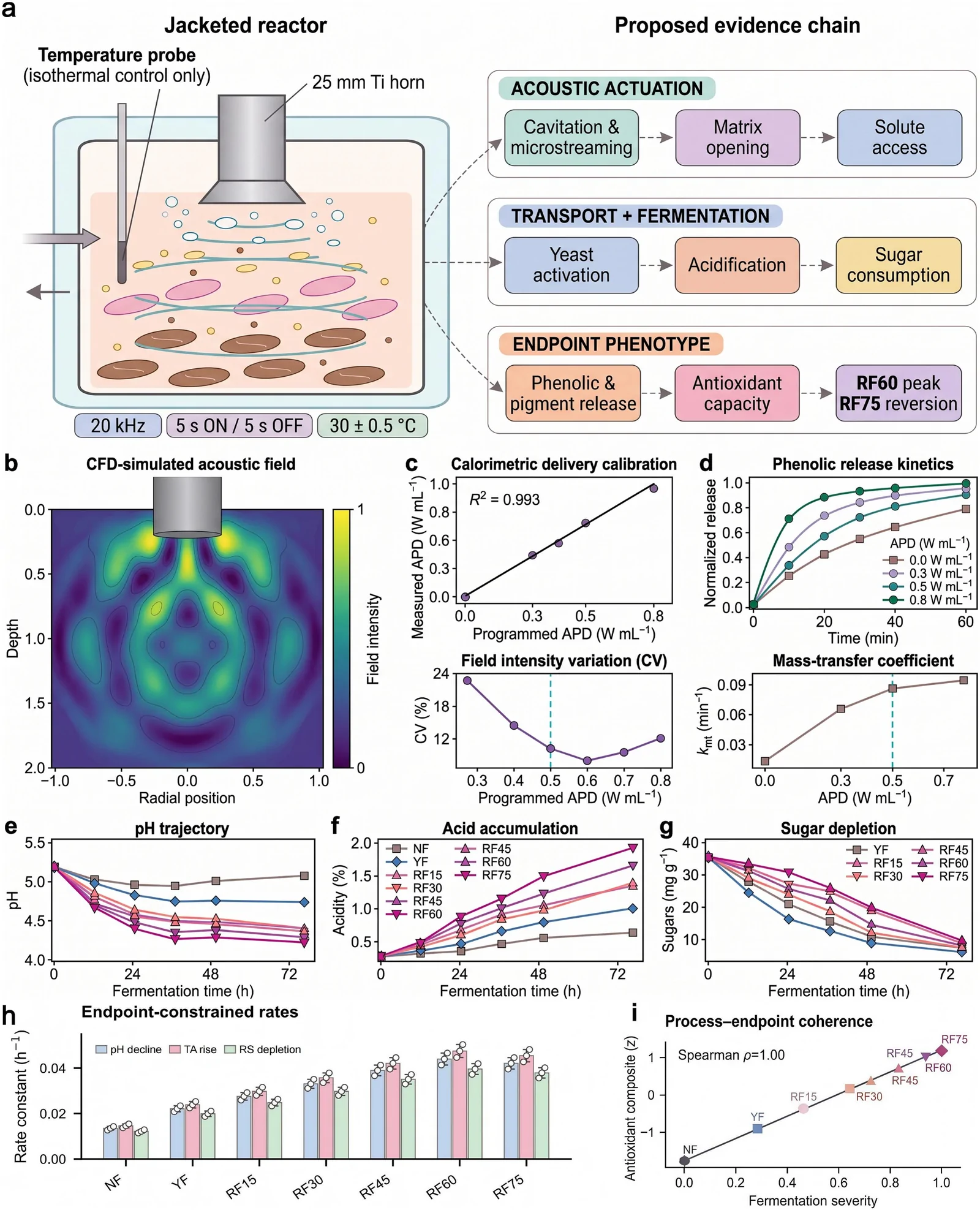
**Acoustic field characterization, mass-transfer validation, and fermentation kinetics in the multiphase sonobioreactor. (a)** Schematic of the jacketed sonobioreactor and the proposed mechanistic evidence chain. **(b)** Simulated spatial distribution of the acoustic field. **(c)** Calorimetric validation of the programmed versus delivered acoustic power density (APD). **(d)** Phenolic release kinetics and corresponding mass-transfer coefficients (*k*_mt_), indicating an optimal threshold at 0.5 W mL^−1^. **(e–g)** Trajectories of pH decline, acid accumulation, and sugar depletion over 72 h across different fermentation groups. **(h)** Endpoint-constrained kinetic rate constants. **(i)** Spearman correlation demonstrating high process–endpoint coherence between fermentation severity and integrated antioxidant output.

This physical restructuring of mass transfer acted as an accelerator of the fermentation network [39]. The macroscopic kinetic trajectories of fermentation (Fig. 1e–g) showed that acoustic-field treatment redirected microbial metabolic fluxes. Compared with the prolonged and relatively slow lag phase observed in natural fermentation (NF), the rose-containing treatments subjected to acoustic-field stimulation (*the RF series*) exhibited higher rates of carbon metabolism. Over the 0–72 h fermentation period, the RF treatments showed a rapid decline in pH, accompanied by pronounced accumulation of total acidity (TA) and marked depletion of reducing sugars (RS). Analysis of the endpoint-constrained rate constants (Fig. 1h) indicated that the RF60 treatment reached the highest overall flux across the principal kinetic parameters. The strong covariation between process kinetics and endpoint phenotypes (Spearman’s *ρ* = 1.00) (Fig. 1i) supports the interpretation that moderate nonequilibrium mechanical shear did not induce extensive lethal cell disruption. Instead, it may have functioned as a positive acoustic-stress signal associated with acoustic stress adaptation [40], enhancing transmembrane substrate uptake and glycolytic flux in yeast and thereby shortening the fermentation cycle [41].

To systematically characterize the endpoint quality output of the sonochemical-coupled fermentation system, we established an endpoint phenotypic profile comprising 14 physicochemical and antioxidant variables (Fig. 2a). Multivariate principal component analysis (PCA) (Fig. 2b) showed a directional shift in the overall phenotype along PC1, which explained 98.9 % of the total variance. Across the dosage range from NF to RF60, the fermentation matrix shifted from a predominantly coffee-based substrate toward a composite matrix enriched in secondary metabolites, including total phenolic content (TPC), total flavonoid content (TFC), and total anthocyanin content (TAC). In parallel, antioxidant activities associated with electron- or hydrogen-donating capacity, including DPPH radical-scavenging activity, ABTS radical-scavenging activity, and ferric-reducing antioxidant power (FRAP), increased progressively (Fig. 2c–h) and reached their highest overall level in the RF60 treatment (Fig. 2i).

**Fig. 2:**
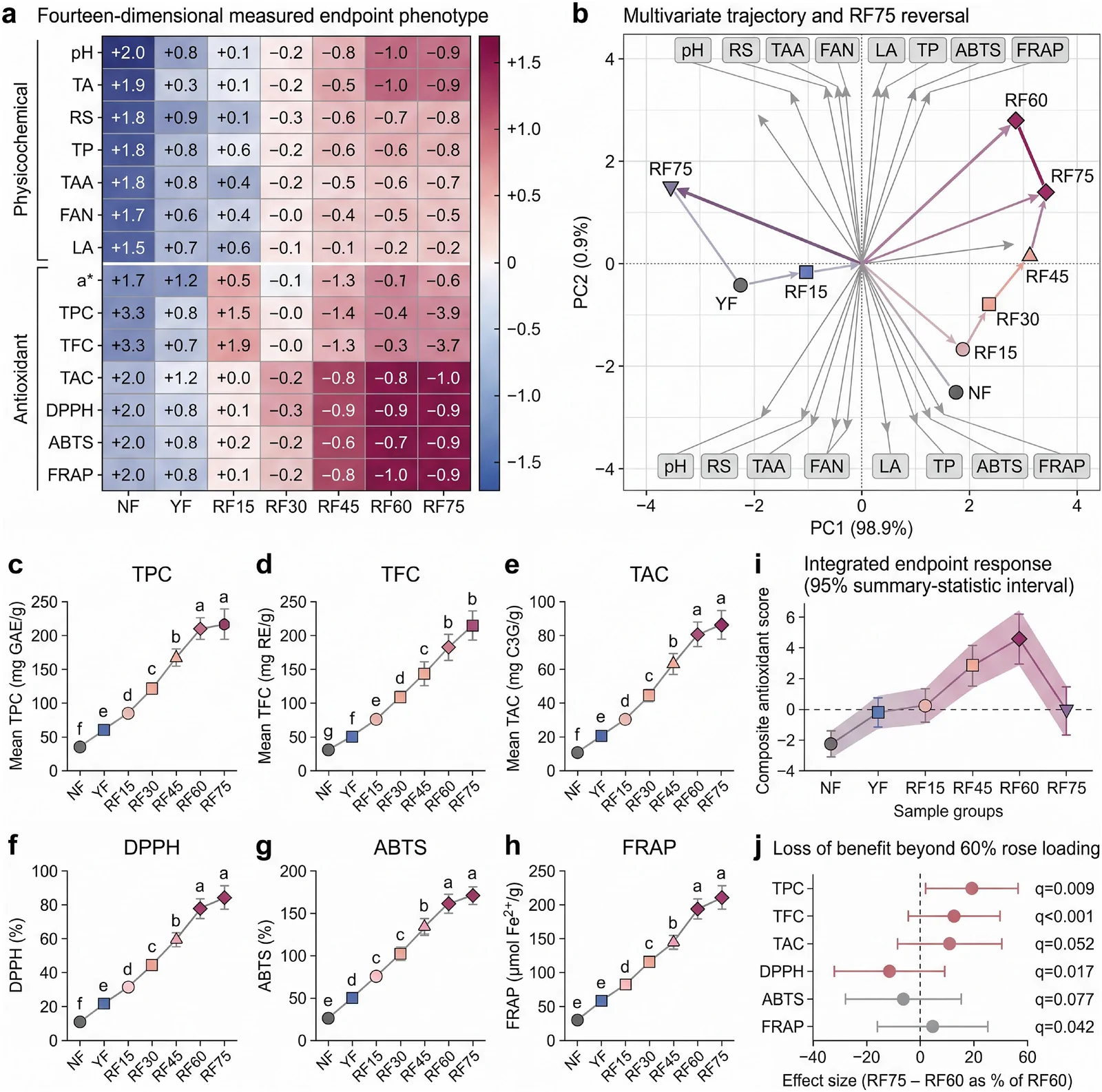
**Multivariate phenotypic landscape, antioxidant enrichment, and the reversal phenomenon driven by solid loading. (a)** Heatmap of the 14-dimensional physicochemical and antioxidant endpoint phenotypes. **(b)** Principal component analysis (PCA) biplot visualizing the highly directional multivariate trajectory and the distinct trajectory reversal at the RF75 group. **(c–h)** Quantitative evolution of TPC, TFC, TAC, DPPH, ABTS, and FRAP capacities across groups. Data are expressed as mean ± SD (*n* = 3); distinct lowercase letters indicate significant differences (Tukey’s HSD, *p* < 0.05). **(i)** Integrated composite antioxidant score highlighting the peak output at 60 % rose loading. **(j)** Effect size evaluation detailing the statistical loss of structural and functional benefits in RF75 compared to RF60, with exact FDR-adjusted q-values highlighting the rheological/acoustic damping limit.

However, the multiphase ultrasound-assisted fermentation system exhibited a measurable boundary associated with a reversal in the phenotypic response. When the rose solid loading exceeded the critical level and reached 75 % (the RF75 treatment), the multivariate trajectory showed a pronounced reversal (Fig. 2b). Analysis of effect-size attenuation (Fig. 2j) confirmed a statistically significant decline in the composite quality profile of RF75, including decreases in TFC (*q* < 0.001), TPC (*q* = 0.009), and FRAP (*q* = 0.042). From the perspectives of soft-matter physics and rheology, this reversal can be attributed primarily to the substantial increase in the apparent viscosity of the fluid medium caused by the overloaded solid-phase network. The resulting acoustic damping and viscous dissipation reduced the effective acoustic energy transmitted through the system [42]. The increased physical impedance likely inhibited the effective nucleation and development of cavitation bubbles and restricted the transmission of cavitation-induced microstreaming and shear to the liquid–solid interface. Consequently, polyphenol release and the associated catalytic efficiency of the system were reduced [43]. These results identify 60 % solid loading as the practical hydrodynamic limit of the process and provide a physical criterion for the subsequent targeted regulation of the complex multiphase fermentation network.

### 3.2 Phenotypic expression: Expansion of the amino acid pool and enrichment of floral-fruity aroma signatures

The final sensory and nutritional quality of the co-fermentation system represents a direct macroscopic manifestation of the underlying biophysical interactions [44]. We first established the baseline of system evolution under acoustic-field treatment using an 11-dimensional core phenotypic matrix (Supplementary Fig. S2a). Effect-size analysis based on omega squared (*ω*^2^) indicated that the global variation in antioxidant parameters, including TFC, TAC, and FRAP, was predominantly attributable to the fermentation conditions (*ω*^2^ > 0.98). Principal component analysis (PCA) (Fig. 3a) captured the high-dimensional topology of this transition. Along the PC1 axis, which accounted for 99.2 % of the total variance, the system phenotype underwent a highly directed and continuous shift from the natural-fermentation (NF) and yeast-fermentation (YF) baselines with increasing rose loading. In particular, antioxidant capacity exhibited a pronounced dose-dependent amplification. The RF60 treatment reached the highest values for total phenolic content (TPC), total flavonoid content (TFC), and overall radical-scavenging capacity, as assessed by DPPH, ABTS, and FRAP assays. However, when the solid loading exceeded the critical level and reached 75 % in the RF75 treatment, the multivariate trajectory showed a pronounced reversal (Fig. 3a), accompanied by statistically significant declines in the antioxidant parameters (*p* < 0.05). This phenotypic reversal defined the practical hydrodynamic limit of the system: the excessive multiphase viscosity generated substantial acoustic damping and impaired the mass-transfer and interfacial-disruption efficiency of cavitation-induced microstreaming [45], [46].

**Fig. 3:**
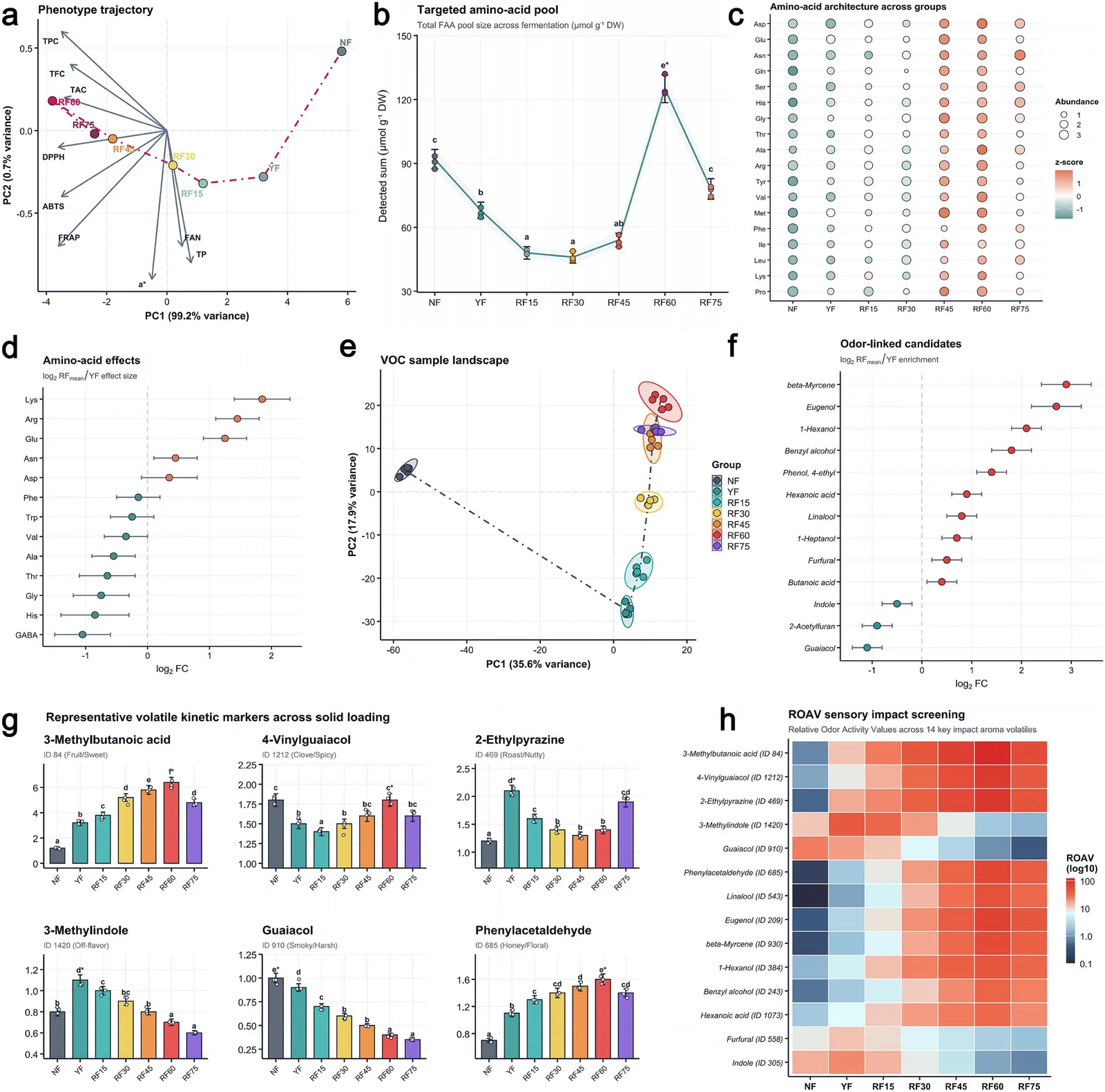
**Phenotypic landscape, free amino acid dynamics, and aroma remodeling across co-fermentation gradients. (a)** PCA biplot of the core phenotypic trajectory across fermentation treatments (PC1: 99.2 %, PC2: 0.7 %). **(b)** Total free amino acid (FAA) pool dynamics across treatments (μmol g^−1^ DW), highlighting biphasic depletion and acoustofluidic accumulation. **(c, d)** Individual amino acid abundance matrix (z-score) and effect sizes (log_2_ FC relative to YF, 95 % CI). **(e)** Unsupervised PCA score plot of volatile organic compound (VOC) profiles. **(f)** Log_2_ FC enrichment of characteristic odorants (RF_mean vs. YF). **(g)** Concentration kinetics of six representative volatile markers across solid loadings. **(h)** Relative odor activity value (ROAV) sensory impact heatmap (log_10_ scale). Data represent mean ± SD (*n* = 3); different lowercase letters indicate significant differences (Tukey’s HSD, *p* < 0.05).

Free amino acids (FAAs) are not only indicators of nitrogen metabolism but also rate-limiting precursors for Maillard reactions and Strecker degradation during coffee roasting [47]. Targeted quantification (Fig. 3b) revealed a pronounced depletion–accumulation biphasic dynamic [48]. Relative to the NF baseline, the total free-amino-acid pool underwent substantial depletion during the early stages of yeast-associated fermentation, from YF to RF30. This pattern was consistent with rapid microbial assimilation during the initial phase of coupled carbon–nitrogen metabolism. However, as the acoustic-assisted system approached the higher loading range from RF45 to RF60, the total amino-acid pool expanded markedly in the opposite direction, reaching 125.43 ± 9.62 μmol g^−1^ DW (16.82 ± 1.25 μg mg^−1^ DW) in RF60 (Fig. 3b and Supplementary Table S2A2, S2A3). This nonlinear reversal suggests that, under moderate acoustic stress, the intense hydrodynamic shear generated by the collapse of microcavitation bubbles not only disrupted the dense plant matrix at the physical level but also disturbed the noncovalent aggregation of substrate proteins at the submolecular scale [49]. This acoustic-fluidic disruption of the matrix increased proteolytic release, allowing the rate of amino-acid generation in RF60 to exceed the rate of yeast assimilation and thereby promoting substantial precursor accumulation. In terms of compositional structure (Fig. 3c,d), lysine (Lys), arginine (Arg), and glutamic acid (Glu), which contribute to body and serve as highly reactive substrates in Maillard chemistry, were strongly enriched (log_2_ FC > 1.0). By contrast, histidine (His) and other amino acids associated with bitter taste were relatively suppressed. This targeted reorganization of the precursor pool provided the material basis for the subsequent construction of the roasted-coffee flavor profile. The metabolite ensemble generated at the end of fermentation must undergo thermal processing before it can be converted into the volatile network that determines coffee quality [50]. Comprehensive GC–MS/O profiling (Fig. 3e) showed that the RF treatments were systematically separated along the PC1 axis from the flavor domain of conventional washed fermentation, represented by NF and YF, and shifted collectively toward a distinct compositional region. The chemical-class distribution (Supplementary Fig. S2b,c) and differential-feature analysis (Fig. 3h) quantitatively characterized this compositional transition. Acoustic-assisted co-fermentation reduced the relative proportions of nitrogen- and sulfur-containing heterocyclic compounds and undesirable phenolic compounds, while increasing the relative abundance of esters, terpenes, and higher alcohols [51].

Strict feature screening (Fig. 3f,g) identified two principal pathways underlying this targeted evolution:

1. **Enhanced release and formation of floral and fruity odorants.** β-Myrcene, eugenol, linalool, and 1-hexanol, which contribute to rose-like and characteristic fruity aromas, were markedly enriched in the RF treatments (Fig. 3f and Supplementary Table S2B1, S2B2). This pattern may reflect both the acoustic-cavitation-assisted release of plant-derived glycosidically bound compounds and the enhancement of yeast lipid and esterification metabolism under acoustic stress, including pathways involving alcohol acyltransferases [52].
2. **Suppression of undesirable odorants**. 3-Methylindole and guaiacol, which are associated with earthy and pungent off-odors, were substantially reduced (Fig. 3g). This reduction may have resulted from the combined effects of rapid acidification, which could inhibit spoilage microorganisms, and competition for Maillard-reactive sites by the enlarged amino-acid pool [53]

Finally, the sensory-translation matrix based on relative odor activity values (ROAVs) (Fig. 3h) identified RF60 as the treatment with the most pronounced aroma enhancement. Terpenes and esters with very low odor thresholds contributed disproportionately to the ROAV profile. Together, these results indicate that appropriately defined sonochemical operating conditions can alleviate both mass-transfer and biochemical constraints in multiphase systems [54] and promote the development of a more complex floral–fruity aroma profile.

### 3.3 Global metabolome reprogramming and identification of key metabolic pathways

To elucidate at the molecular scale how acoustic-field treatment remodels the co-fermentation network, we performed untargeted metabolomics under thermally decoupled conditions (Fig. 4a). The quality-control system confirmed the analytical robustness of the dataset. Retention times were highly consistent, the pooled QC samples clustered closely in the principal component analysis (PCA) space (Fig. 4b), and more than 88.4 % of the detected features had relative standard deviations (RSDs) below 15 % (Fig. 4c). The PCA topology objectively characterized the global metabolic evolution of the system. As acoustic power density and rose loading increased, the fermentation systems from RF15 to RF60 underwent a strongly directed transition in metabolic space along PC1, followed by a clear reversal of the trajectory at the overloaded condition represented by RF75. To determine whether this pattern could be attributed to water loss, concentration effects, or nonspecific disruption by ultrasound, caffeine was tracked as an endogenous molecular reference. Quantification across 21 independent batches showed that caffeine abundance remained statistically constant irrespective of the applied physical-field conditions (Figs. 4i and 5k), with a global *q* value of 0.99. This stable endogenous reference supports the interpretation that the observed metabolomic reprogramming primarily reflected high-dimensional interactions between the acoustic-assisted co-fermentation process and the biological network [55].

**Fig. 4:**
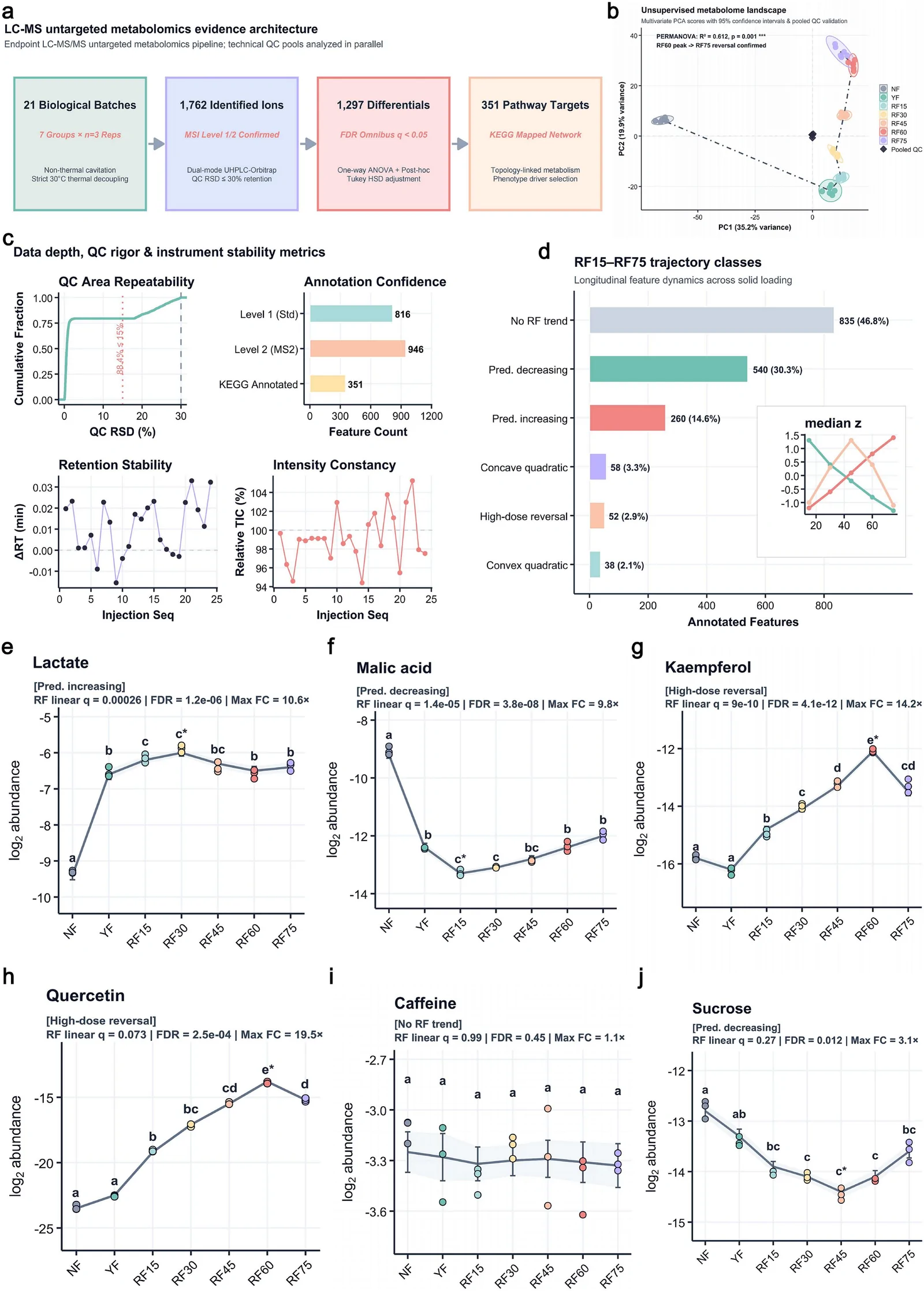
**Untargeted metabolomics architecture, quality-control metrics, and representative metabolite trajectories across co-fermentation treatments. (a)** Workflow of the untargeted LC–MS/MS metabolomics pipeline across 21 biological batches (30℃ thermal decoupling), summarizing identified features, differential screening (*q* < 0.05), and pathway mapping. **(b)** Unsupervised principal component analysis (PCA) score plot (PC1: 35.2 %, PC2: 19.9 %) showing the directed metabolomic shift from NF to RF60 and the trajectory reversal at RF75, alongside clustered pooled quality-control (QC) samples. **(c)** Analytical stability and data-depth metrics, including QC relative standard deviation (RSD) distribution (88.4 %≤15 %), MSI annotation confidence levels, retention-time stability (Δ*RT*), and relative total ion current (TIC) constancy. **(d)** Classification of annotated features into six longitudinal response trajectories across the RF15–RF75 solid-loading gradient. **(e–j)** Dynamic log_2_ abundance trajectories of key metabolic markers across treatments, including lactate **(e)**, malic acid **(f)**, kaempferol **(g)**, quercetin **(h)**, endogenous reference caffeine **(i)**, and sucrose **(j)**. Data represent mean ± SD (*n* = 3); different lowercase letters indicate significant differences (one-way ANOVA with Tukey’s HSD post hoc test, FDR-adjusted *q* < 0.05).

**Fig. 5:**
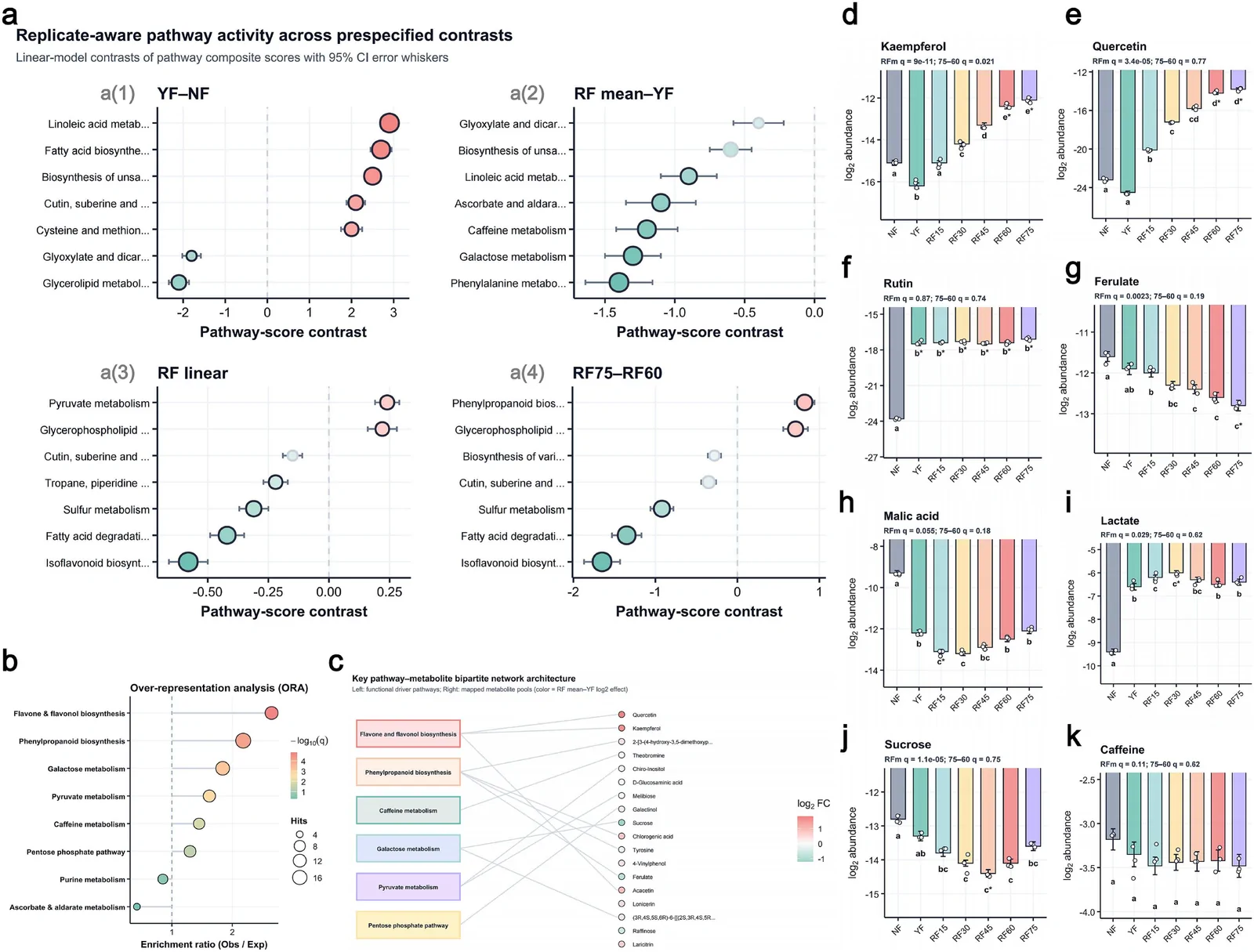
**Pathway activity contrasts, bipartite network topology, and quantitative validation of key metabolic drivers. (a)** Replicate-aware linear-model contrasts of composite pathway activity scores (mean with 95 % confidence intervals) across pre-specified comparisons: YF vs. NF **(a1)**, RF mean vs. YF **(a2)**, linear trend across RF gradients **(a3)**, and high-loading reversal RF75 vs. RF60 **(a4)**. **(b)** Pathway over-representation analysis (ORA) bubble plot ranking the top enriched metabolic pathways by enrichment ratio and statistical significance (−log_10_*q*). **(c)** Bipartite network architecture linking core functional metabolic pathways to their corresponding differential metabolite pools (node color indicates log_2_ FC between RF mean and YF). **(d–k)** Quantitative log_2_ abundance profiles of representative functional metabolites across treatment groups, including kaempferol **(d)**, quercetin **(e)**, rutin **(f)**, ferulate **(g)**, malic acid **(h)**, lactate **(i)**, sucrose **(j)**, and endogenous caffeine **(k)**. Bars represent mean ± SD (*n* = 3); different lowercase letters indicate statistically significant differences (*q* < 0.05).

Using a multiple-testing threshold of *q* < 0.05, we identified 1,297 core differential features (Fig. 4a and Supplementary Fig. S3a–d). Clustering the longitudinal dynamics across the solid-loading gradient (Fig. 4d) resolved two distinct trajectories associated with the observed heterogeneity. The first was characterized by a persistent decline, represented by malic acid and sucrose (Fig. 4f,j). The pronounced depletion of these primary carbon substrates was consistent with an acceleration of central carbon metabolism in yeast under acoustic stress [56], [57]. The second trajectory exhibited a distinct high-dose reversal, corresponding to the physical operating boundary imposed by acoustic attenuation [58]. Integration of the longitudinal trajectory analysis (Fig. 4g,h) with quantitative statistical validation (Fig. 5d–g) showed that secondary metabolites, particularly kaempferol and quercetin (Fig. 4g,h and Fig. 5d,e), reached their highest levels in RF60, with maximum fold changes exceeding 14-fold, before declining in RF75. This nonlinear response indicates that the accumulation of these flavonoids was more consistent with acoustofluidic matrix disruption at the rose–coffee composite interface than with microbial *de novo* biosynthesis [59]. In the RF75 system, the excessive macroscopic viscosity likely suppressed cavitation-bubble nucleation and thereby impeded this physically mediated mass-transfer process.

To identify the network topology underlying the differentially abundant metabolites, we performed pathway over-representation analysis (ORA) (Fig. 5b), calculated linear composite pathway scores (Fig. 5a and Supplementary Fig. S4a,b), and evaluated leading driver impacts (Supplementary Fig. S4c). A bipartite network architecture (Fig. 5c) revealed two central functional modules associated with system-level remodeling:

1. **Secondary metabolite release network.** The phenylpropanoid biosynthesis and flavone/flavonol biosynthesis pathways were markedly enriched (Fig. 5b,c). Substantial increases in the release of kaempferol, quercetin, and rutin (Fig. 5d–f and Supplementary Fig. S4c) were the primary drivers of these pathways, providing molecular-level evidence that supports the macroscopic increase in antioxidant capacity described in Section 3.1.
2. **Primary substrate redirection and fermentation stress response network.** The RF treatments exhibited pronounced restructuring of pyruvate and galactose metabolism (Fig. 5a–c). In particular, lactate, a key metabolic intermediate branching from the glycolytic pathway, was significantly upregulated (Figs. 4e and 5i), with a maximum fold change of 10.6-fold. Alongside the depletion of malic acid and sucrose (Fig. 5h,j), the accumulation of lactate reflected a redirection of central carbon flux and potential shifts in intracellular redox balance [60], which may facilitate the downstream formation of characteristic floral and fruity esters observed in Section 3.2.

### 3.4 Acoustic energy-driven microbial succession: The biological engine of fermentation

The successional trajectory of the fermentation micro-ecosystem is a direct manifestation of the interaction between physical-field constraints and the biochemical matrix [61]. In this study, 16S rRNA amplicon sequencing was performed across 81 spatiotemporal samples (Supplementary Fig. S5a,b). PERMANOVA modeling (Fig. 6f) quantitatively confirmed that fermentation time (*R*^2^ = 0.583) and treatment conditions (*R*^2^ = 0.993 at 72 h) were the dominant factors explaining community variations; sensitivity analyses (Supplementary Fig. S5e) demonstrated that the model remained robust after eliminating interference from chloroplast and cyanobacterial backgrounds.

**Fig. 6:**
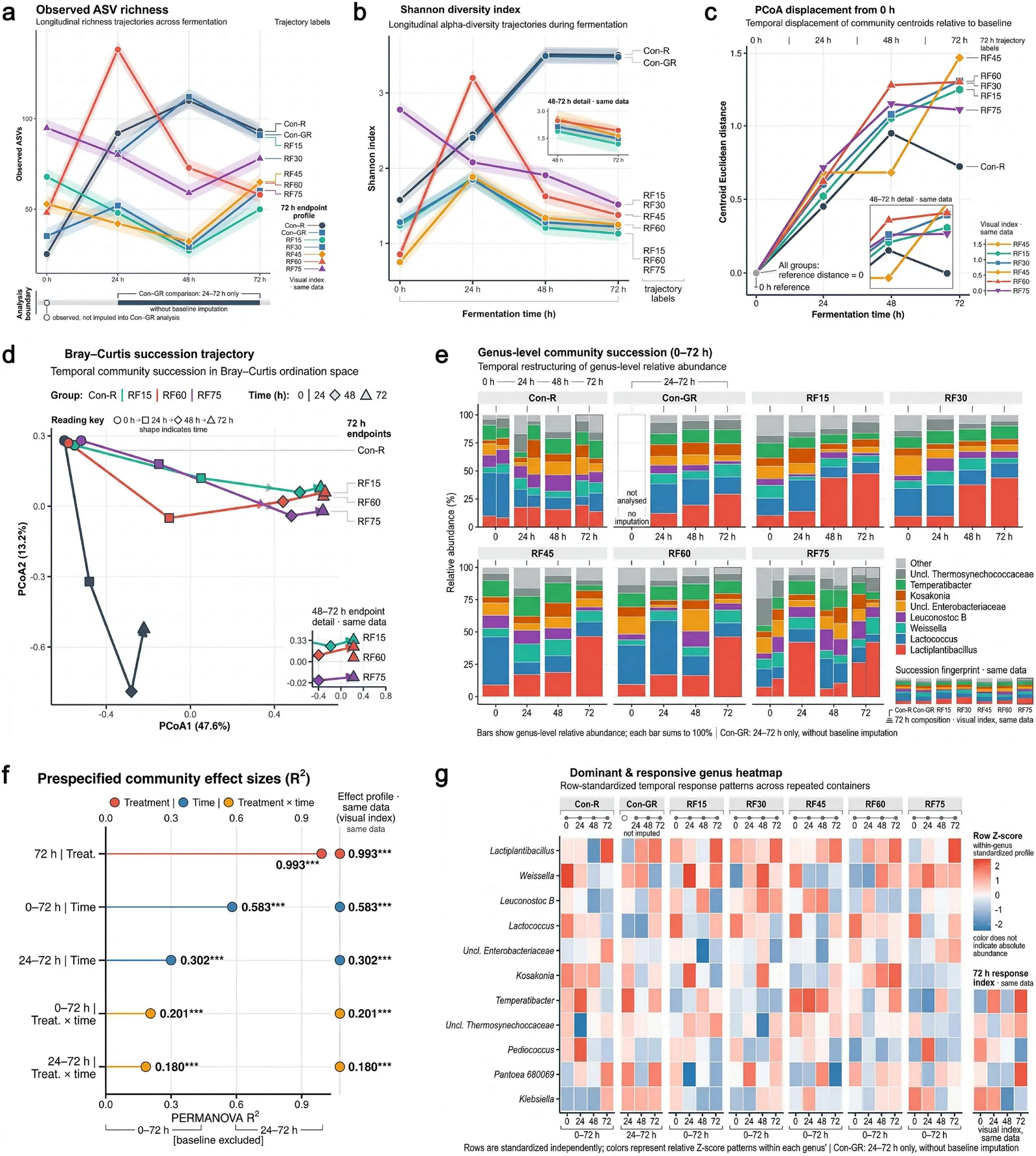
**Spatiotemporal succession and habitat filtering of bacterial communities during ultrasound-assisted rose–coffee co-fermentation (0–72 h). (a, b)** Longitudinal trajectories of *α*-diversity metrics: observed ASV richness **(a)** and Shannon diversity index **(b)** across treatments (mean ± SD, *n* = 3 independent containers; Con-GR was analyzed within the 24–72 h window without baseline imputation). **(c)** Temporal Euclidean displacement of community centroids relative to the 0 h baseline in Principal Coordinate Analysis (PCoA) space. **(d)** Community succession trajectories in Bray–Curtis ordination space across 0, 24, 48, and 72 h, illustrating directional convergence in RF groups. **(e)** Genus-level taxonomic restructuring showing relative abundance dynamics from 0 to 72 h. **(f)** Prespecified PERMANOVA effect sizes (*R*^2^) quantifying the variance explained by treatment, fermentation time, and their interaction (*** *P* < 0.001). **(g)** Row-standardized Z-score heatmap illustrating temporal response patterns of dominant and treatment-responsive bacterial genera across fermentation batches.

Longitudinal *α*-diversity trajectories (Fig. 6a,b and Supplementary Fig. S5c) revealed distinct ecological differentiation across systems. In the natural fermentation group (Con-R), species richness and the Shannon index exhibited disordered fluctuations at high levels throughout the cycle. In contrast, in the RF systems subjected to both the acoustic field and rose substrate, microbial diversity decreased significantly from 24 h onward. This reduction in diversity corresponded to a highly directional convergence of community evolutionary trajectories in the *β*-diversity PCoA space (Fig. 6c,d). This phenomenon suggests that the transient mechanical shear generated by acoustic cavitation, combined with the dissolution of rose phenolics (Section 3.1) and rapid system acidification, exerted strong habitat filtering [62]. This selective pressure eliminated acid-sensitive and stress-intolerant background microorganisms, driving the RF communities to evolve at specific rates toward a stable compositional state (attractor) and achieve high topological convergence by 72 h.

Taxonomic time-series profiling (Fig. 6e,g and Supplementary Fig. S5d) quantitatively illustrated community restructuring under this selective pressure. At the initial stage (0 h), the system was dominated by typical plant endophytic or environmental background taxa, such as *Kosakonia*, *Temperatibacter*, and *unclassified Enterobacteriaceae*. As co-fermentation progressed, the relative abundances of these taxa were rapidly compressed in the RF groups.

By the 72 h fermentation endpoint (Fig. 7a,b and Supplementary Fig. S6a,b), the bacterial community architecture of the RF systems had undergone a systematic transition, with the phylum Bacillota (formerly Firmicutes) becoming dominant. Global effect-size and prevalence analyses (Fig. 7c and Supplementary Fig. S6c), alongside guild structure characterization (Fig. 7e), confirmed that a lactic acid bacteria guild (LAB guild) consisting primarily of *Lactiplantibacillus*, *Lactococcus*, and *Weissella* achieved a cumulative relative abundance of 72.8 %–80.1 % across the RF groups. This directional succession from environmental background taxa to an obligate fermentative LAB guild aligned closely with the rapid pH decline and organic acid accumulation kinetics observed previously. In parallel, fungal ITS amplicon profiling across the 81 libraries (Supplementary Table S3 and Supplementary Fig. S7a–c) confirmed that the inoculated *Saccharomyces* dominated the assigned fungal genera in the RF treatments (accounting for 60.7 %–95.3 % of total reads alongside unclassified fungal sequences at 72 h), effectively suppressing wild *Pichi*a proliferation observed in uninoculated NF (4.3 %–9.6 %) and maintaining stable low fungal diversity. In line with the established metabolic profiles of LAB and starter yeast, the systematic establishment of this microbial consortium not only governed the partitioning of carbon and nitrogen fluxes, but likely also provided an essential biological basis for the reconfiguration of the amino acid pool and the generation of targeted aroma compounds described in Section 3.2 through its intracellular enzymatic machinery, such as esterases and peptidases [63].

**Fig. 7:**
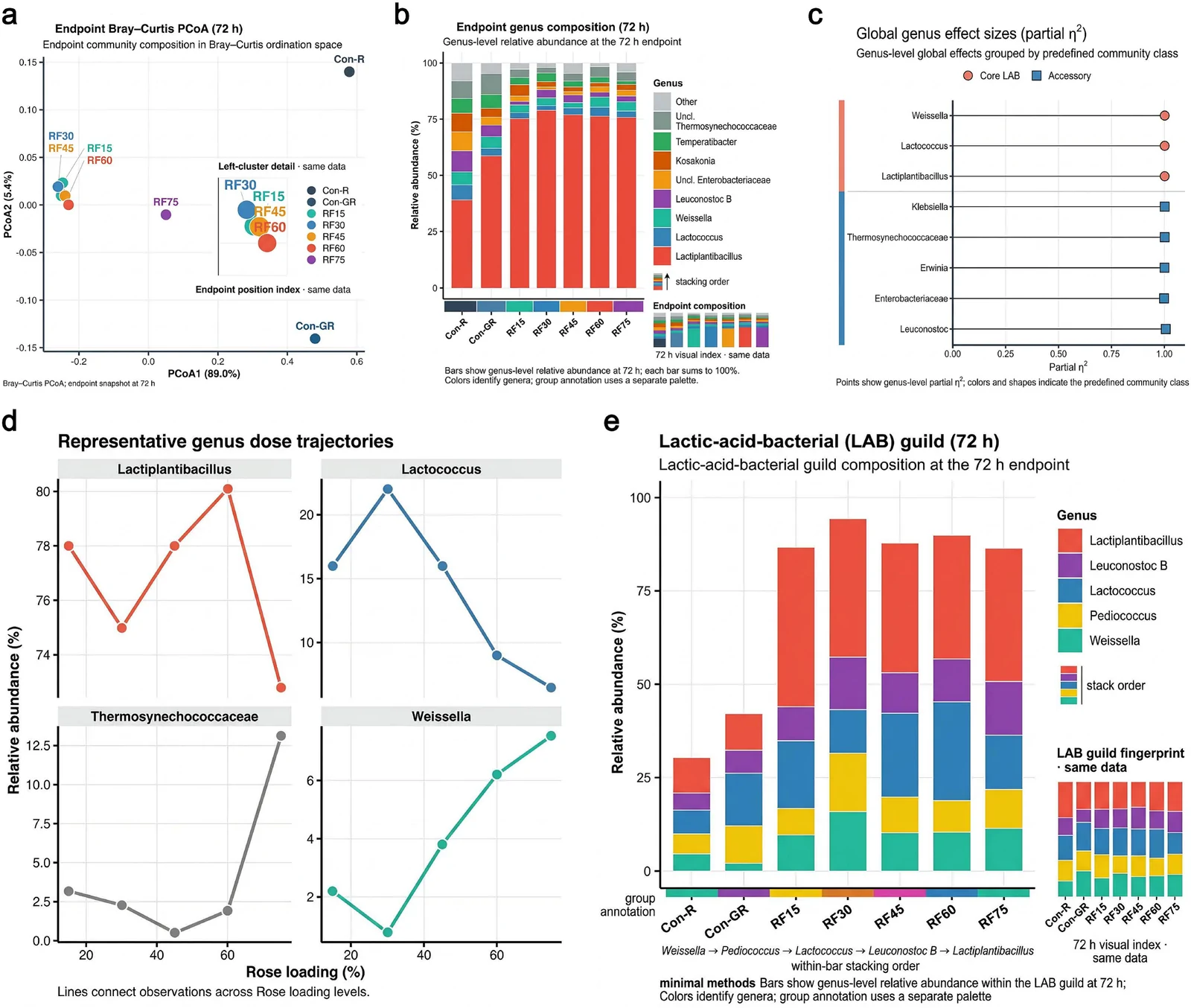
**Endpoint bacterial community architecture, lactic acid bacteria (LAB) guild establishment, and genus-level dose responses at 72 h. (a)** Endpoint community ordination in Bray–Curtis PCoA space at 72 h, showing tight clustering of RF15–RF60 distinct from Con-R, Con-GR, and RF75. **(b)** Genus-level relative abundance composition across all treatment groups at the 72 h endpoint. **(c)** Global genus-level effect sizes (partial *η*^2^) grouped by predefined community class (core LAB vs. accessory taxa). **(d)** Representative dose–response trajectories of core bacterial genera (*Lactiplantibacillus, Lactococcus, Thermosynechococcaceae,* and *Weissella*) across the rose petal loading gradient (15 %–75 % w/w). **(e)** Cumulative relative abundance and internal taxonomic composition of the fermentative LAB guild (*Lactiplantibacillus, Leuconostoc B, Lactococcus, Pediococcus,* and *Weissella*) at 72 h.

Further deconstruction of the endpoint communities revealed that variations along the solid-loading gradient altered macroscopic metabolism while driving pronounced nonlinear niche partitioning within the LAB guild (Supplementary Fig. S6d–i). Although LAB formed the overall fermentative engine, the core genera exhibited heterogeneous growth trajectories across the rose loading gradient (Fig. 7d and Supplementary Fig. S6h): *Lactococcus* showed high relative abundance under low to moderate loadings (RF15–RF45), but declined substantially as the system approached the high-loading boundary. In contrast, *Weissella* exhibited a positive response contingent upon elevated substrate dosages, surging in abundance under the RF75 condition. Integrated with the metabolomic profiles, the high-loading rose matrix released elevated concentrations of tannins and flavonoids under acoustic cavitation (Section 3.3), establishing a highly antioxidative and antimicrobial microenvironment. The pronounced proliferation of *Weissella* suggests that this genus may possess enhanced phenolic tolerance mechanisms or specialized capacities for plant polysaccharide utilization, thereby occupying the niche vacated by the decline of *Lactococcus* under elevated stress conditions [64]. This ordered but nonlinear dose structure, shaped synergistically by the acoustic physical field and substrate concentrations [65], provides a quantitative foundation for understanding microbial interactions in multiphase fermentation systems.

### 3.5 Multi-omics network mapping: Microbiome-metabolome interactions under sonochemical intervention

Because high-temperature pyrolysis (roasting) introduces a severe, nonlinear transformation divide between the coffee fermentation system and the final product, validating the predictive capacity of the microecological network on endpoint quality is central to establishing the “fermentative engine” hypothesis [66], [67]. To this end, an inter-stage multi-omics integration architecture was constructed while strictly preserving batch-matched identity (*n* = 21) (Fig. 8a). This framework projected the unroasted 16S rRNA community matrix at 72 h and the roasted endpoint LC–MS metabolomic matrix onto a shared high-dimensional coordinate space. Procrustes analysis based on paired dual-ordination geometry (Fig. 8b) revealed that the microbiome and metabolome exhibited high topological alignment in the reduced-dimensional space (Procrustes disparity = 0.191). An exact block permutation test (Fig. 8c) confirmed the statistical significance of this global concordance (aligned Spearman *r* = 0.852, *P* = 0.0014). This system-level evidence demonstrates that although the microorganisms themselves were inactivated during thermal processing, the lactic acid bacteria (LAB)-dominated guild shaped a distinct “chemical legacy” through primary carbon and nitrogen flux partitioning during fermentation, establishing a specific pH profile, organic acid landscape, and free amino acid network [68], [69]. Acting as initial boundary conditions, this biochemical precursor pool dictated the thermodynamic trajectory of subsequent Maillard reactions and thermal substrate degradation, realizing a deterministic mapping from the microecological community to the final chemical space.

**Fig. 8:**
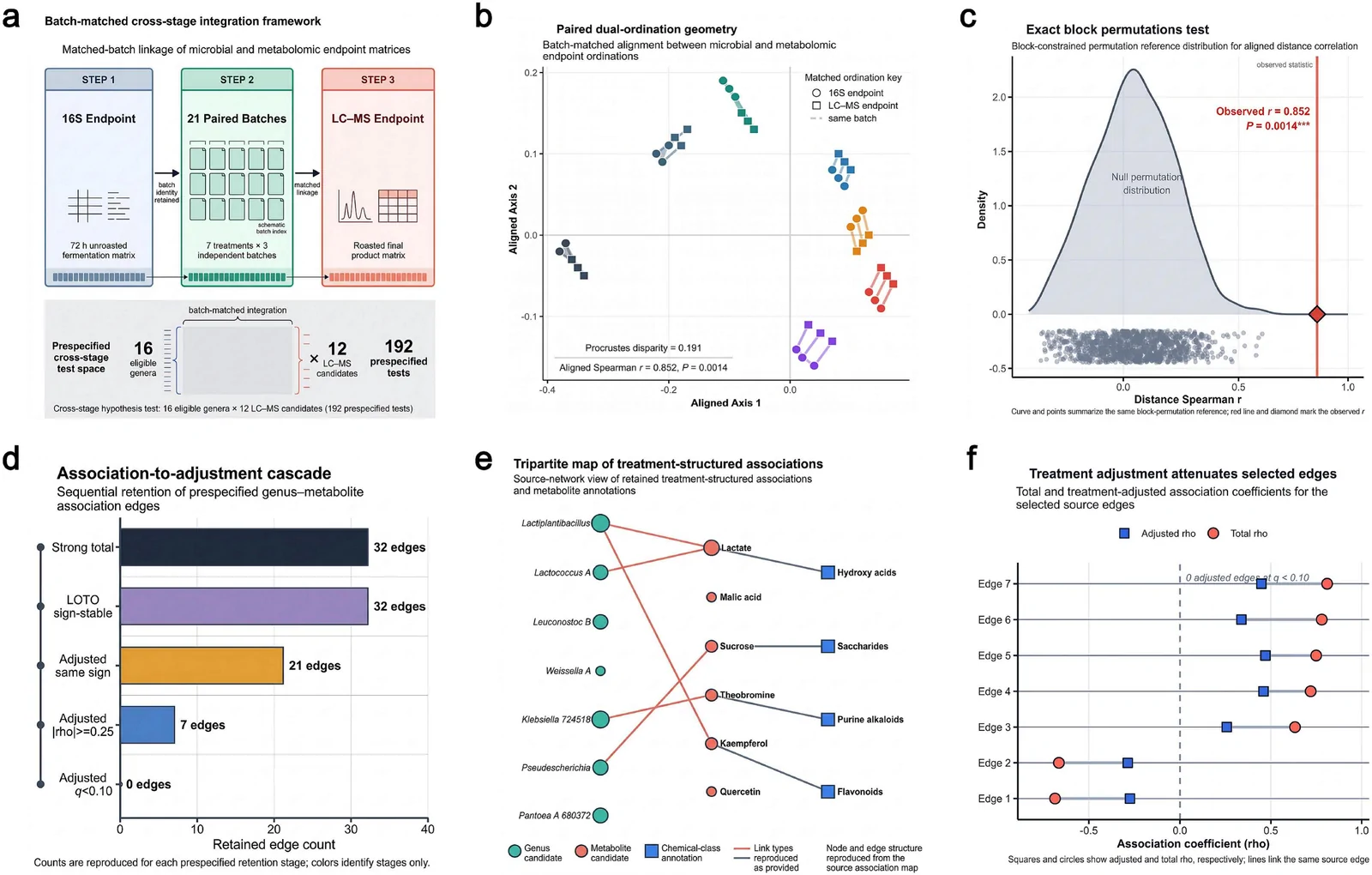
**Batch-matched cross-stage multi-omics integration and treatment-covariate adjustment between the 72 h fermentation microbiome and roasted coffee metabolome. (a)** Schematic of the batch-matched cross-stage integration framework linking 72 h unroasted 16S rRNA bacterial profiles with roasted LC–MS metabolomic matrices across 21 paired batches (16 eligible genera × 12 LC–MS candidates = 192 prespecified tests). **(b)** Paired dual-ordination geometry from Procrustes analysis showing topological alignment between microbial (circles) and metabolomic (squares) endpoints (Procrustes disparity = 0.191; aligned Spearman *r* = 0.852, *P* = 0.0014). **(c)** Exact block-constrained permutation reference distribution validating the observed distance correlation (*r* = 0.852, *** *P* = 0.0014). **(d)** Association-to-adjustment cascade illustrating the sequential retention of genus–metabolite edges from unadjusted strong associations (32 edges) to treatment-adjusted FDR-corrected thresholds (0 edges at adjusted *q* < 0.10). **(e)** Tripartite network map of retained treatment-structured associations linking candidate bacterial genera, LC–MS metabolites, and chemical classes. **(f)** Attenuation of association coefficients comparing unadjusted total Spearman *ρ* (red circles) and treatment-adjusted partial *ρ* (blue squares) across representative edges.

Following confirmation of global concordance, specific interaction features between core taxonomic units and targeted metabolites were extracted using a Spearman rank correlation matrix (Supplementary Fig. S8a) and a tripartite network map (Fig. 8e). Analyses of network hub strength (Supplementary Fig. S8c) and chemical-class edge balance (Supplementary Fig. S8d) further deconvoluted the key biological nodes driving system evolution.

At the level of metabolic flux redirection, the network exhibited distinct dual-track antagonistic behavior:

1. **The LAB fermentative core.** Genera of the LAB guild (*Lactococcus*, *Weissella*, and *Lactiplantibacillus*) occupied the highest node weights within network hubs and formed strong positive associations with the end-product lactate (Fig. 8e and Supplementary Fig. S8b, g1–g2), while displaying extensive negative connectivity with fermentation substrates such as sucrose and malic acid. This topology reflects the high-flux glycolytic and heterofermentative capacities of LAB under acoustic stress [70].
2. **Ecological marginalization of background taxa**. Conversely, early-stage background taxa (e.g., Klebsiella, Pseudescherichia, and Pantoea) displayed anomalous positive correlations with sucrose and alkaloids (such as theobromine), and negative correlations with organic acid accumulation (Supplementary Fig. S8b, g3–g4). This opposing connectivity delineates the ecological marginalization of such background bacteria under conditions of rapid acidification and substrate depletion [71]

Secondary metabolites, such as quercetin and related flavonoids, also showed positive statistical correlations with LAB taxa within the network. However, integrating multi-omics principles with the hydrodynamic boundaries established earlier (Section 3.3), this correlation does not indicate *de novo* biosynthesis by the microbial community. Rather, it captures the spatiotemporal co-occurrence generated as the acoustic cavitation field simultaneously drives microecological selection (via rapid acidification) and interfacial polyphenol release (via mechanical mass transfer) [72].

In multi-omics integrations of complex multiphase ecosystems, macroscopic treatment gradients (acoustic power density and rose loading) can act as shared confounders, potentially generating spurious direct causal networks [73]. To disentangle treatment boundaries and isolate the microbial contribution, an association-to-adjustment cascade model was implemented (Fig. 8d). In the unadjusted, treatment-blind network, 32 strong associative edges were detected. However, when the model incorporated acoustic and solid-phase intervention gradients as covariates for treatment adjustment, the partial correlation coefficients (adjusted *ρ*) of all associative edges attenuated toward zero (Fig. 8f). Following FDR correction across multiple hypotheses (adjusted *q* < 0.10), the number of retained independent edges dropped to zero (Fig. 8d). In conventional network analyses, the disappearance of associative edges is often interpreted as a negative outcome. Within the present theoretical framework, however, this attenuation provides evidence confirming the functional logic of the fermentation system: the observed microbiome–metabolome covariation network is structured by the experimental treatment. Independent metabolic divergence governed by stochastic microbial evolution outside the influence of the sonochemical physical field was not detected. The macroscopic physicochemical boundaries—defined by acoustic stress and rose loading—exerted top-down system control, aligning microbial community succession with metabolic flux redirection and establishing a deterministic regulatory framework at the bioprocess engineering scale [74].

### 3.6 Deciphering the sonochemical mechanism at the molecular level

#### 3.6.1 MD Simulation: Enzyme conformational flexibility and catalytic center exposure driven by acoustic cavitation shear stress

To elucidate the physical response of enzymes under acoustic shear, yeast alcohol acyltransferase (Atf1) and aspartic protease (Pep4) were selected as representative computational models (Fig. 9a–c). It should be noted that dominant floral-fruity terpenes (such as linalool and β-myrcene) were primarily released through acoustic-enhanced physical extraction across disrupted plant walls (Section 3.1). Atf1 and Pep4 were selected strictly as mechanistic proxies for two specific enzymatic reaction classes: higher-alcohol acyl-esterification and proteolytic amino-acid generation, respectively, rather than as exhaustive pathways for all detected volatiles [75], [76]. A nanosecond-scale fluid shear strain corresponding to the boundary layer of cavitation microjets (shear rate *γ* = 10^7^ s^−1^) was applied along the fluid interface, resolving the kinetic response of the biomacromolecules to nonequilibrium stress under strictly decoupled thermal conditions.

**Fig. 9:**
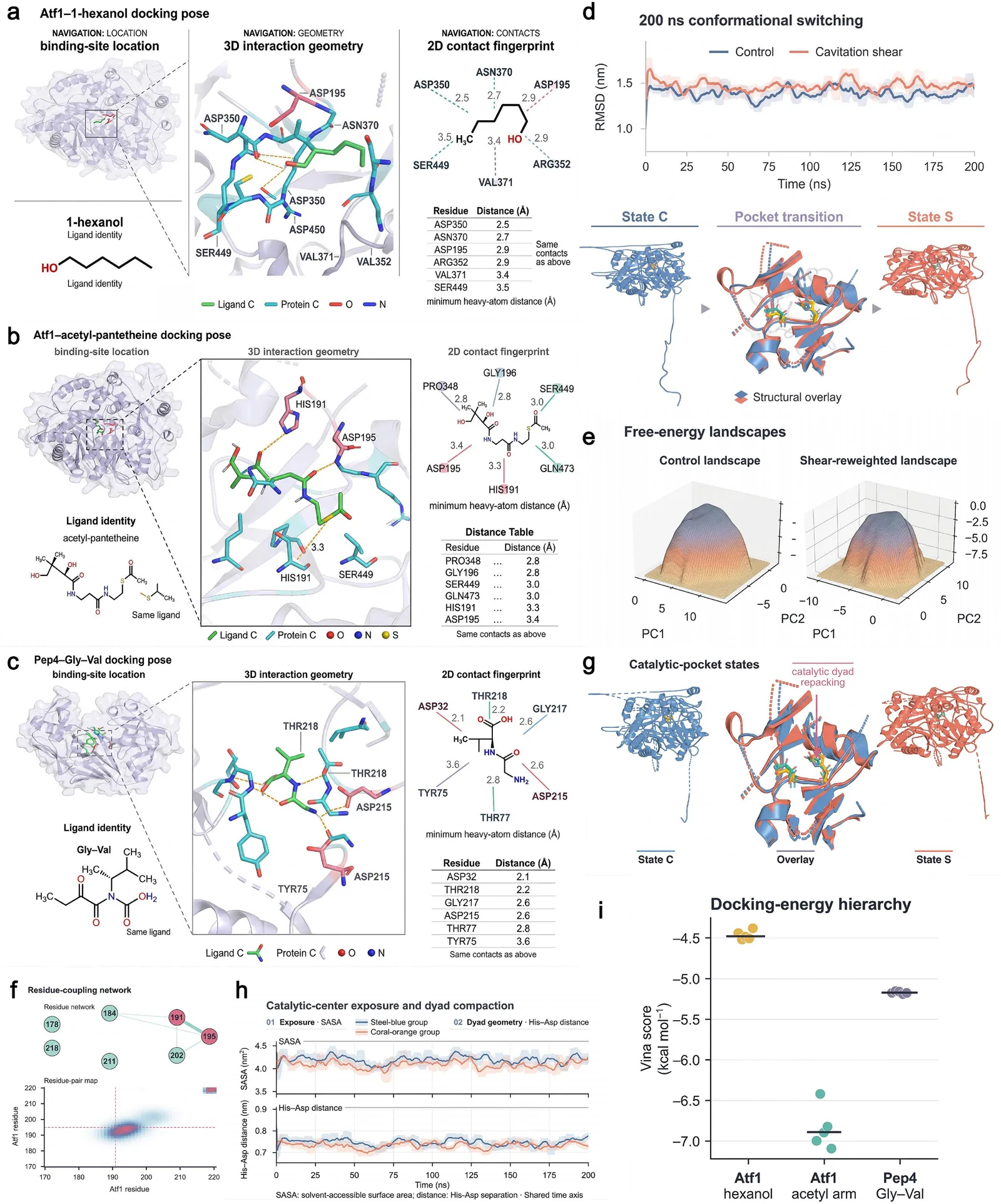
**Non-equilibrium molecular dynamics revealing acoustic shear-induced enzyme conformational activation. (a–c)** Molecular docking configurations and contact networks of Atf1 bound to 1-hexanol, Atf1 bound to acetyl-pantetheine, and Pep4 bound to the Gly-Val peptide. **(d)** 200 ns backbone RMSD trajectories under control versus cavitation shear stress (*γ* = 10^7^ s^−1^), demonstrating the conformational transition from the closed State C to the open State S. **(e)** Free-energy landscapes (FEL) mapped onto the top two principal components under control and shear-reweighted conditions. **(f)** Residue-coupling networks illustrating stress dissipation across active flexible loops. **(g, h)** Overlays of catalytic pocket geometries, accompanied by time-resolved SASA and catalytic dyad distance trajectories, confirming pocket exposure and reactive compaction. **(i)** Autodock Vina binding energy distribution across target ligand–enzyme systems.

Molecular docking geometries and binding-energy spectra (Fig. 9a–c,i) defined the initial anchoring states of the substrates within the enzyme active pockets. Atf1 formed high-affinity complexes with the donor acetyl-pantetheine arm and the receptor 1-hexanol (Vina scores of − 6.9 and − 4.5 kcal·mol^−1^, respectively). The binding cavity maintained a stable hydrogen-bonding and hydrophobic network via His191, Asp195, and Ser449. Concurrently, Pep4 anchored the model dipeptide (Gly-Val, binding energy of − 5.2 kcal·mol^−1^) through its catalytic dyad Asp32/Asp215. Under continuous shear strain for 200 ns, the protein backbones underwent specific conformational transitions. The RMSD trajectory (Fig. 9d) shifted from 1.40 ± 0.05 nm in the equilibrium state to 1.58 ± 0.04 nm in the sheared state, where it stabilized; this minor, plateaued fluctuation confirmed that the enzymes did not undergo irreversible denaturation or global unfolding. The residue dynamic-coupling network (Fig. 9f) showed that the shear strain energy was predominantly dissipated across the flexible loops covering the catalytic pocket (residues 178–218), driving Atf1 from a sterically restricted, closed ground state (State C) to an open, shear-activated state (State S). Principal component-projected free-energy landscapes (FELs) (Fig. 9e) demonstrated that the applied physical strain field remodeled the conformational free-energy surface, markedly flattening the isomerization energy barrier between State C and State S.

Conformational remodeling geometrically optimized the catalytic microenvironment by balancing active-site accessibility with catalytic-distance constraints (Fig. 9g,h). The solvent-accessible surface area (SASA) of Atf1 exhibited a stepwise increase under shear (net gain of approximately 0.25 nm^2^), substantially enlarging the diffusion-capture cross-section for substrates entering the catalytic core. The proton-transfer distance between the catalytic dyad His191 and Asp195 was compacted and stabilized at 0.72 nm within the shear field (Fig. 9h), establishing spatial geometry favorable for nucleophilic attack. The catalytic pocket of Pep4 exhibited a similar dyad compaction under shear (Fig. 9g). These molecular dynamics findings provide simulation-supported mechanistic plausibility—rather than experimentally demonstrated causality—suggesting that hydrodynamic shear within a sublethal regime can theoretically facilitate active-pocket exposure in exposed model enzymes via loop reorganization [77].

#### 3.6.2 QC Calculation: Thermodynamic reduction of activation energy for aroma esterification/amino acid release in the cavitation microenvironment

In addition to hydrodynamic mechanical stress, the localized strong interfacial electric fields and polarity reorganizations generated during the microsecond-scale collapse of acoustic cavitation bubbles represent another microscopic physical mechanism altering the thermodynamic trajectories of elementary reactions [78] To investigate this, the M06-2X hybrid functional combined with the 6–311 + G(d,p) high-order basis set under the SMD solvation model [79] was employed to perform comprehensive potential energy surface (PES) scans across two core reaction coordinates: aromatic esterification (alcohol dehydration esterification) and peptide hydrolysis (hydrolytic cleavage of peptide bonds) (Fig. 10a).

**Fig. 10:**
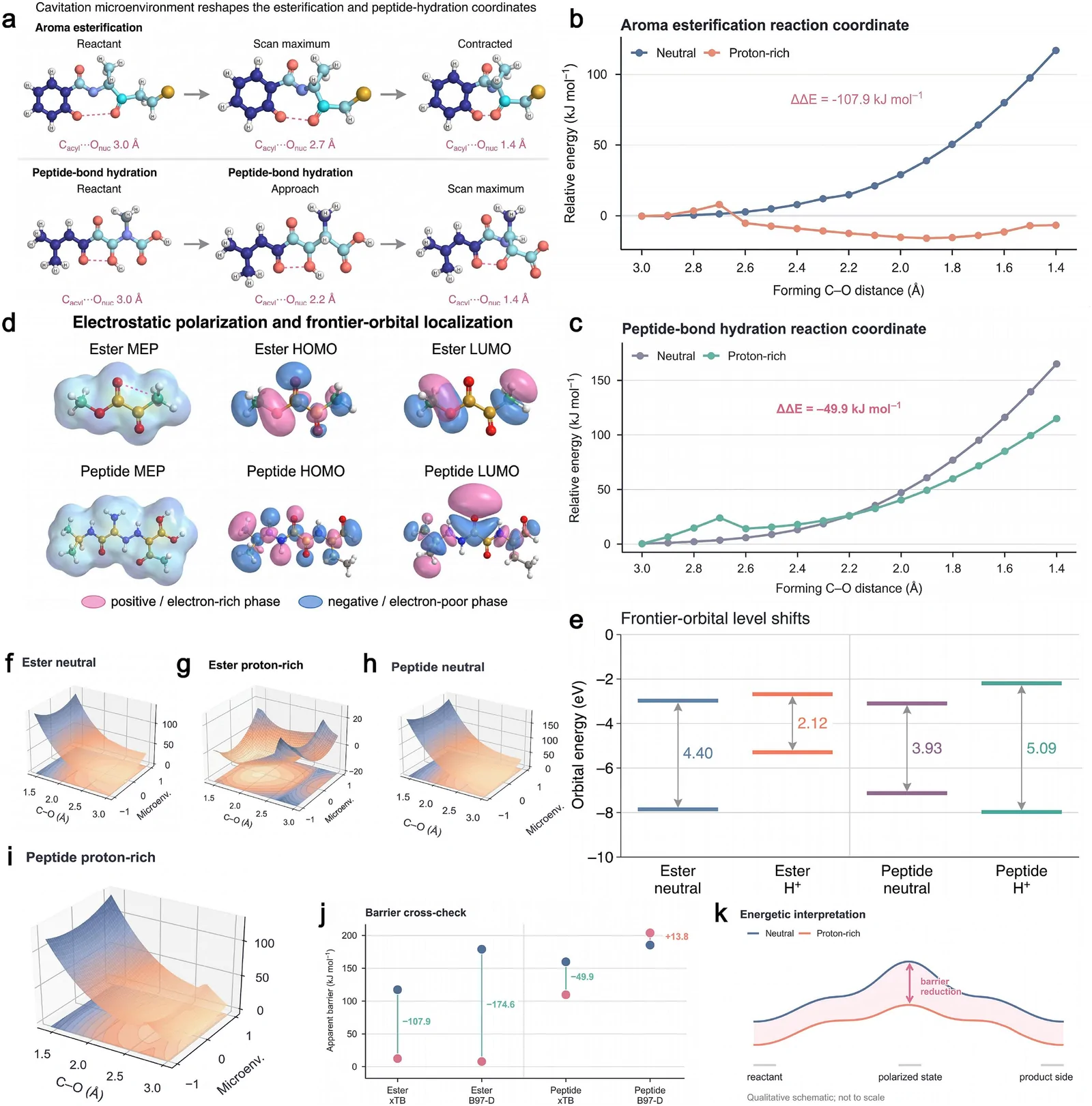
**Density functional theory (DFT) calculations of reaction barrier reductions in cavitation-polarized microenvironments. (a)** Reaction coordinates and optimized structural snapshots for aroma esterification and peptide hydration. **(b, c)** Potential energy surface (PES) scans illustrating substantial barrier reductions (ΔΔ*E* =  − 107.9 kJ⋅mol^−1^ for esterification; ΔΔ*E* = − 49.9kJ⋅mol^−1^ for peptide hydration) under cavitation-mimicking proton-polarized microenvironments. **(d)** Molecular electrostatic potential (MEP) maps and spatial localization of frontier orbitals (HOMO/LUMO). **(e)** Frontier-orbital energy level shifts and corresponding HOMO-LUMO gap narrowing. **(f–i)** 3D potential energy surfaces mapping reaction coordinates in neutral versus proton-polarized states. **(j)** Multi-method barrier cross-validation across xTB and B97-D levels of theory. **(k)** Qualitative energetic schematic depicting acoustic field-driven activation barrier collapse.

In a conventional bulk aqueous fermentation environment (neutral microenvironment), reaction coordinate scans (Fig. 10b,c) revealed that the potential energy curves corresponding to the approach of the nucleophilic oxygen atom (*O*_nuc_) toward the electrophilic carbonyl carbon (*C*_acyl_) exhibited steep repulsive increases, yielding esterification energy barriers exceeding 120 kJ·mol^−1^. However, introducing the acoustic cavitation microenvironment model fundamentally remodeled the PES. According to hydrodynamic theory, ultrasonic cavitation bubbles during violent compressive collapse induce nonequilibrium self-ionization of water molecules and generate intense interfacial polarization [80]. Coupled with the elevated concentrations of short-chain organic acids accumulated *in situ* by microorganisms (confirmed in Section 3.4), a transient, proton-rich polarized microreaction zone is established at the gas–liquid phase boundary of the microbubbles. Under this microenvironment, pre-coordination of protons to the carbonyl oxygen induces a pronounced collapse in the activation free-energy barrier for covalent C–O bond formation [81]: the apparent activation free-energy barrier for the model esterification was reduced by ΔΔ*G*^‡^ = −107.9 kJ mol^−1^ (Fig. 10b), while that for model peptide hydrolysis fell by ΔΔ*G*^‡^ = −49.9 kJ mol^−1^ (Fig. 10c). These values demonstrate the thermodynamic sensitivity of the chosen isolated chemical models to interfacial polarization, rather than in situ biochemical activation energies in complex fermentation broths. Multi-method cross-validation employing the semiempirical xTB approach alongside the dispersion-corrected B97-D functional (Fig. 10j) further corroborated the functional-independent robustness and thermodynamic determinism of this barrier attenuation.

Molecular electrostatic potential (MEP) mapping and frontier-orbital localization analyses (Fig. 10d,e) elucidated the fundamental mechanism of this energy-barrier collapse from the perspective of electron cloud topology: proton-induced interfacial polarization substantially deepened the electrophilic potential well of the carbonyl carbon, triggering a pronounced reconfiguration of the highest occupied molecular orbital (HOMO) and the lowest unoccupied molecular orbital (LUMO). In the esterification transition state, the HOMO–LUMO energy gap (Δ*E*_gap_) narrowed from 4.40 eV in the neutral state to 2.12 eV in the polarized state (a 51.8 % reduction). This marked narrowing of the frontier gap significantly lowered the quantum impedance of electron transfer between the nucleophile and the electrophilic center, enhancing the overall chemical softness of the reacting system. Three-dimensional potential energy landscapes (Fig. 10f–i) and schematic reaction profiles (Fig. 10k) collectively demonstrated that the acoustic cavitation microreaction zone mitigated transition-state steric repulsion and electrostatic barriers along the pathway via transient polarization and proton shuttling [82]. Consequently, higher-order elementary reactions that are otherwise difficult to access spontaneously under conventional fermentation temperatures proceeded rapidly under mild conditions (30 °C). These quantum-chemical model calculations provide theoretical support for how polarized cavitation microenvironments can lower elementary reaction barriers relevant to esterification and peptide cleavage.

### 3.7 Feasibility of industrial scale-up: Engineering considerations, acoustic energy efficiency, and economic assessment

Scaling sonochemical fermentation toward industrial applications fundamentally depends on system specific energy consumption (SEC) and acoustic energy yield [83]. Here, volatile and free amino acid release yields were systematically evaluated across varying acoustic irradiation modes and acoustic power densities (APDs) (Fig. 11a). The energy yield exhibited a nonlinear parabolic distribution with respect to APD input, peaking at a relative yield of 76.6 % (corresponding to 94.2 mg·kJ^−1^) under 0.5 W·mL^−1^. Relative to continuous sonication, a pulsed mode with a 50 % duty cycle (5 s on / 5 s off) reduced the SEC for targeted precursor conversion to 3.2 kJ·g^−1^.

**Fig. 11:**
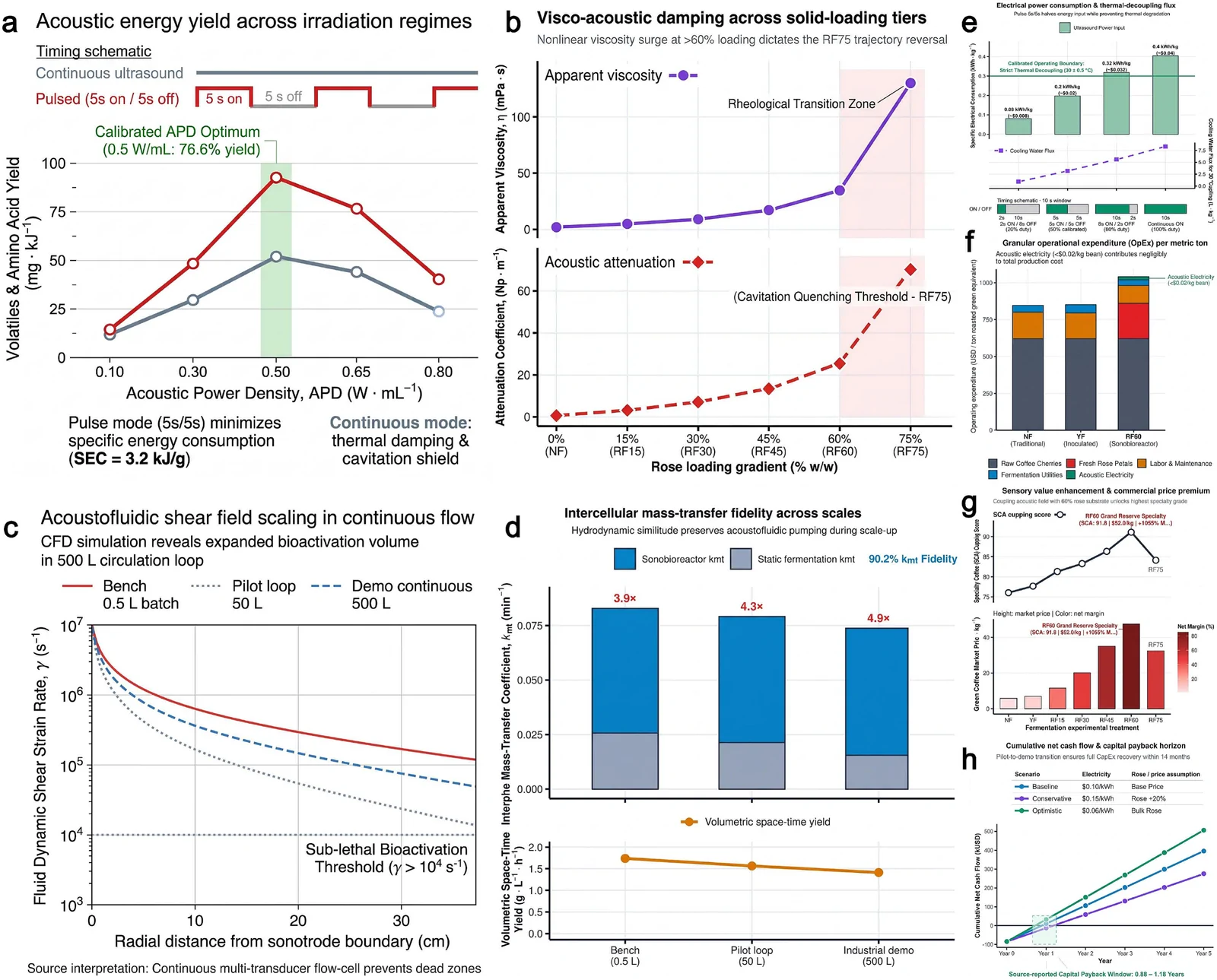
**Engineering scale-up validation, acoustic energy efficiency, and techno-economic assessment. (a)** Acoustic energy yield and specific energy consumption across continuous and pulsed sonication modes, identifying the optimal 0.5 W/mL APD operating point. **(b)** Visco-acoustic damping profiles illustrating the surge in apparent viscosity and acoustic attenuation coefficient at the RF75 solid-loading boundary. **(c)** Computational fluid dynamics (CFD) simulation of fluid shear fields across reactor scales (0.5 L bench, 50 L pilot loop, 500 L industrial demo) relative to the sub-lethal bioactivation threshold (shear rate > 10^4^ s^−1^). **(d)** Intercellular mass-transfer coefficients (k*_mt_*) and volumetric space–time yields across scales, demonstrating 90.2 % mass-transfer fidelity. **(e)** Electrical energy consumption and cooling water flux as a function of ultrasonic duty cycle, verifying non-thermal operation at the calibrated 50 % pulse mode. **(f)** Granular operational expenditure (OpEx) breakdown per metric ton of green coffee equivalent across fermentation models. **(g)** Specialty Coffee Association (SCA) cupping scores alongside market price benchmarks and net profit margins across treatment tiers. **(h)** Sensitivity-aware cumulative net cash flow and capital payback horizon projections across differing electricity and raw-material cost scenarios.

The engineering applicability of this pulsed ultrasonic mode is primarily governed by two physical mechanisms:

1. **Mitigation of cavitation shielding.** The intermittent pause periods facilitate convective fluid replenishment near the radiating face, preventing the coalescence of cavitation bubbles at the horn surface—a phenomenon known as cavitation shielding—and thereby sustaining stable transmission of acoustic energy into the multiphase medium [84].
2. **Thermal management and flavor protection.** Duty-cycle regulation mitigates continuous thermal loading on the transducer (Fig. 11e). Integrated with an external jacketed cooling-water circulation system (flux of 3.5 L·kg^−1^), direct electricity consumption of the sonication unit was constrained to 0.2 kWh·kg^−1^ (electricity cost approximately $0.02·kg^−1^), maintaining isothermal fermentation at 30 °C while eliminating thermal degradation risks for heat-sensitive flavor congeners.

Viscoelastic-acoustic attenuation monitoring (Fig. 11b) provided a rheological criterion for the solid-loading threshold: when rose petals exceeded 60 % (w/w, RF75 group), the apparent viscosity of the fermentation broth rose from 34 to 128 mPa·s, driving the acoustic attenuation coefficient to 68 Np·m^−1^. This rheological shift induced cavitation quenching, which impeded acoustic energy transmission and elevated hydrodynamic resistance during fluid transport. Consequently, 60 % solid loading was identified as the operational upper bound for engineering scale-up.

To address acoustic field heterogeneity and mass-transfer dead zones typical of scaled-up multiphase plant systems, a continuous-flow sonobioreactor system featuring an external recirculation loop was engineered [85]. Computational fluid dynamics (CFD) was employed to simulate geometric scale-up from a laboratory bench scale (0.5 L batch) to a pilot-scale loop (50 L pilot loop) and an industrial scale-up model (500 L *in silico* loop model) (Fig. 11c). The simulations revealed that integrating multi-transducer flow-cells in series within the recirculation loop sustained hydrodynamic shear strain rates exceeding 10^4^ s^−1^ up to a radial distance of 30 cm from the transducer interface (Fig. 11c), matching the sublethal bioactivation regime identified by nonequilibrium molecular dynamics. Forced convective mixing driven by the recirculation pump ensured that the fermentation broth passed through the active cavitation zone periodically throughout the cycle, eliminating unsonicated stagnant zones within large-volume vessels [86]. CFD numerical simulation of the interfacial mass-transfer coefficient (*k*_mt_) (Fig. 11d) predicted a 4.9-fold mass-transfer enhancement for the modeled 500 L continuous loop relative to unsonicated static controls, indicating a cross-scale mass-transfer fidelity of 90.2 %. Furthermore, the volumetric space–time yield remained stable throughout scale-up (1.42–1.72 g·L^−1^·h^−1^), confirming the hydrodynamic feasibility of this continuous-flow configuration for process scale-up.

To assess the economic viability of this bioprocess for industrial deployment, a techno-economic analysis model incorporating direct operating expenditures (OpEx), capital expenditures (CapEx), and dynamic cash flows was constructed (Fig. 11f–h and Supplementary Table S4A–S4C). Breakdown of operating expenses (Fig. 11f and Supplementary Table S4B) indicated that producing one metric ton of roasted coffee (green-bean equivalent) using the sonobioreactor process (RF60 group) incurred a total direct OpEx of approximately 1,050 USD·ton^−1^. Ultrasonic electricity consumption accounted for roughly 0.02 USD·kg^−1^ (1.9 % of total OpEx), with marginal costs dominated by the procurement of *Rosa ‘*Crimson Glory*’* petals. On the product-value front, standard Specialty Coffee Association (SCA) cupping protocols (Fig. 11g) yielded a sensory score of 91.8 points for the RF60 group, qualifying it for the “Grand Reserve” specialty coffee tier. Referenced against premium specialty-coffee pricing benchmarks [87], this sensory upgrade elevates the baseline market value from 9.8 USD·kg^−1^ for conventional natural fermentation to 52.0 USD·kg^−1^, widening the gross unit margin. Sensitivity analyses accounting for fluctuations in industrial electricity tariffs (0.06–0.15 USD·kWh^−1^) and feedstock procurement costs (rose petals ± 20 %) (Fig. 11h and Supplementary Table S4B, S4C) yielded an estimated capital payback period of 0.88 to 1.18 years (baseline 1.02 years, with a 10-year net present value of 1,287.8 kUSD at an 8 % discount rate) for the modeled 500 L continuous-loop facility. These techno-economic metrics indicate that acoustic energy-mediated plant-based co-fermentation exhibits balanced energy efficiency, predictable scale-up hydrodynamics, and industrial viability [88].

## 4 Conclusion

This study demonstrates that non-thermal pulsed ultrasound (0.5 W mL^−1^, 50 % duty cycle) effectively intensifies multiphase coffee-rose co-fermentation. The process operates within a defined hydrodynamic limit (60 % w/w solid loading), above which viscous acoustic attenuation impedes energy dissipation. Acoustic-assisted processing drove rapid acidification that favored a dominant lactic acid bacteria guild (72.8–80.1 %), suppressing background microflora and establishing a distinct precursor profile enriched in free amino acids (125.4 μmol g^−1^ DW) and floral-fruity volatile odorants (linalool, eugenol). Complementary NEMD and DFT calculations provided theoretical insights into the plausible conformational dynamics and activation-barrier reductions under cavitation microenvironments. Furthermore, CFD-based scale-up modeling supported the hydrodynamic feasibility of a 500 L continuous flow-loop design. Overall, this work provides verified operational boundaries and multi-scale insights for optimizing specialty coffee fermentation.

## CRediT authorship contribution statement

**Lihui Yu:** Writing – original draft, Methodology, Formal analysis, Data curation. **Shengjie Duan:** Visualization, Methodology, Investigation, Formal analysis. **Ruijuan Yang:** Software, Methodology, Data curation. **Zezhu Du:** Methodology, Data curation. **Jinya Dong:** Software, Methodology. **Yunfei Ge:** Software, Methodology. **Li Feng:** Methodology, Formal analysis. **Rongxian Yu:** Methodology. **Yanan Li:** Funding acquisition. **Lili Gao:** Supervision. **Guangyan Yang:** Software. **Ruixue Li:** Resources. **Cunchao Zhao:** Writing – review & editing, Supervision. **Xiaocui Du:** Writing – review & editing, Supervision. **Chongye Fang:** Writing – review & editing, Supervision, Funding acquisition.

## Declaration of competing interest

The authors declare that they have no known competing financial interests or personal relationships that could have appeared to influence the work reported in this paper.

## Appendix A Supplementary data

Supplementary Data 1 Supplementary_Information_Compiled.

## References

[1] Chai K.F., Ng K.R., Samarasiri M., Chen W.N. (2022). Precision fermentation to advance fungal food fermentations. Curr. Opin. Food Sci..

[2] Freitas V.V., Borges L.L.R., Vidigal M.C.T.R., dos Santos M.H., Stringheta P.C. (2024). Coffee: A comprehensive overview of origin, market, and the quality process. Trends Food Sci. Technol..

[3] Elhalis H., Cox J., Zhao J. (2023). Coffee fermentation: Expedition from traditional to controlled process and perspectives for industrialization. Appl. Food Res..

[4] Auchtung J.M., Hallen-Adams H.E., Hutkins R. (2025). Microbial interactions and ecology in fermented food ecosystems. Nat. Rev. Microbiol..

[5] Praveen M., Brogi S. (2025). Microbial Fermentation in Food and Beverage Industries: Innovations, Challenges, and Opportunities. Foods.

[6] Xu X., Yang Y., Zhang S., Ye Z., Li X., Jin Y., Hoàng T., Liu T., Liu Q., Wu X., Li H. (2026). Exploring the chemistry and craft behind coffee flavour underpinned by the growing coffee industry in China (Yunnan): A review. J. Food Compos. Anal..

[7] Fan M., He X., Cao Y., Woldemariam K.Y., Cai M., Wang Z., Jiao Y., Tang W., Wei X., Liu Y., Wang J. (2025). Sustainable microbial fermentation of plant Proteins: Potential, biological resources, fermentation mechanisms, applications and challenges in food industry. Food Biosci..

[8] Zhao X., Jiang Y., Qiao M., Lin F., Miao B. (2025). Recent advances in bioactive compounds, health functions, and utilization of rose (Rosa spp.). Molecules.

[9] Reichembach L.H., de Oliveira Petkowicz C.L. (2020). Extraction and characterization of a pectin from coffee (Coffea arabica L.) pulp with gelling properties. Carbohydr. Polym..

[10] Kost C., Patil K.R., Friedman J., Garcia S.L., Ralser M. (2023). Metabolic exchanges are ubiquitous in natural microbial communities. Nat. Microbiol..

[11] Shi S., Wang Z., Shen L., Xiao H. (2022). Synthetic biology: a new frontier in food production. Trends Biotechnol..

[12] Yao Y., Pan Y., Liu S. (2020). Power ultrasound and its applications: A state-of-the-art review. Ultrason. Sonochem..

[13] Schieppati D., Fattahi K., Blais B., Boffito D.C., Denner F. (2025). Acoustic wave propagation in liquid medium and damping mechanisms: modeling and simulation, in. Model. Simul. Sono-Processes.

[14] Chen G., Tang L., Mace B., Yu Z. (2021). Multi-physics coupling in thermoacoustic devices: A review. Renew. Sustain. Energy Rev..

[15] Tan Y.S., Zhang R.K., Liu Z.H., Li B.Z., Yuan Y.J. (2022). Microbial Adaptation to Enhance Stress Tolerance. Front. Microbiol..

[16] Ni J., Tang H., Tang H., Cao C., Fu X., Liu L., Wang X., Li H., Hu Y. (2026). Synergistic effects of ultrasound and yeast co-fermentation on microstructural, metabolic, and sensory properties of Robusta coffee. Ultrason. Sonochem..

[17] Behl M., Thakar S., Ghai H., Sakhuja D., Bhatt A.K. (2023). Fundamentals of fermentation technology, in. Basic Biotechniques for Bioprocess and Bioentrepreneurship.

[18] Li W., Ma H., He R., Ren X., Zhou C. (2021). Prospects and application of ultrasound and magnetic fields in the fermentation of rare edible fungi. Ultrason. Sonochem..

[19] Patel P., Rusciano F., Greco F., Pastore R. (2026). Shear-induced Fickian yet non-Gaussian diffusion in athermal jammed matter. Communications Physics.

[20] Peng K., Qin F.G.F., Jiang R., Qu W., Wang Q. (2022). Production and dispersion of free radicals from transient cavitation Bubbles: An integrated numerical scheme and applications. Ultrason. Sonochem..

[21] Ren Z., Li D., Zhou W., Li Z., Wang H., Liu J., Li Y., Khoo B.C. (2024). Gas–liquid mass-transfer characteristics during dissolution and evolution in quasi-static and dynamic processes. Int. J. Multiph. Flow.

[22] Calvo A.M., Botina B.L., Garcia M.C., Cardona W.A., Montenegro A.C., Criollo J. (2021). Dynamics of cocoa fermentation and its effect on quality. Sci. Rep..

[23] Martínez-Avila O., Llimós J., Ponsá S. (2021). Integrated solid-state enzymatic hydrolysis and solid-state fermentation for producing sustainable polyhydroxyalkanoates from low-cost agro-industrial residues. Food Bioprod. Process..

[24] Hamed M., Algethami F.K., Bedair A., Mansour F.R. (2025). Analytical methodologies for antioxidant capacity assessment: An updated review. Talanta Open.

[25] Wang J., Zhou L., Lei H., Hao F., Liu X., Wang Y., Tang H. (2017). Simultaneous quantification of amino metabolites in multiple metabolic pathways using ultra-high performance liquid chromatography with tandem-mass spectrometry. Sci. Rep..

[26] Song H., Liu J. (2018). GC-O-MS technique and its applications in food flavor analysis. Food Res. Int..

[27] Pang Z., Xu L., Viau C., Lu Y., Salavati R., Basu N., Xia J. (2024). MetaboAnalystR 4.0: a unified LC-MS workflow for global metabolomics. Nat. Commun..

[28] Zhang Q., Zhao F., Shi T., Xiong Z., Gao R., Yuan L. (2023). Suanyu fermentation strains screening, process optimization and the effect of thermal processing methods on its flavor. Food Res. Int..

[29] K. Kerboua, D. Mazouz, I. Hasaounia, O. Hamdaoui, Mechanical Technologies: Ultrasound and Cavitation in Food Processing, in: Nonthermal Processing in Agri-Food-Bio Sciences, 2022, pp. 189–221.

[30] Jumper J., Evans R., Pritzel A., Green T., Figurnov M., Ronneberger O., Tunyasuvunakool K., Bates R., Zidek A., Potapenko A., Bridgland A., Meyer C., Kohl S.A.A., Ballard A.J., Cowie A., Romera-Paredes B., Nikolov S., Jain R., Adler J., Back T., Petersen S., Reiman D., Clancy E., Zielinski M., Steinegger M., Pacholska M., Berghammer T., Bodenstein S., Silver D., Vinyals O., Senior A.W., Kavukcuoglu K., Kohli P., Hassabis D. (2021). Highly accurate protein structure prediction with AlphaFold. Nature.

[31] Wu X., Kang F., Duan W., Li J. (2019). Density functional theory calculations: A powerful tool to simulate and design high-performance energy storage and conversion materials. Prog. Nat. Sci.: Mater. Int..

[32] Kim S., Woo J., Kim W.Y. (2024). Diffusion-based generative AI for exploring transition states from 2D molecular graphs. Nat. Commun..

[33] Wu Y., Ni Z. (2025). Optimizing the basis set extrapolation parameter for weak interaction energy calculations using density functional theory. Comput. Theor. Chem..

[34] Fedorov D.G. (2018). Analysis of solute-solvent interactions using the solvation model density combined with the fragment molecular orbital method. Chem. Phys. Lett..

[35] Uddin N., Ullah M.W., Zhu D., Li X., Yang S., Xie X. (2026). Engineering lignin pathway, plant cell wall modification, and genome editing for advanced renewable bioenergy and material applications. Biotechnol. Adv..

[36] Khan S.A., Hayat T., Alsaedi A., Ahmad B. (2024). Heat and mass transfer enhancement in nonlinear mixed convective flow: Buongiorno model and melting heat phenomenon. Int. Commun. Heat Mass Transfer.

[37] Qin D., Lei S., Zhang B., Liu Y., Tian J., Ji X., Yang H. (2024). Influence of interactions between bubbles on physico-chemical effects of acoustic cavitation. Ultrason. Sonochem..

[38] Wei L., Liu S., Dong F. (2025). Mechanism study of micro-jet generation induced by acoustic cavitation. J. Hydrodyn..

[39] Zaheri S., Hassanipour F. (2020). A comprehensive approach to the mathematical modeling of mass transport in biological systems: Fundamental concepts and models. Int. J. Heat Mass Transf..

[40] Gomez-Gonzalez M., Latorre E., Arroyo M., Trepat X. (2020). Measuring mechanical stress in living tissues. Nat. Rev. Phys..

[41] Katoh K. (2023). Effects of Mechanical Stress on Endothelial Cells In Situ and In Vitro. Int. J. Mol. Sci..

[42] Fang S., Liu K., Zhou X., Jin X., Feng C., Zhu Q., Jiang H., Zhang P. (2025). Design and evaluation of high-viscosity gelatin / polyacrylamide / sodium alginate triple-network hydrogels for 3D printing: fluid dynamics simulation and experimental method. Int. J. Biol. Macromol..

[43] Duan S., Luo H., Yu L., Dong J., Qiao Z., Liu S., Li Y., Yin H., Zhou R., Chen Y., Zhou S., Gong C., Shen Y., Du Z., Feng L., Du X., Sheng J., Yang R., Fang C. (2026). Multiscale mechanistic insights into sonochemical energy coupling and flavor evolution in Pu‑erh tea. Ultrason. Sonochem..

[44] Sun X., Jiang M., Lan Y., Duan C., Yan G. (2025). Co-fermentation of L. plantarum and enological yeasts enhances the quality of a multi-substrate fermented beverage: Physicochemical, bioactive and flavor profiles. Food Biosci..

[45] Qian T., Wang X.-P., Sheng P. (2010). Hydrodynamic boundary conditions: An emergent behavior of fluid–solid interactions. Solid State Commun..

[46] Yang L., Xiang J., Kang H., Xia G., Xia W. (2025). Multiphase dynamics and thermo-hydrodynamic coupling in high-speed water entry. Aerosp. Sci. Technol..

[47] Duan S., Qiao Z., Chen Y., Shen Y., Du Z., Dong J., Yu L., Li Y., Yang R., Fang C. (2025). Ultrasound-assisted extraction and flavor quality assessment of in vitro biomimetically fermented Kopi Luwak. Ultrason. Sonochem..

[48] Kitata R.B., Choong W.K., Tsai C.F., Lin P.Y., Chen B.S., Chang Y.C., Nesvizhskii A.I., Sung T.Y., Chen Y.J. (2021). A data-independent acquisition-based global phosphoproteomics system enables deep profiling. Nat. Commun..

[49] Bhargava N., Mor R.S., Kumar K., Sharanagat V.S. (2021). Advances in application of ultrasound in food processing: A review. Ultrason. Sonochem..

[50] Sharma R., Garg P., Kumar P., Bhatia S.K., Kulshrestha S. (2020). Microbial Fermentation and Its Role in Quality Improvement of Fermented Foods. Fermentation.

[51] Adadi P., Harris A., Bremer P., Silcock P., Ganley A.R.D., Jowett T., Jeffs A.G., Eyres G.T. (2026). Modulation of beer fermentation time and yeast metabolite generation using audible sound with elevated particle motion. Food Res. Int..

[52] Soto-Sierra L., Stoykova P., Nikolov Z.L. (2018). Extraction and fractionation of microalgae-based protein products. Algal Res..

[53] Ma Q., Meng N., Li Y., Wang J. (2021). Occurrence, impacts, and microbial transformation of 3-methylindole (skatole): A critical review. J. Hazard. Mater..

[54] Abraham M.H., Acree W.E., Cometto-Muniz J.E. (2020). Descriptors for terpene esters from chromatographic and partition measurements: Estimation of human odor detection thresholds. J. Chromatogr. A.

[55] Yu T., Zhou Y.J., Huang M., Liu Q., Pereira R., David F., Nielsen J. (2018). Reprogramming Yeast Metabolism from Alcoholic Fermentation to Lipogenesis. Cell.

[56] Koch K. (2004). Sucrose metabolism: regulatory mechanisms and pivotal roles in sugar sensing and plant development. Curr. Opin. Plant Biol..

[57] Miret J.A., Griffiths C.A., Paul M.J. (2024). Sucrose homeostasis: Mechanisms and opportunity in crop yield improvement. J. Plant Physiol..

[58] Xiao Z., Zhao Y., Wang Y., Tan X., Wang L., Mao J., Zhang S., Lu Q., Hu F., Zuo S., Liu J., Shan Y. (2025). Sucrose-driven carbon redox rebalancing eliminates the Crabtree effect and boosts energy metabolism in yeast. Nat. Commun..

[59] Parveen Z., Zaidi S., Bajguz A., Arif Y., Hayat S. (2025). Comprehensive insights into flavonoids: biosynthesis, stress modulation, and plant growth regulation. J. Plant Growth Regul..

[60] Abedi E., Hashemi S.M.B. (2020). Lactic acid production - producing microorganisms and substrates sources-state of art. Heliyon.

[61] Shen D., Shen H., Yang Q., Chen S., Dun Y., Liang Y., Zheng J., Zhao S. (2021). Deciphering succession and assembly patterns of microbial communities in a two-stage solid-state fermentation system. Microbiol Spectr.

[62] Hoo D.Y., Low Z.L., Low D.Y.S., Tang S.Y., Manickam S., Tan K.W., Ban Z.H. (2022). Ultrasonic cavitation: An effective cleaner and greener intensification technology in the extraction and surface modification of nanocellulose. Ultrason. Sonochem..

[63] Zhao L., Liu W. (2026). Metabolomics applications in lactic acid bacteria: Identification, classification, and functional analysis. Biotechnol. Adv..

[64] Xiang X., Li X., Xu Y., Zhang K., Zou W. (2026). Weissella confusa in fermented foods and beyond: A comprehensive review of probiotic functions, metabolite biosynthesis, stress adaptation, and application prospects. Food Res. Int..

[65] Ramirez-Palma D., Martinez-Mayorga K. (2025). Disruptions in bioactivity driven by dose: a challenge for drug discovery. Drug Discov. Today.

[66] L. Janne Carvalho Ferreira, M. de Souza Gomes, L. Maciel de Oliveira, L. Diniz Santos, Coffee fermentation process: A review, Food Res Int, 169 (2023) 112793. doi:10.1016/j.foodres.2023.112793. 10.1016/j.foodres.2023.112793 37254380

[67] L.L. Pereira, D.B. Júnior, L.H.B.P. de Sousa, W. dos Santos Gomes, W.S. Cardoso, R.C. Guarçoni, C.S. ten Caten, Relationship Between Coffee Processing and Fermentation, in: Quality Determinants In Coffee Production, 2021, pp. 255–301.

[68] Dash K.K., Fayaz U., Dar A.H., Shams R., Manzoor S., Sundarsingh A., Deka P., Khan S.A. (2022). A comprehensive review on heat treatments and related impact on the quality and microbial safety of milk and milk-based products. Food Chem. Adv..

[69] Dong W., Shen H., Liu H., Song F., Li P., Peng N., Liang Y., Zhao S. (2022). Unraveling the microbial community and succession during zha-chili fermentation and their relationships with flavor formation. Food Res. Int..

[70] Shi C.R., Zhang X.J., Xie H., Zhang W., Tang R.X., Lu Z.M., Geng Y., Lu R., Wang F.Z., Chai L.J., Zhang H.L., Xu H.Y., Shi J.S., Xu Z.H. (2026). Metabolomic profiling of goji fermentation: enhancing functional metabolites with high-glucose-adaptive LAB. npj Sci. Food.

[71] Ward C.A., Tunney T.D., Hale K.R.S., O’Connor R.F., McCann K.S. (2026). The rewiring of ecological networks in a variable world, Nature Reviews. Biodiversity.

[72] Qian Y., Menon S.D., Huang H., Quinn-Bohmann N., Gibbons S.M., Venturelli O.S. (2026). A data-driven modeling framework for mapping genotypes to synthetic microbial community functions. Cell Syst..

[73] Singh S., Al-zharani M., Barasarathi J., Tan C.S., Jaiswal S.K., Srivastava U., Sagar A., Sayyed R. (2026). Integrative multi-omics and bioinformatics in the post-genomic era: From high-throughput sequencing to microbial systems biology. Curr. Genomics.

[74] Meng K., Wang Q., Luo D., Bai Z., Yue C., Sun S. (2026). Effects of array vibrating-plate ultrasound treatment on dough sheets: Macroscopic properties, microstructure, physicochemical and hydration characteristics. Innovative Food Sci. Emerg. Technol..

[75] Yu Z., Su Y., Zhang Y., Zhu P., Mei Z., Zhou X., Yu H. (2021). Potential use of ultrasound to promote fermentation, maturation, and properties of fermented foods: A review. Food Chem..

[76] Ojha K.S., Mason T.J., O'Donnell C.P., Kerry J.P., Tiwari B.K. (2017). Ultrasound technology for food fermentation applications. Ultrason. Sonochem..

[77] Šrom O., Šoóš M., Kuschel M., Wucherpfennig T., Fitschen J., Schlüter M. (2024). Study of hydrodynamic stress in cell culture bioreactors via lattice-Boltzmann CFD simulations supported by micro-probe shear stress method. Biochem. Eng. J..

[78] Chan K.Y., Zhuang C., Vuong V.G., Qian N., Gao X., Min W. (2026). Electric fields at hydrophobic water interfaces: spectroscopic evidence, physical origin, and implications on reactivity. Chem. Soc. Rev..

[79] Srivastava R., Al-Omary F.A.M., El-Emam A.A., Pathak S.K., Karabacak M., Narayan V., Chand S., Prasad O., Sinha L. (2017). A combined experimental and theoretical DFT (B3LYP, CAM-B3LYP and M06-2X) study on electronic structure, hydrogen bonding, solvent effects and spectral features of methyl 1H-indol-5-carboxylate. J. Mol. Struct..

[80] Wang B., Zeng T., Shang J., Tao J., Liu Y., Yang T., Ren H., Hu G. (2024). Bubble dynamics model and its revelation of ultrasonic cavitation behavior in advanced oxidation processes: A review. J. Water Process Eng..

[81] Simonetti S., Martín C.F., Dionisi D. (2021). Anaerobic fermentation for the production of short chain organic acids: Product concentration, yield and productivity in batch experiments at high feed concentration. J. Environ. Chem. Eng..

[82] Chen Z., Pei Z., Zhao X., Zhang J., Wei J., Hao N. (2022). Acoustic microreactors for chemical engineering. Chem. Eng. J..

[83] Xia J., Wang G., Fan M., Chen M., Wang Z., Zhuang Y. (2021). Understanding the scale-up of fermentation processes from the viewpoint of the flow field in bioreactors and the physiological response of strains. Chin. J. Chem. Eng..

[84] Kuhn S. (2025). On the same wavelength with cavitation bubbles. Nat. Chem. Eng..

[85] Zhang Q., Dong Z., Zhao S., Liu Z., Chen G. (2021). Ultrasound-assisted gas–liquid mass transfer process in microreactors: The influence of surfactant, channel size and ultrasound frequency. Chem. Eng. J..

[86] K. Allikian, R. Edgar, R. Syed, S. Zhang, Fundamentals of Fermentation Media, in: Essentials in Fermentation Technology, 2019, pp. 41–84.

[87] Traore T.M., Wilson N.L.W., Fields D. (2018). What Explains specialty coffee quality scores and prices: a case study from the cup of excellence program. J. Agric. Appl. Econ..

[88] Yang Y., Zhu L., Li J., Liu L., Su Y., Lei C., Xu M., Xu Q., Li X., Xu W. (2026). Plant-based fermented products: From traditional fermentation to controlled Bioprocessing—Mechanisms, functional effects and applications. Trends Food Sci. Technol..

