# Supplementary material for: Ultrasound-enhanced co-fermentation of Yunnan Arabica coffee with rose petals: Mass transfer, microbial succession, and molecular modeling
Source: Ultrason Sonochem. 2026 Oct 24;134:108127. doi: 10.1016/j.ultsonch.2026.108127 (PMC13734951; doi:10.1016/j.ultsonch.2026.108127)
Supplement: Supplementary file 1 [file mmc1.docx]

# **SUPPLEMENTARY INFORMATION**

# **1.Supplementary Table S1.**

Experimental calorimetric power calibration and Computational Fluid Dynamics (CFD) acoustic power dissipation validation across programmed acoustic power densities.

| **Programmed APD (W mL⁻¹)** | **Calorimetric (dT/dt)ₜ₌₀ (°C s⁻¹)** | **Calibrated Net Power Pcal (W)** | **Calorimetric APD Pcal (W mL⁻¹)** | **CFD Dissipation PCFD (W mL⁻¹)** | **Relative Deviation δ (%)** |
| --- | --- | --- | --- | --- | --- |
| 0.10 | 0.0234 ± 0.0007 | 49.0 ± 1.5 | 0.098 ± 0.003 | 0.104 | 6.12% |
| 0.30 | 0.0705 ± 0.0019 | 147.5 ± 4.0 | 0.295 ± 0.008 | 0.312 | 5.76% |
| 0.50 | 0.1176 ± 0.0026 | 246.0 ± 5.5 | 0.492 ± 0.011 | 0.524 | 6.50% |
| 0.65 | 0.1525 ± 0.0036 | 319.0 ± 7.5 | 0.638 ± 0.015 | 0.682 | 6.90% |
| 0.80 | 0.1852 ± 0.0043 | 387.5 ± 9.0 | 0.775 ± 0.018 | 0.831 | 7.23% |

**Notes and Abbreviations:**

1. Programmed APD: Nominated acoustic power density setting per unit volume (liquid working volume V = 500 mL, fluid mass M = 0.50 kg).

2. Calorimetric (dT/dt)ₜ₌₀: Initial volumetric temperature rise rate determined during the first 30 s of sonication, measured via a calibrated multi-junction thermocouple array (5 array points distributed radially at r/R = 0, 0.25, 0.5, 0.75, 0.95 and axially at z/H = 0.5) under rapid mechanical pre-recirculation (150 rpm) to eliminate local temperature gradient bias. Data represent Mean ± Standard Deviation (n = 3 independent calibration runs).

3. Calibrated Net Power Pcal: Delivered acoustic thermal power calculated via energy balance equation: Pcal = M · Cp · (dT/dt)ₜ₌₀, where Cp is the heat capacity of the coffee-rose multiphase mixture (approximated as 4.184 kJ kg⁻¹ °C⁻¹).

4. CFD Dissipation PCFD: Volume-integrated acoustic dissipation rate computed by integrating the acoustic loss tensor over the 3D fluid domain using the Helmholtz acoustic wave model in Computational Fluid Dynamics (ANSYS Fluent 2022 R2).

5. Relative Deviation δ (%): Calculated as δ = |Pcal - PCFD| / Pcal × 100%. The close agreement (relative deviation ranging between 5.76% and 7.23%) rigorously validates the predictive accuracy of the CFD acoustic field model against empirical calorimetric energy input.

# **2.Supplementary Figure S1.**

Computational Fluid Dynamics (CFD) numerical simulation of 3D acoustic pressure distribution, acoustic streaming velocity vector fields, and mesh independence analysis inside the continuous sonobioreactor.

**Detailed Methodological Notes and Computational Parameters:**

(a) Acoustic Pressure Distribution Slice: Simulated 2D r-z cross-sectional contour of acoustic pressure amplitude (pa in kPa) generated by the titanium probe operating at 20 kHz (input power density = 0.5 W mL⁻¹). The acoustic wave propagation was modeled using the linearized Helmholtz wave equation in ANSYS Fluent 2022 R2. Note the pronounced near-field acoustic attenuation and spatial intensity gradient extending from the horn tip into the bulk fluid, reflecting characteristic wave scattering.

(b) Acoustic Streaming & Flow Velocity Vectors: Vector field map illustrating time-averaged hydrodynamic recirculation patterns (m s⁻¹) induced by acoustic cavitation microjets and streaming momentum transfer. A downward high-velocity jet (peak velocity ~1.2 m s⁻¹) originates directly beneath the radiating face, driving twin toroidal circulation vortices that eliminate unsonicated dead zones across the vessel.

(c) Mesh Independence Analysis: Grid sensitivity evaluation performed across six polyhedral meshing levels (M1 to M6: 1.2 × 10⁵ to 2.2 × 10⁶ elements). Total volume-integrated acoustic dissipation power (PCFD, W) was tracked as the convergence criterion. The Grid Convergence Index (GCI) dropped below 0.5% at Mesh M4 (8.5 × 10⁵ elements), confirming that numerical discretization errors were negligible. Consequently, Mesh M4 was selected for all production-scale fluid dynamic simulations.

# **3.Supplementary Figure S2.**

**Caption：**

(a) Endpoint phenotype heatmap across fermentation treatments alongside calculated effect sizes (*ω*^2^).

(b, c) Remodeling magnitude of significant VOC features (*q*<0.05) and the macro-structural shift in chemical classes.

# **4.Supplementary Table S2.**

**S2A1. Free amino acid identities, molecular weights, and UHPLC calibration metrics**

Calibration curves were established using 25 authentic free-amino-acid standards combined with isotopically labeled internal standards (¹³C/¹⁵N-labeled) following online pre-column derivatization with OPA and FMOC-Cl (Section 2.3). LLOD, lower limit of detection; LLOQ, lower limit of quantification.

| **Analyte** | **CAS** | **MW (g mol⁻¹)** | **LLOD (nmol L⁻¹)** | **LLOQ (nmol L⁻¹)** | **Calibration R²** | **Internal Standard Calibration** | **Pre-column Derivatization** |
| --- | --- | --- | --- | --- | --- | --- | --- |
| 1-Methylhistidine | 332-80-9 | 169.18 | 39.06 | 78.12 | 0.9917 | Authentic Std + ¹³C/¹⁵N-IS | OPA / FMOC-Cl |
| 3-Methylhistidine | 368-16-1 | 169.18 | 29.30 | 58.59 | 0.9932 | Authentic Std + ¹³C/¹⁵N-IS | OPA / FMOC-Cl |
| 4-Aminobutyric acid | 56-12-2 | 103.12 | 4.88 | 9.77 | 0.9928 | Authentic Std + ¹³C/¹⁵N-IS | OPA / FMOC-Cl |
| 4-Hydroxyproline | 51-35-4 | 131.13 | 39.06 | 78.12 | 0.9902 | Authentic Std + ¹³C/¹⁵N-IS | OPA / FMOC-Cl |
| 5-Hydroxylysine | 1190-94-9 | 162.19 | 2.44 | 4.88 | 0.9917 | Authentic Std + ¹³C/¹⁵N-IS | OPA / FMOC-Cl |
| β-Alanine | 107-95-9 | 89.09 | 156.25 | 312.50 | 0.9947 | Authentic Std + ¹³C/¹⁵N-IS | OPA / FMOC-Cl |
| Glycine | 56-40-6 | 75.07 | 625.00 | 1250.00 | 0.9944 | Authentic Std + ¹³C/¹⁵N-IS | OPA / FMOC-Cl |
| L-Alanine | 56-41-7 | 89.09 | 9.77 | 19.53 | 0.9988 | Authentic Std + ¹³C/¹⁵N-IS | OPA / FMOC-Cl |
| L-Arginine | 74-79-3 | 174.20 | 78.12 | 156.25 | 0.9906 | Authentic Std + ¹³C/¹⁵N-IS | OPA / FMOC-Cl |
| L-Asparagine | 70-47-3 | 132.12 | 19.53 | 39.06 | 0.9965 | Authentic Std + ¹³C/¹⁵N-IS | OPA / FMOC-Cl |
| L-Aspartic acid | 56-84-8 | 133.10 | 312.50 | 625.00 | 0.9997 | Authentic Std + ¹³C/¹⁵N-IS | OPA / FMOC-Cl |
| L-Citrulline | 372-75-8 | 175.19 | 39.06 | 78.12 | 0.9938 | Authentic Std + ¹³C/¹⁵N-IS | OPA / FMOC-Cl |
| L-Glutamic acid | 56-86-0 | 147.13 | 312.50 | 625.00 | 0.9954 | Authentic Std + ¹³C/¹⁵N-IS | OPA / FMOC-Cl |
| L-Glutamine | 56-85-9 | 146.14 | 625.00 | 1250.00 | 0.9966 | Authentic Std + ¹³C/¹⁵N-IS | OPA / FMOC-Cl |
| L-Histidine | 71-00-1 | 155.15 | 312.50 | 625.00 | 0.9957 | Authentic Std + ¹³C/¹⁵N-IS | OPA / FMOC-Cl |
| L-Lysine | 56-87-1 | 146.19 | 312.50 | 625.00 | 0.9995 | Authentic Std + ¹³C/¹⁵N-IS | OPA / FMOC-Cl |
| L-Methionine | 63-68-3 | 149.21 | 39.06 | 78.12 | 0.9925 | Authentic Std + ¹³C/¹⁵N-IS | OPA / FMOC-Cl |
| L-Ornithine | 70-26-8 | 132.16 | 312.50 | 625.00 | 0.9915 | Authentic Std + ¹³C/¹⁵N-IS | OPA / FMOC-Cl |
| L-Phenylalanine | 63-91-2 | 165.19 | 156.25 | 312.50 | 0.9996 | Authentic Std + ¹³C/¹⁵N-IS | OPA / FMOC-Cl |
| L-Proline | 147-85-3 | 115.13 | 312.50 | 625.00 | 0.9900 | Authentic Std + ¹³C/¹⁵N-IS | OPA / FMOC-Cl |
| L-Threonine | 72-19-5 | 119.12 | 312.50 | 625.00 | 0.9991 | Authentic Std + ¹³C/¹⁵N-IS | OPA / FMOC-Cl |
| L-Tryptophan | 73-22-3 | 204.23 | 39.06 | 78.12 | 0.9998 | Authentic Std + ¹³C/¹⁵N-IS | OPA / FMOC-Cl |
| L-Tyrosine | 60-18-4 | 181.19 | 2.44 | 4.88 | 0.9952 | Authentic Std + ¹³C/¹⁵N-IS | OPA / FMOC-Cl |
| L-Valine | 72-18-4 | 117.15 | 625.00 | 1250.00 | 0.9999 | Authentic Std + ¹³C/¹⁵N-IS | OPA / FMOC-Cl |
| L-Serine | 56-45-1 | 105.09 | 312.50 | 625.00 | 0.9997 | Authentic Std + ¹³C/¹⁵N-IS | OPA / FMOC-Cl |

**S2A2. Targeted free amino acid mass concentrations across fermentation treatments (Unit: μg mg⁻¹ DW)**

Concentrations are normalized to exact dry sample mass (m_sample, 19.7–20.9 mg DW, moisture content 10–12% corrected) and expressed as μg mg⁻¹ dry weight (DW) (numerically equivalent to mg g⁻¹ DW, Section 2.3). Data represent Mean ± SD of three independent biological replicates (n = 3). ND, not detected (below LLOD).

| **Analyte** | **NF** | **YF** | **RF15** | **RF30** | **RF45** | **RF60** | **RF75** |
| --- | --- | --- | --- | --- | --- | --- | --- |
| 1-Methylhistidine | ND | ND | ND | ND | ND | ND | ND |
| 3-Methylhistidine | ND | ND | ND | ND | ND | ND | ND |
| 4-Aminobutyric acid | 0.43 ± 0.04 | 0.88 ± 0.11 | 0.48 ± 0.05 | 0.51 ± 0.04 | 0.49 ± 0.04 | 0.68 ± 0.07 | 0.53 ± 0.06 |
| 4-Hydroxyproline | ND | ND | ND | ND | ND | ND | ND |
| 5-Hydroxylysine | ND | ND | ND | ND | ND | ND | ND |
| β-Alanine | ND | ND | ND | ND | ND | 0.22 ± 0.06 | 0.16 ± 0.04 |
| Glycine | 0.56 ± 0.02 | 1.07 ± 0.16 | 0.70 ± 0.08 | 0.56 ± 0.09 | 0.57 ± 0.02 | 1.06 ± 0.12 | 0.81 ± 0.09 |
| L-Alanine | 0.55 ± 0.08 | 0.94 ± 0.13 | 0.62 ± 0.10 | 0.55 ± 0.05 | 0.56 ± 0.18 | 1.08 ± 0.09 | 0.82 ± 0.08 |
| L-Arginine | 1.38 ± 0.45 | 0.06 ± 0.03 | 0.09 ± 0.07 | 0.10 ± 0.04 | 0.60 ± 0.12 | 2.16 ± 0.24 | 1.43 ± 0.18 |
| L-Asparagine | 2.42 ± 0.48 | 0.35 ± 0.05 | 0.22 ± 0.14 | 0.46 ± 0.09 | 0.86 ± 0.15 | 1.96 ± 0.22 | 1.48 ± 0.19 |
| L-Aspartic acid | 3.18 ± 0.33 | 1.72 ± 0.29 | 1.33 ± 0.25 | 1.41 ± 0.23 | 1.98 ± 0.22 | 3.03 ± 0.35 | 2.45 ± 0.28 |
| L-Citrulline | ND | ND | ND | ND | ND | ND | ND |
| L-Glutamic acid | 1.38 ± 0.16 | 0.49 ± 0.03 | 0.56 ± 0.11 | 0.70 ± 0.05 | 2.41 ± 0.25 | 3.62 ± 0.42 | 2.62 ± 0.31 |
| L-Glutamine | ND | ND | ND | ND | ND | 0.23 ± 0.04 | 0.16 ± 0.03 |
| L-Histidine | 0.18 ± 0.01 | 0.25 ± 0.10 | 0.11 ± 0.02 | 0.09 ± 0.01 | 0.08 ± 0.02 | 0.12 ± 0.02 | 0.19 ± 0.03 |
| L-Lysine | 0.16 ± 0.02 | 0.13 ± 0.04 | 0.21 ± 0.09 | 0.19 ± 0.04 | 0.65 ± 0.08 | 1.08 ± 0.12 | 0.79 ± 0.09 |
| L-Methionine | ND | ND | ND | ND | ND | ND | ND |
| L-Ornithine | ND | ND | ND | ND | ND | 0.38 ± 0.05 | 0.24 ± 0.04 |
| L-Phenylalanine | 0.09 ± 0.05 | 0.26 ± 0.01 | 0.27 ± 0.01 | 0.24 ± 0.05 | 0.35 ± 0.04 | 0.37 ± 0.04 | 0.27 ± 0.03 |
| L-Proline | ND | ND | ND | ND | ND | ND | ND |
| L-Threonine | 0.08 ± 0.06 | 0.11 ± 0.10 | 0.04 ± 0.01 | 0.03 ± 0.02 | 0.19 ± 0.03 | 0.23 ± 0.03 | 0.17 ± 0.02 |
| L-Tryptophan | 0.06 ± 0.01 | 0.17 ± 0.03 | 0.13 ± 0.06 | 0.14 ± 0.01 | 0.14 ± 0.02 | 0.20 ± 0.02 | 0.15 ± 0.02 |
| L-Tyrosine | ND | ND | ND | ND | ND | ND | ND |
| L-Valine | 1.28 ± 0.04 | 0.92 ± 0.04 | 0.62 ± 0.08 | 0.67 ± 0.16 | 0.97 ± 0.11 | 1.08 ± 0.14 | 0.80 ± 0.10 |
| L-Serine | 0.33 ± 0.06 | ND | ND | ND | ND | 0.38 ± 0.05 | 0.29 ± 0.04 |
| **Total FAA Pool (μg mg⁻¹ DW)** | **12.08 ± 0.92** | **7.35 ± 0.58** | **5.38 ± 0.49** | **5.65 ± 0.46** | **9.85 ± 0.74** | **16.82 ± 1.25** | **12.36 ± 0.98** |

**S2A3. Targeted free amino acid molar concentrations across fermentation treatments (Unit: μmol g⁻¹ DW)**

Molar concentrations (μmol g⁻¹ DW, equivalent to mmol kg⁻¹ DW) were derived from mass concentrations (S2A2) divided by analyte molecular weights (S2A1). The total free amino acid (FAA) pool exhibits a biphasic depletion–accumulation trajectory (Fig. 3d), transitioning from initial microbial assimilation (NF: 94.74 μmol g⁻¹ DW to RF15–RF30: 47.13–47.89 μmol g⁻¹ DW) to pronounced proteolytic accumulation peaking at 125.43 ± 9.62 μmol g⁻¹ DW in RF60.

| **Analyte** | **NF** | **YF** | **RF15** | **RF30** | **RF45** | **RF60** | **RF75** |
| --- | --- | --- | --- | --- | --- | --- | --- |
| 1-Methylhistidine | ND | ND | ND | ND | ND | ND | ND |
| 3-Methylhistidine | ND | ND | ND | ND | ND | ND | ND |
| 4-Aminobutyric acid | 4.13 ± 0.38 | 8.50 ± 1.10 | 4.67 ± 0.49 | 4.97 ± 0.39 | 4.79 ± 0.35 | 6.60 ± 0.70 | 5.14 ± 0.58 |
| 4-Hydroxyproline | ND | ND | ND | ND | ND | ND | ND |
| 5-Hydroxylysine | ND | ND | ND | ND | ND | ND | ND |
| β-Alanine | ND | ND | ND | ND | ND | 2.43 ± 0.68 | 1.80 ± 0.45 |
| Glycine | 7.40 ± 0.27 | 14.20 ± 2.20 | 9.30 ± 1.00 | 7.40 ± 1.20 | 7.59 ± 0.27 | 14.10 ± 1.60 | 10.80 ± 1.20 |
| L-Alanine | 6.19 ± 0.93 | 10.50 ± 1.50 | 7.00 ± 1.10 | 6.12 ± 0.55 | 6.30 ± 2.00 | 12.10 ± 1.00 | 9.20 ± 0.90 |
| L-Arginine | 7.90 ± 2.60 | 0.35 ± 0.15 | 0.54 ± 0.43 | 0.60 ± 0.24 | 3.45 ± 0.68 | 12.40 ± 1.38 | 8.20 ± 1.05 |
| L-Asparagine | 18.30 ± 3.60 | 2.67 ± 0.41 | 1.70 ± 1.10 | 3.51 ± 0.67 | 6.50 ± 1.10 | 14.80 ± 1.65 | 11.20 ± 1.40 |
| L-Aspartic acid | 23.90 ± 2.50 | 12.90 ± 2.20 | 10.00 ± 1.90 | 10.60 ± 1.70 | 14.90 ± 1.70 | 22.80 ± 2.60 | 18.40 ± 2.10 |
| L-Citrulline | ND | ND | ND | ND | ND | ND | ND |
| L-Glutamic acid | 9.40 ± 1.10 | 3.35 ± 0.23 | 3.82 ± 0.77 | 4.74 ± 0.35 | 16.35 ± 1.66 | 24.60 ± 2.85 | 17.80 ± 2.10 |
| L-Glutamine | ND | ND | ND | ND | ND | 1.59 ± 0.26 | 1.10 ± 0.20 |
| L-Histidine | 1.19 ± 0.09 | 1.64 ± 0.62 | 0.72 ± 0.14 | 0.58 ± 0.07 | 0.50 ± 0.16 | 0.78 ± 0.15 | 1.22 ± 0.19 |
| L-Lysine | 1.07 ± 0.13 | 0.89 ± 0.27 | 1.45 ± 0.62 | 1.27 ± 0.30 | 4.45 ± 0.55 | 7.40 ± 0.82 | 5.40 ± 0.62 |
| L-Methionine | ND | ND | ND | ND | ND | ND | ND |
| L-Ornithine | ND | ND | ND | ND | ND | 2.88 ± 0.38 | 1.82 ± 0.30 |
| L-Phenylalanine | 0.56 ± 0.32 | 1.58 ± 0.04 | 1.63 ± 0.08 | 1.48 ± 0.30 | 2.14 ± 0.25 | 2.25 ± 0.24 | 1.64 ± 0.18 |
| L-Proline | ND | ND | ND | ND | ND | ND | ND |
| L-Threonine | 0.63 ± 0.48 | 0.92 ± 0.87 | 0.35 ± 0.12 | 0.25 ± 0.20 | 1.58 ± 0.22 | 1.92 ± 0.25 | 1.42 ± 0.17 |
| L-Tryptophan | 0.32 ± 0.05 | 0.85 ± 0.14 | 0.64 ± 0.30 | 0.67 ± 0.05 | 0.70 ± 0.11 | 0.98 ± 0.11 | 0.73 ± 0.09 |
| L-Tyrosine | ND | ND | ND | ND | ND | ND | ND |
| L-Valine | 10.93 ± 0.33 | 7.81 ± 0.34 | 5.31 ± 0.72 | 5.70 ± 1.40 | 8.25 ± 0.96 | 9.20 ± 1.20 | 6.80 ± 0.85 |
| L-Serine | 3.12 ± 0.59 | ND | ND | ND | ND | 3.60 ± 0.48 | 2.76 ± 0.38 |
| **Total FAA Pool (μmol g⁻¹ DW)** | **94.74 ± 6.85** | **65.31 ± 4.92** | **47.13 ± 3.84** | **47.89 ± 3.65** | **76.80 ± 5.42** | **125.43 ± 9.62** | **92.63 ± 7.35** |

**S2B1. Key volatile organic compound (VOC) identifications and olfactory descriptors via GC-MS/O**

Volatile compounds were extracted via HS-SPME (50/30 μm DVB/CAR/PDMS) and analyzed on a Trace 1610–TSQ 9610 GC-MS equipped with a Sniffer 9100 olfactory detection port and a polar DB-WAX capillary column (Section 2.3). Compound identities were assigned by integrating retention indices (RI, determined using C8–C40 n-alkanes), matching mass spectra against the NIST 2020 library, and comparing olfactory descriptors from four trained assessors.

| **Peak ID** | **Compound Name** | **CAS** | **RT (min)** | **RI (DB-WAX)** | **Olfactory Descriptors (GC-O & Database)** | **Identification Basis** |
| --- | --- | --- | --- | --- | --- | --- |
| 84 | Ethyl 3-methylbutanoate | 108-64-5 | 6.700 | 1069.4 | Fruity, apple, pineapple, sweet | RI + NIST20 + GC-O |
| 150 | β-Myrcene | 123-35-3 | 8.322 | 1164.4 | Floral, balsamic, rose-like, herbaceous, terpene | RI + NIST20 + GC-O |
| 209 | Ethyl hexanoate | 123-66-0 | 9.490 | 1236.1 | Fruity, pineapple, apple peel, sweet, waxy | RI + NIST20 + GC-O |
| 236 | 1-Heptanol | 111-70-6 | 9.938 | 1264.7 | Leafy, green, floral, peony, strawberry | RI + NIST20 + GC-O |
| 243 | Methylpyrazine | 109-08-0 | 10.062 | 1272.6 | Roasted, nutty, cocoa, chocolate, peanut | RI + NIST20 + GC-O |
| 305 | 2,5-Dimethylpyrazine | 123-32-0 | 10.929 | 1330.7 | Roasted nuts, cocoa, roast beef, earthy, bread | RI + NIST20 + GC-O |
| 334 | 1-Hexanol | 111-27-3 | 11.296 | 1356.5 | Fruity, green, floral, freshly cut grass, sweet alcohol | RI + NIST20 + GC-O |
| 384 | 2-Ethyl-5-methylpyrazine | 13360-64-0 | 11.931 | 1401.3 | Nutty, roasted, coffee, sweet, fruity | RI + NIST20 + GC-O |
| 465 | Furfural | 98-01-1 | 12.824 | 1470.4 | Sweet, baked bread, almond, woody, caramel | RI + NIST20 + GC-O |
| 469 | 2-Ethyl-3,5-dimethylpyrazine | 13925-07-0 | 12.884 | 1475.0 | Roasted nuts, coffee, earthy, burnt almonds, cocoa | RI + NIST20 + GC-O |
| 543 | Benzaldehyde | 100-52-7 | 13.675 | 1536.6 | Almond, cherry, sweet, burnt sugar | RI + NIST20 + GC-O |
| 558 | Linalool | 78-70-6 | 13.844 | 1549.7 | Floral, rose, citrus, bergamot, lavender, sweet | RI + NIST20 + GC-O |
| 685 | Phenylacetaldehyde | 122-78-1 | 15.154 | 1656.1 | Floral, honey, hyacinth, rose, sweet, clover | RI + NIST20 + GC-O |
| 733 | 2-Acetyl-3-methylpyrazine | 23787-80-6 | 15.645 | 1697.5 | Roasted, toasted, nutty, grain, vegetable | RI + NIST20 + GC-O |
| 910 | Guaiacol (2-Methoxyphenol) | 90-05-1 | 17.556 | 1872.5 | Smoky, phenolic, medicinal, woody, burnt | RI + NIST20 + GC-O |
| 930 | Benzyl alcohol | 100-51-6 | 17.738 | 1889.9 | Floral, rose, berry, cherry, balsamic, sweet | RI + NIST20 + GC-O |
| 1073 | 4-Ethylguaiacol | 2785-89-9 | 19.301 | 2043.3 | Smoky, clove, phenolic, medicinal, bacon | RI + NIST20 + GC-O |
| 1191 | Eugenol | 97-53-0 | 20.617 | 2172.0 | Clove, floral, rose-carnation, honey, warm spicy | RI + NIST20 + GC-O |
| 1194 | 4-Ethylphenol | 123-07-9 | 20.649 | 2175.1 | Phenolic, smoky, woody, leather, medicinal | RI + NIST20 + GC-O |
| 1212 | 4-Vinylguaiacol | 7786-61-0 | 20.880 | 2198.0 | Spicy, clove, smoky, curry, phenolic | RI + NIST20 + GC-O |
| 1396 | Indole | 120-72-9 | 23.263 | 2437.1 | Earthy, animalic, mothball, pungent at high levels | RI + NIST20 + GC-O |
| 1420 | 3-Methylindole (Skatole) | 83-34-1 | 23.762 | 2487.1 | Earthy, pungent, fecal, mothball off-odor | RI + NIST20 + GC-O |

**S2B2. Integrated peak areas of key volatile flavor compounds across fermentation treatments (Unit: 10⁶ arbitrary peak-area units)**

Values represent Mean ± SD (n = 3 independent batches, detected in 3/3 replicates) expressed as 10⁶ integrated peak-area units normalized to internal standard (2-octanol). Characteristic floral-fruity markers (β-myrcene ID 150, 1-hexanol ID 334, linalool ID 558, benzyl alcohol ID 930, eugenol ID 1191) were substantially enriched in RF treatments, whereas pungent/earthy off-odorants (guaiacol ID 910, 4-ethylguaiacol ID 1073, indole ID 1396, 3-methylindole ID 1420) were markedly suppressed.

| **ID** | **Compound** | **NF** | **YF** | **RF15** | **RF30** | **RF45** | **RF60** | **RF75** |
| --- | --- | --- | --- | --- | --- | --- | --- | --- |
| 84 | Ethyl 3-methylbutanoate | 12.69 ± 0.20 | 63.2 ± 3.8 | 51.77 ± 0.76 | 61.9 ± 6.3 | 95.1 ± 6.0 | 105.3 ± 5.0 | 72.8 ± 6.6 |
| 150 | β-Myrcene | 87.8 ± 2.6 | 55.3 ± 9.5 | 234.9 ± 3.1 | 317 ± 39 | 374 ± 25 | 499 ± 40 | 403 ± 82 |
| 209 | Ethyl hexanoate | 60.7 ± 2.1 | 106.9 ± 6.9 | 123 ± 12 | 147 ± 16 | 168 ± 27 | 200 ± 14 | 179 ± 20 |
| 236 | 1-Heptanol | 24.58 ± 0.60 | 134 ± 15 | 190.4 ± 1.7 | 173.6 ± 8.8 | 183 ± 14 | 154.8 ± 9.2 | 202 ± 17 |
| 243 | Methylpyrazine | 5175 ± 244 | 7482 ± 136 | 4796 ± 159 | 3937 ± 179 | 3924 ± 255 | 4396 ± 369 | 4930 ± 270 |
| 305 | 2,5-Dimethylpyrazine | 5241 ± 169 | 6213 ± 95 | 4186 ± 88 | 3672 ± 140 | 3729 ± 183 | 3820 ± 288 | 4543 ± 231 |
| 334 | 1-Hexanol | 122.7 ± 2.4 | 64.4 ± 4.0 | 101.3 ± 1.8 | 165 ± 10 | 297 ± 12 | 427 ± 29 | 282 ± 17 |
| 384 | 2-Ethyl-5-methylpyrazine | 2616 ± 92 | 3409 ± 74 | 2179 ± 44 | 2090 ± 85 | 2128 ± 94 | 2130 ± 163 | 2392 ± 93 |
| 465 | Furfural | 4134 ± 164 | 4512 ± 64 | 5100 ± 93 | 5389 ± 173 | 5540 ± 175 | 5872 ± 328 | 5167 ± 213 |
| 469 | 2-Ethyl-3,5-dimethylpyrazine | 1562 ± 45 | 4252 ± 265 | 2882 ± 27 | 2443 ± 144 | 2211 ± 98 | 2315 ± 189 | 3193 ± 167 |
| 543 | Benzaldehyde | 916 ± 18 | 1586 ± 37 | 1416 ± 23 | 1283 ± 41 | 1200 ± 70 | 1259 ± 131 | 1190 ± 60 |
| 558 | Linalool | 135.8 ± 2.8 | 361 ± 58 | 345.1 ± 6.5 | 415 ± 49 | 583 ± 33 | 647 ± 81 | 711 ± 98 |
| 685 | Phenylacetaldehyde | 985 ± 22 | 2104 ± 158 | 2487 ± 38 | 2513 ± 130 | 2414 ± 103 | 2366 ± 80 | 2179 ± 134 |
| 733 | 2-Acetyl-3-methylpyrazine | 58.9 ± 1.7 | 39.3 ± 6.7 | 37.73 ± 0.44 | 26.5 ± 3.6 | 14.2 ± 1.1 | 15.2 ± 2.0 | 8.4 ± 1.4 |
| 910 | Guaiacol (2-Methoxyphenol) | 1426 ± 63 | 2104 ± 116 | 1427 ± 24 | 1136 ± 102 | 780 ± 57 | 750 ± 76 | 541 ± 33 |
| 930 | Benzyl alcohol | 148.8 ± 5.9 | 175 ± 17 | 306.9 ± 2.9 | 430 ± 38 | 553 ± 40 | 612 ± 64 | 665 ± 49 |
| 1073 | 4-Ethylguaiacol | 1685 ± 36 | 2328 ± 162 | 1863 ± 25 | 1282 ± 123 | 874 ± 49 | 864 ± 80 | 619 ± 49 |
| 1191 | Eugenol | 33.5 ± 1.3 | 10.01 ± 0.28 | 25.68 ± 0.65 | 34.1 ± 3.2 | 67.0 ± 3.9 | 93.8 ± 7.5 | 97.6 ± 8.0 |
| 1194 | 4-Ethylphenol | 287.9 ± 7.8 | 489 ± 24 | 1198 ± 24 | 1067 ± 92 | 1044 ± 59 | 1112 ± 97 | 967 ± 69 |
| 1212 | 4-Vinylguaiacol | 2455 ± 150 | 3774 ± 232 | 2628 ± 110 | 2478 ± 294 | 2648 ± 143 | 2513 ± 175 | 2877 ± 239 |
| 1396 | Indole | 436 ± 14 | 1524 ± 71 | 1120 ± 43 | 864 ± 103 | 741 ± 62 | 769 ± 59 | 807 ± 64 |
| 1420 | 3-Methylindole (Skatole) | 104.5 ± 2.1 | 201.7 ± 7.1 | 189.7 ± 4.3 | 155 ± 12 | 134.1 ± 8.1 | 136.2 ± 6.8 | 111.2 ± 5.6 |

# **5.Supplementary Figure S3.**

**Caption:**

(a) Standardized z-score heatmap of the 1,297 core differential features across 21 independent batches (formerly Fig. 4g).
(b) Global treatment effect-size (Ω^2^ ) distribution against statistical significance (formerly Fig. 4d).
(c) Pre-specified differential contrasts across treatment pairs (formerly Fig. 4e).

(d) UpSet intersection plot of significant metabolic features (*q*<0.05) across comparison groups (formerly Fig. 4f).

# **6.Supplementary Figure S4.**

**Caption:**

(a) Global pathway response heatmap across pre-specified treatment contrasts (formerly Fig. 5b).
(b) Normalized median pathway activity score matrix across NF, YF, and RF15–RF75 groups (formerly Fig. 5c).

(c) Impact magnitude ranking of leading metabolic drivers evaluated by log_2_ fold change (RF mean /YF) (formerly Fig. 5e).

# **7.Supplementary Figure S5.**

**Caption:**

(a) Repeated-container longitudinal sampling design across 3 independent containers and 4 time points (0, 24, 48, and 72 h), yielding 81 sequenced libraries and 1,493 rarefied ASVs.
(b) Library-level sequencing quality summary (mean ± SD), including raw and clean read depths, Q30 base-call quality ratio, and Good’s coverage.
(c) Two-way ANOVA effect sizes (partial *η*^2^) for richness and evenness/diversity indices attributable to treatment, time, and their interaction.
(d) Replicate-level abundance validation of six core bacterial genera at the fermentation endpoint (bars indicate mean relative abundance ± SD; open circles denote individual container observations, *n*=3).
(e) Sensitivity analysis of prespecified PERMANOVA models (*R*^2^) comparing community variance explanation before and after excluding cyanobacterial/chloroplast sequences.

# **8.Supplementary Figure S6.**

**Caption:**

(a) Phylum-level relative abundance across treatments at the 72 h endpoint, highlighting the dominance of Firmicutes (Bacillota) in RF groups.
(b) Standardized Z-score heatmap of endpoint genus signatures (P1–P12) across treatment groups.
(c) Prevalence–abundance distribution map distinguishing core and non-core bacterial genera (abundance shown on a logarithmic scale).

(d–f) Dose-response bar plots of *α*-diversity metrics across the RF gradient (RF15–RF75) at 72 h: observed ASV richness (d), Shannon diversity index (e), and Pielou evenness (f) (mean ± SD, open circles denote replicate observations, *n*=3).

(f) PERMANOVA variance explanation (*R*^2^ ) for prespecified linear (*q*=0.0006) and quadratic (*q*=0.003) models of ordered community response.

(h) Estimated centered log-ratio (CLR, log_2_ ) dose contrasts with 95% confidence intervals for key responsive genera. (i) Evidence-linked schematic summarizing treatment-specific succession (*R*^2^ =0.583), LAB-dominated RF endpoints (72.8%–80.1%), and ordered nonlinear dose structure.

# **9.Supplementary Figure S7.**

**Fungal ITS1–ITS2 amplicon community profiling, alpha-diversity trajectories.**

**Caption:**

(a) Phylum-level relative abundance (%) across 81 sequenced libraries rarefied to 3,500 reads per sample. The community is partitioned primarily between *p__Ascomycota* and *p__unclassified*_Fungi.

(b) Genus-level taxonomic restructuring showing the high relative abundance of *g__Saccharomyces* in inoculated groups (reaching 90.4–96.1% in RF15–RF30 and 60.7–88.2% in RF45–RF75 at 72 h, alongside *g__unclassified*_Fungi), contrasting with uninoculated NF (Con_R) where *g__Saccharomyces* remained at 17.8–27.4% with co-occurring *g__Pichia* (4.3–9.6%) at 72 h.

(c) Longitudinal trajectories of fungal ITS Shannon diversity (base 2, mean ± SD, n = 3). Uninoculated NF exhibited a progressive rise in fungal diversity by 48–72 h, whereas inoculated YF and RF groups maintained lower diversity.

# **10.Supplementary Table S3.**

**Sequencing quality control statistics for bacterial 16S rRNA and fungal ITS amplicon libraries**

Sample nomenclature correspondence: NF = Con_R; YF = Con_GR; RF15–RF75 = FRC_G15R–FRC_G75R. The final integer (1, 2, or 3) denotes independent biological container replicates across 0, 24, 48, and 72 h.

**S3A. Bacterial 16S rRNA (V3–V4) sequencing quality control metrics (81 samples)**

| **Sample ID** | **Raw reads** | **Clean reads** | **Q20 (%)** | **Q30 (%)** | **DADA2 ASV reads** | **ASVs observed** | **Coverage (DADA2)** | **Coverage (Supplier)** |
| --- | --- | --- | --- | --- | --- | --- | --- | --- |
| Con_R_0h_1 | 66,759 | 60,705 | 97.64 | 92.50 | 59,454 | 32 | 0.999966 | 0.999958 |
| Con_R_0h_2 | 60,261 | 54,760 | 97.64 | 92.65 | 53,664 | 22 | 1.000000 | 0.999980 |
| Con_R_0h_3 | 64,235 | 58,663 | 97.79 | 92.93 | 57,384 | 25 | 1.000000 | 0.999924 |
| Con_R_24h_1 | 76,651 | 70,535 | 97.89 | 93.18 | 67,639 | 94 | 0.999882 | 0.999792 |
| Con_R_24h_2 | 72,149 | 66,434 | 97.80 | 92.92 | 63,764 | 93 | 0.999827 | 0.999813 |
| Con_R_24h_3 | 105,287 | 96,893 | 97.97 | 93.38 | 92,464 | 139 | 0.999773 | 0.999647 |
| Con_R_48h_1 | 58,171 | 53,299 | 97.68 | 92.62 | 49,671 | 126 | 0.999738 | 0.999896 |
| Con_R_48h_2 | 58,128 | 54,236 | 97.94 | 93.26 | 50,498 | 103 | 0.999861 | 0.999875 |
| Con_R_48h_3 | 57,943 | 53,679 | 98.04 | 93.52 | 49,106 | 134 | 0.999776 | 0.999792 |
| Con_R_72h_1 | 57,972 | 53,621 | 97.91 | 93.18 | 48,960 | 107 | 0.999816 | 0.999750 |
| Con_R_72h_2 | 57,703 | 53,518 | 97.89 | 93.17 | 48,608 | 107 | 0.999753 | 0.999896 |
| Con_R_72h_3 | 57,625 | 52,905 | 97.91 | 93.18 | 48,148 | 94 | 0.999834 | 0.999792 |
| Con_GR_24h_1 | 58,772 | 54,052 | 98.04 | 93.56 | 50,402 | 38 | 0.999901 | 0.999958 |
| Con_GR_24h_2 | 76,450 | 69,848 | 97.81 | 93.05 | 64,026 | 66 | 0.999844 | 0.999896 |
| Con_GR_24h_3 | 61,786 | 56,239 | 97.98 | 93.44 | 52,088 | 50 | 0.999923 | 0.999938 |
| Con_GR_48h_1 | 107,226 | 98,528 | 97.70 | 92.64 | 93,804 | 119 | 0.999851 | 0.999667 |
| Con_GR_48h_2 | 61,445 | 56,890 | 98.02 | 93.47 | 54,777 | 77 | 0.999890 | 0.999875 |
| Con_GR_48h_3 | 74,918 | 68,948 | 97.79 | 92.86 | 65,893 | 86 | 0.999788 | 0.999750 |
| Con_GR_72h_1 | 64,740 | 59,840 | 97.64 | 92.54 | 53,878 | 84 | 0.999833 | 0.999875 |
| Con_GR_72h_2 | 58,575 | 54,116 | 97.96 | 93.29 | 48,097 | 62 | 0.999938 | 0.999958 |
| Con_GR_72h_3 | 58,668 | 54,150 | 97.74 | 92.75 | 49,920 | 106 | 0.999780 | 0.999938 |
| FRC_G15R_0h_1 | 76,704 | 69,865 | 97.91 | 93.20 | 68,625 | 38 | 0.999942 | 0.999917 |
| FRC_G15R_0h_2 | 71,970 | 65,498 | 97.68 | 92.65 | 64,227 | 27 | 1.000000 | 1.000000 |
| FRC_G15R_0h_3 | 87,121 | 78,081 | 95.95 | 88.10 | 76,966 | 33 | 1.000000 | 0.999942 |
| FRC_G15R_24h_1 | 59,313 | 54,130 | 97.95 | 93.39 | 50,231 | 54 | 0.999821 | 0.999938 |
| FRC_G15R_24h_2 | 57,898 | 52,773 | 98.11 | 93.75 | 48,346 | 71 | 0.999793 | 0.999917 |
| FRC_G15R_24h_3 | 62,340 | 57,057 | 98.21 | 94.05 | 52,434 | 59 | 0.999847 | 0.999854 |
| FRC_G15R_48h_1 | 72,341 | 66,689 | 97.86 | 93.08 | 61,762 | 39 | 0.999935 | 0.999958 |
| FRC_G15R_48h_2 | 57,694 | 53,243 | 98.01 | 93.42 | 49,220 | 28 | 0.999919 | 0.999875 |
| FRC_G15R_48h_3 | 57,423 | 52,992 | 98.03 | 93.46 | 48,919 | 38 | 0.999857 | 0.999958 |
| FRC_G15R_72h_1 | 80,255 | 73,925 | 98.11 | 93.70 | 66,411 | 42 | 0.999925 | 0.999834 |
| FRC_G15R_72h_2 | 60,242 | 55,762 | 98.08 | 93.57 | 49,878 | 37 | 0.999960 | 0.999938 |
| FRC_G15R_72h_3 | 62,160 | 57,409 | 97.98 | 93.27 | 51,787 | 36 | 0.999981 | 0.999917 |
| FRC_G30R_0h_1 | 74,629 | 68,322 | 97.72 | 92.74 | 66,256 | 61 | 0.999925 | 0.999917 |
| FRC_G30R_0h_2 | 97,230 | 88,885 | 97.79 | 92.90 | 85,852 | 97 | 0.999930 | 0.999709 |
| FRC_G30R_0h_3 | 79,279 | 73,049 | 97.76 | 92.80 | 71,160 | 68 | 0.999972 | 0.999854 |
| FRC_G30R_24h_1 | 66,793 | 60,916 | 97.72 | 92.77 | 57,296 | 56 | 0.999913 | 0.999896 |
| FRC_G30R_24h_2 | 57,711 | 52,945 | 98.13 | 93.82 | 48,969 | 35 | 0.999898 | 0.999917 |
| FRC_G30R_24h_3 | 59,910 | 54,668 | 97.92 | 93.23 | 50,599 | 50 | 0.999901 | 0.999834 |
| FRC_G30R_48h_1 | 58,265 | 53,411 | 97.95 | 93.28 | 48,808 | 24 | 0.999959 | 0.999979 |
| FRC_G30R_48h_2 | 59,182 | 54,632 | 98.03 | 93.44 | 50,490 | 26 | 0.999980 | 0.999960 |
| FRC_G30R_48h_3 | 59,516 | 54,406 | 97.90 | 93.11 | 49,149 | 35 | 0.999959 | 0.999875 |
| FRC_G30R_72h_1 | 74,412 | 68,635 | 98.13 | 93.71 | 60,477 | 49 | 0.999901 | 0.999771 |
| FRC_G30R_72h_2 | 66,888 | 62,097 | 98.18 | 93.83 | 55,124 | 45 | 0.999946 | 0.999917 |
| FRC_G30R_72h_3 | 78,402 | 72,204 | 97.99 | 93.40 | 61,144 | 74 | 0.999820 | 0.999834 |
| FRC_G45R_0h_1 | 61,084 | 55,695 | 97.61 | 92.50 | 55,091 | 55 | 0.999837 | 0.999896 |
| FRC_G45R_0h_2 | 81,697 | 74,632 | 97.62 | 92.61 | 72,982 | 86 | 0.999822 | 0.999854 |
| FRC_G45R_0h_3 | 61,114 | 55,936 | 97.81 | 92.92 | 55,254 | 41 | 1.000000 | 0.999917 |
| FRC_G45R_24h_1 | 101,354 | 92,406 | 97.98 | 93.43 | 84,525 | 57 | 0.999929 | 0.999834 |
| FRC_G45R_24h_2 | 79,419 | 72,800 | 98.02 | 93.46 | 65,994 | 46 | 0.999924 | 1.000000 |
| FRC_G45R_24h_3 | 80,436 | 73,782 | 98.12 | 93.77 | 67,345 | 50 | 0.999896 | 0.999854 |
| FRC_G45R_48h_1 | 58,965 | 53,924 | 97.82 | 92.95 | 50,610 | 33 | 0.999980 | 0.999931 |
| FRC_G45R_48h_2 | 59,489 | 54,336 | 98.03 | 93.46 | 50,554 | 37 | 0.999921 | 0.999938 |
| FRC_G45R_48h_3 | 60,978 | 55,474 | 96.47 | 89.15 | 52,620 | 26 | 0.999981 | 0.999979 |
| FRC_G45R_72h_1 | 63,498 | 58,617 | 98.27 | 94.11 | 51,788 | 65 | 0.999884 | 0.999834 |
| FRC_G45R_72h_2 | 68,765 | 63,352 | 98.09 | 93.59 | 56,570 | 75 | 0.999841 | 0.999875 |
| FRC_G45R_72h_3 | 66,272 | 61,149 | 98.00 | 93.41 | 54,668 | 83 | 0.999799 | 0.999875 |
| FRC_G60R_0h_1 | 65,876 | 60,031 | 97.72 | 92.72 | 59,096 | 50 | 0.999898 | 0.999917 |
| FRC_G60R_0h_2 | 61,668 | 56,114 | 97.70 | 92.73 | 55,462 | 55 | 0.999946 | 0.999917 |
| FRC_G60R_0h_3 | 60,406 | 55,304 | 97.63 | 92.65 | 54,509 | 47 | 0.999945 | 0.999875 |
| FRC_G60R_24h_1 | 59,163 | 54,042 | 98.00 | 93.43 | 50,100 | 154 | 0.999681 | 0.999792 |
| FRC_G60R_24h_2 | 58,308 | 53,290 | 98.05 | 93.56 | 49,640 | 141 | 0.999678 | 0.999854 |
| FRC_G60R_24h_3 | 60,122 | 55,184 | 97.95 | 93.33 | 50,750 | 148 | 0.999764 | 0.999771 |
| FRC_G60R_48h_1 | 79,002 | 73,203 | 97.95 | 93.16 | 67,621 | 88 | 0.999823 | 0.999854 |
| FRC_G60R_48h_2 | 58,502 | 53,742 | 97.91 | 93.06 | 49,069 | 66 | 0.999878 | 0.999917 |
| FRC_G60R_48h_3 | 72,032 | 66,348 | 98.09 | 93.62 | 60,657 | 87 | 0.999868 | 0.999750 |
| FRC_G60R_72h_1 | 75,182 | 69,252 | 97.85 | 92.93 | 62,904 | 60 | 0.999889 | 0.999772 |
| FRC_G60R_72h_2 | 70,504 | 65,227 | 98.20 | 93.93 | 58,587 | 61 | 0.999932 | 0.999917 |
| FRC_G60R_72h_3 | 68,609 | 63,363 | 98.00 | 93.31 | 57,644 | 59 | 0.999861 | 0.999854 |
| FRC_G75R_0h_1 | 64,191 | 58,851 | 97.55 | 92.36 | 55,957 | 101 | 0.999803 | 0.999792 |
| FRC_G75R_0h_2 | 61,747 | 56,580 | 97.88 | 93.11 | 53,159 | 105 | 0.999737 | 0.999917 |
| FRC_G75R_0h_3 | 61,366 | 56,213 | 97.67 | 92.59 | 53,771 | 111 | 0.999870 | 0.999813 |
| FRC_G75R_24h_1 | 58,971 | 54,427 | 97.76 | 92.85 | 52,807 | 91 | 0.999792 | 0.999875 |
| FRC_G75R_24h_2 | 58,797 | 54,056 | 97.98 | 93.36 | 51,285 | 89 | 0.999708 | 0.999834 |
| FRC_G75R_24h_3 | 59,089 | 53,939 | 97.85 | 93.00 | 51,698 | 78 | 0.999845 | 0.999854 |
| FRC_G75R_48h_1 | 67,679 | 62,345 | 97.91 | 93.18 | 55,464 | 80 | 0.999820 | 0.999896 |
| FRC_G75R_48h_2 | 58,827 | 54,215 | 97.97 | 93.27 | 49,290 | 51 | 0.999939 | 0.999917 |
| FRC_G75R_48h_3 | 59,099 | 54,431 | 98.07 | 93.54 | 49,466 | 54 | 0.999899 | 0.999875 |
| FRC_G75R_72h_1 | 87,302 | 80,691 | 97.99 | 93.38 | 74,420 | 91 | 0.999852 | 0.999771 |
| FRC_G75R_72h_2 | 83,024 | 76,681 | 98.16 | 93.72 | 69,911 | 85 | 0.999814 | 0.999813 |
| FRC_G75R_72h_3 | 74,082 | 68,362 | 98.02 | 93.42 | 62,264 | 83 | 0.999855 | 0.999730 |

**S3B. Fungal ITS (ITS1–ITS2) sequencing quality control metrics (81 samples)**

| **Sample ID** | **Raw reads** | **Clean reads** | **Q20 (%)** | **Q30 (%)** | **DADA2 ASV reads** | **ASVs observed** | **Coverage (DADA2)** | **Coverage (Supplier)** |
| --- | --- | --- | --- | --- | --- | --- | --- | --- |
| Con_R_0h_1 | 71,957 | 21,298 | 98.25 | 94.43 | 3,500 | 12 | 1.000000 | 0.999774 |
| Con_R_0h_2 | 64,434 | 18,311 | 98.43 | 95.19 | 8,485 | 15 | 1.000000 | 0.999714 |
| Con_R_0h_3 | 70,948 | 20,250 | 98.24 | 94.32 | 9,412 | 14 | 1.000000 | 0.999735 |
| Con_R_24h_1 | 99,410 | 72,335 | 98.11 | 93.98 | 49,725 | 17 | 1.000000 | 0.999503 |
| Con_R_24h_2 | 108,499 | 78,328 | 98.11 | 94.10 | 53,381 | 16 | 1.000000 | 0.999429 |
| Con_R_24h_3 | 62,402 | 44,366 | 98.32 | 94.73 | 24,815 | 11 | 1.000000 | 0.999171 |
| Con_R_48h_1 | 100,770 | 62,236 | 98.23 | 94.44 | 34,607 | 66 | 1.000000 | 0.994857 |
| Con_R_48h_2 | 95,939 | 58,715 | 98.07 | 93.84 | 32,934 | 51 | 1.000000 | 0.996981 |
| Con_R_48h_3 | 104,880 | 64,091 | 98.30 | 94.77 | 35,153 | 53 | 1.000000 | 0.997429 |
| Con_R_72h_1 | 111,187 | 73,211 | 98.25 | 94.68 | 58,843 | 67 | 1.000000 | 0.995143 |
| Con_R_72h_2 | 122,091 | 77,857 | 98.26 | 94.70 | 61,577 | 49 | 1.000000 | 0.999143 |
| Con_R_72h_3 | 117,675 | 80,445 | 98.21 | 94.61 | 59,003 | 78 | 1.000000 | 0.996286 |
| Con_GR_24h_1 | 72,180 | 50,851 | 98.19 | 94.28 | 39,684 | 8 | 1.000000 | 1.000000 |
| Con_GR_24h_2 | 72,473 | 50,012 | 98.21 | 94.43 | 38,998 | 11 | 1.000000 | 0.999745 |
| Con_GR_24h_3 | 73,477 | 51,853 | 98.20 | 94.36 | 41,113 | 13 | 1.000000 | 0.999448 |
| Con_GR_48h_1 | 116,634 | 82,238 | 98.45 | 94.99 | 68,010 | 25 | 1.000000 | 0.998926 |
| Con_GR_48h_2 | 84,608 | 58,171 | 98.52 | 95.07 | 47,593 | 13 | 1.000000 | 0.999461 |
| Con_GR_48h_3 | 117,084 | 82,656 | 98.52 | 95.04 | 69,064 | 20 | 1.000000 | 1.000000 |
| Con_GR_72h_1 | 110,495 | 87,624 | 98.40 | 94.88 | 70,960 | 19 | 1.000000 | 0.999379 |
| Con_GR_72h_2 | 107,011 | 86,169 | 98.32 | 94.53 | 77,044 | 23 | 1.000000 | 1.000000 |
| Con_GR_72h_3 | 108,753 | 84,774 | 98.34 | 94.56 | 61,063 | 20 | 1.000000 | 0.999554 |
| FRC_G15R_0h_1 | 58,701 | 41,950 | 98.03 | 93.77 | 31,205 | 9 | 1.000000 | 0.999724 |
| FRC_G15R_0h_2 | 55,196 | 38,605 | 98.09 | 94.03 | 23,416 | 7 | 1.000000 | 1.000000 |
| FRC_G15R_0h_3 | 69,448 | 47,698 | 98.17 | 94.46 | 35,517 | 8 | 1.000000 | 1.000000 |
| FRC_G15R_24h_1 | 68,646 | 47,891 | 98.20 | 94.41 | 35,366 | 9 | 1.000000 | 1.000000 |
| FRC_G15R_24h_2 | 53,217 | 38,758 | 98.18 | 94.34 | 25,599 | 7 | 1.000000 | 1.000000 |
| FRC_G15R_24h_3 | 68,355 | 47,582 | 98.25 | 94.57 | 39,647 | 12 | 1.000000 | 0.999510 |
| FRC_G15R_48h_1 | 112,513 | 78,001 | 98.29 | 94.65 | 52,352 | 18 | 1.000000 | 1.000000 |
| FRC_G15R_48h_2 | 121,348 | 84,515 | 98.23 | 94.45 | 62,188 | 24 | 0.999984 | 0.999613 |
| FRC_G15R_48h_3 | 106,916 | 75,199 | 98.19 | 94.29 | 56,426 | 18 | 1.000000 | 1.000000 |
| FRC_G15R_72h_1 | 107,175 | 91,264 | 98.45 | 94.94 | 76,385 | 68 | 1.000000 | 0.996857 |
| FRC_G15R_72h_2 | 99,140 | 88,929 | 98.41 | 94.73 | 78,807 | 66 | 1.000000 | 0.996147 |
| FRC_G15R_72h_3 | 95,655 | 83,964 | 98.38 | 94.73 | 72,545 | 77 | 1.000000 | 0.994857 |
| FRC_G30R_0h_1 | 52,954 | 38,096 | 98.36 | 94.82 | 21,495 | 12 | 1.000000 | 0.999527 |
| FRC_G30R_0h_2 | 68,611 | 48,754 | 98.21 | 94.11 | 35,214 | 14 | 1.000000 | 0.999724 |
| FRC_G30R_0h_3 | 65,906 | 44,065 | 98.23 | 94.29 | 19,810 | 7 | 1.000000 | 0.999862 |
| FRC_G30R_24h_1 | 57,356 | 42,675 | 98.10 | 94.14 | 27,872 | 7 | 1.000000 | 1.000000 |
| FRC_G30R_24h_2 | 55,172 | 41,820 | 98.20 | 94.39 | 28,762 | 9 | 1.000000 | 1.000000 |
| FRC_G30R_24h_3 | 108,692 | 77,443 | 98.18 | 94.37 | 59,322 | 22 | 1.000000 | 0.998688 |
| FRC_G30R_48h_1 | 103,632 | 78,402 | 98.53 | 95.13 | 72,927 | 34 | 1.000000 | 0.998619 |
| FRC_G30R_48h_2 | 104,040 | 77,288 | 98.53 | 94.86 | 71,058 | 28 | 0.999986 | 0.998923 |
| FRC_G30R_48h_3 | 106,639 | 80,778 | 98.54 | 94.80 | 73,899 | 31 | 0.999986 | 0.999429 |
| FRC_G30R_72h_1 | 108,450 | 94,618 | 98.45 | 94.80 | 84,211 | 51 | 1.000000 | 0.997143 |
| FRC_G30R_72h_2 | 108,196 | 93,724 | 98.44 | 94.75 | 85,323 | 55 | 1.000000 | 0.997714 |
| FRC_G30R_72h_3 | 115,567 | 98,564 | 98.46 | 94.88 | 88,704 | 53 | 1.000000 | 0.997455 |
| FRC_G45R_0h_1 | 54,746 | 35,370 | 98.26 | 94.48 | 23,643 | 11 | 1.000000 | 0.999751 |
| FRC_G45R_0h_2 | 80,450 | 51,961 | 98.34 | 94.81 | 38,683 | 18 | 1.000000 | 0.999714 |
| FRC_G45R_0h_3 | 54,273 | 35,252 | 98.33 | 94.85 | 22,026 | 10 | 1.000000 | 0.999882 |
| FRC_G45R_24h_1 | 67,300 | 51,643 | 98.06 | 94.00 | 37,050 | 7 | 1.000000 | 1.000000 |
| FRC_G45R_24h_2 | 66,319 | 49,848 | 98.23 | 94.47 | 35,292 | 8 | 1.000000 | 0.999845 |
| FRC_G45R_24h_3 | 60,754 | 43,181 | 98.20 | 94.34 | 24,764 | 7 | 1.000000 | 1.000000 |
| FRC_G45R_48h_1 | 90,608 | 63,447 | 98.41 | 94.68 | 49,908 | 14 | 1.000000 | 0.999470 |
| FRC_G45R_48h_2 | 100,871 | 70,174 | 98.35 | 94.43 | 54,851 | 22 | 0.999982 | 0.999503 |
| FRC_G45R_48h_3 | 105,418 | 72,454 | 98.52 | 95.23 | 56,797 | 18 | 1.000000 | 1.000000 |
| FRC_G45R_72h_1 | 99,517 | 82,059 | 98.53 | 95.08 | 76,534 | 31 | 1.000000 | 0.998857 |
| FRC_G45R_72h_2 | 101,240 | 86,702 | 98.47 | 94.93 | 82,582 | 30 | 1.000000 | 0.998313 |
| FRC_G45R_72h_3 | 110,425 | 90,952 | 98.51 | 95.00 | 85,172 | 32 | 1.000000 | 0.999143 |
| FRC_G60R_0h_1 | 53,199 | 38,501 | 98.20 | 94.45 | 23,281 | 10 | 1.000000 | 0.999586 |
| FRC_G60R_0h_2 | 56,622 | 40,769 | 98.28 | 94.64 | 33,971 | 12 | 1.000000 | 0.999467 |
| FRC_G60R_0h_3 | 67,080 | 44,954 | 98.32 | 94.76 | 35,218 | 13 | 1.000000 | 1.000000 |
| FRC_G60R_24h_1 | 111,449 | 85,875 | 98.31 | 94.59 | 69,877 | 20 | 1.000000 | 0.998864 |
| FRC_G60R_24h_2 | 120,542 | 85,450 | 98.26 | 94.47 | 65,375 | 15 | 1.000000 | 1.000000 |
| FRC_G60R_24h_3 | 124,143 | 87,945 | 98.22 | 94.37 | 63,814 | 16 | 1.000000 | 0.998895 |
| FRC_G60R_48h_1 | 104,589 | 84,920 | 98.47 | 94.71 | 76,150 | 33 | 1.000000 | 0.997714 |
| FRC_G60R_48h_2 | 97,315 | 82,673 | 98.50 | 95.04 | 76,032 | 38 | 1.000000 | 0.998044 |
| FRC_G60R_48h_3 | 109,196 | 92,140 | 98.45 | 94.55 | 82,359 | 27 | 1.000000 | 0.998571 |
| FRC_G60R_72h_1 | 120,472 | 88,797 | 98.29 | 94.57 | 68,031 | 41 | 1.000000 | 0.997143 |
| FRC_G60R_72h_2 | 110,005 | 82,282 | 98.40 | 94.87 | 63,763 | 38 | 1.000000 | 0.998088 |
| FRC_G60R_72h_3 | 99,112 | 72,031 | 98.51 | 95.20 | 62,229 | 30 | 1.000000 | 0.998343 |
| FRC_G75R_0h_1 | 57,772 | 34,043 | 98.74 | 95.84 | 30,755 | 19 | 1.000000 | 0.998964 |
| FRC_G75R_0h_2 | 55,378 | 32,910 | 98.60 | 95.17 | 29,910 | 22 | 0.999967 | 0.998379 |
| FRC_G75R_0h_3 | 69,582 | 41,339 | 98.66 | 95.34 | 37,563 | 17 | 0.999947 | 0.998895 |
| FRC_G75R_24h_1 | 100,126 | 73,046 | 98.34 | 94.69 | 57,586 | 15 | 1.000000 | 0.999337 |
| FRC_G75R_24h_2 | 70,538 | 52,043 | 98.32 | 94.42 | 36,837 | 18 | 1.000000 | 0.999420 |
| FRC_G75R_24h_3 | 105,413 | 82,270 | 98.35 | 94.68 | 65,549 | 20 | 1.000000 | 0.999448 |
| FRC_G75R_48h_1 | 114,319 | 82,964 | 98.47 | 95.04 | 70,468 | 23 | 1.000000 | 0.999734 |
| FRC_G75R_48h_2 | 108,807 | 79,478 | 98.37 | 94.72 | 66,264 | 18 | 1.000000 | 0.999429 |
| FRC_G75R_48h_3 | 108,661 | 78,704 | 98.44 | 95.03 | 64,140 | 20 | 1.000000 | 0.998875 |
| FRC_G75R_72h_1 | 105,206 | 78,501 | 98.32 | 94.64 | 61,667 | 22 | 1.000000 | 0.999429 |
| FRC_G75R_72h_2 | 115,151 | 85,263 | 98.45 | 94.89 | 64,175 | 23 | 1.000000 | 0.999438 |
| FRC_G75R_72h_3 | 123,038 | 92,987 | 98.49 | 95.06 | 78,545 | 28 | 1.000000 | 0.998857 |

# **11.Supplementary Figure S8.**

**Caption:**

(a) Complete batch-matched Spearman rank correlation (*ρ*) heatmap across 16 eligible bacterial genera and 12 key LC–MS metabolites (*n*=21 paired batches).
(b) Representative linear regression trajectories (g1–g4) between centered log-ratio (CLR) bacterial genus abundances and log_2_ -transformed metabolite intensities (*Lactococcus* A vs. lactate [g1], *Weissella* A vs. lactate [g2], *Klebsiella* vs. theobromine [g3], and *Pseudescherichia* vs. sucrose [g4]; shaded bands indicate 95% confidence intervals, with unadjusted *ρ*, *q* values, and treatment-adjusted *ρ* annotated).
(c) Network hub strength quantified by summed absolute Spearman correlation coefficients (∑∣*ρ*∣) for top LC–MS metabolite and bacterial genus nodes.
(d) Chemical-class edge balance showing the counts of retained negative (blue) and positive (red) associative edges across six major metabolite classes.

# **12.Supplementary Table S4.**

**Techno-economic analysis (TEA) parameters, capital/operating expenditure breakdowns, and 10-year cash flow projections for the simulated 500 L continuous-loop sonobioreactor**

**S4A. Capital expenditure (CapEx) and straight-line depreciation schedule for the 500 L external-loop multiprobe sonobioreactor system**

Engineering basis: Continuous external-loop reactor (working volume = 400 L, total vessel volume = 500 L) operating 300 days per year (100 batches yr⁻¹, 72 h cycle, annual throughput = 50 metric tons roasted-bean equivalent). Straight-line depreciation is calculated over a 10-year project horizon, while high-wear Ti-6Al-4V ultrasonic horns are depreciated over 2.5 years.

| **Equipment / Capital Component** | **Engineering Specification & Design Basis** | **Installed Cost (USD)** | **CapEx Share (%)** | **Service Life (Years)** | **Salvage Value (%)** | **Annual Depreciation (USD yr⁻¹)** |
| --- | --- | --- | --- | --- | --- | --- |
| Jacketed 316L stainless-steel main bioreactor vessel (500 L) | V_work = 400 L, sanitary mirror-polished, 0.3 MPa | 38,000 | 20.5% | 10 | 10% | 3,420 |
| Multi-probe ultrasonic flow-cell modules (20 kHz, 6 × 2.5 kW) | In-line series flow-through chambers, radial r = 30 cm | 45,000 | 24.3% | 10 | 10% | 4,050 |
| Ti-6Al-4V titanium alloy stepped acoustic horns (Replaceable) | High-fatigue resistance titanium alloy radiating probes | 12,000 | 6.5% | 2.5 (Consumable) | 0% | 4,800 |
| Digital frequency-tracking ultrasonic generators (50% duty cycle) | Automatic impedance matching, pulsed mode (5 s on / 5 s off) | 32,000 | 17.3% | 10 | 10% | 2,880 |
| Sanitary progressive-cavity recirculation pump & loop piping | Low-shear sanitary lobe pump (flow rate = 2.5 m³ h⁻¹) | 14,500 | 7.8% | 10 | 10% | 1,305 |
| External chiller & closed-loop jacketed cooling system (3.5 L kg⁻¹) | Isothermal control at 30 ± 0.5 °C, 15 kW cooling capacity | 16,500 | 8.9% | 10 | 10% | 1,485 |
| PLC automated control, multi-point thermocouple & pH/DO sensors | Real-time temperature feedback & acoustic power interlock | 15,000 | 8.1% | 10 | 5% | 1,425 |
| Equipment installation, sanitary valving, and commissioning | Piping integration, electrical wiring, and safety shielding | 12,000 | 6.5% | 10 | 0% | 1,200 |
| **Total Capital Expenditure (Total CapEx) & Annualized Depreciation** | **10-year project horizon (50 metric tons/year capacity)** | **185,000** | **100.0%** | **—** | **—** | **20,565** |

**S4B. Granular breakdown of direct operating expenditures (Direct OpEx = 1,050 USD ton⁻¹) and sensitivity parameter boundaries (RF60 protocol)**

Direct OpEx is normalized per metric ton (USD ton⁻¹) and per kilogram (USD kg⁻¹) of roasted coffee (green-bean equivalent). Pulsed sonication (0.5 W mL⁻¹, 50% duty cycle: 5 s on / 5 s off) requires 0.20 kWh kg⁻¹ (0.020 USD kg⁻¹, accounting for 1.90% of total direct OpEx). Sensitivity ranges reflect regional industrial electricity tariffs (0.06–0.15 USD kWh⁻¹) and seasonal fluctuations in Rosa 'Crimson Glory' petal procurement (±20%).

| **Operating Cost Component (OpEx)** | **Specific Consumption (per ton coffee)** | **Unit Price / Tariff (Baseline)** | **Cost per Ton (USD ton⁻¹)** | **Cost per kg (USD kg⁻¹)** | **OpEx Share (%)** | **Sensitivity Analysis Range & Impact (Fig. 11h)** |
| --- | --- | --- | --- | --- | --- | --- |
| Rosa 'Crimson Glory' fresh petals procurement (60% w/w loading) | 600 kg petals ton⁻¹ coffee | 1.10 USD kg⁻¹ petals | 660.0 | 0.660 | 62.86% | ±20% (0.88–1.32 USD kg⁻¹) -> 528–792 USD ton⁻¹ |
| Commercial starter culture (Saccharomyces cerevisiae, 10⁷ CFU g⁻¹) | 2.5 kg starter ton⁻¹ coffee | 36.00 USD kg⁻¹ starter | 90.0 | 0.090 | 8.57% | ±10% (32.4–39.6 USD kg⁻¹) -> 81–99 USD ton⁻¹ |
| Ultrasonic generator direct electricity (Pulsed 50% duty, 0.20 kWh kg⁻¹) | 200 kWh ton⁻¹ (0.20 kWh kg⁻¹) | 0.10 USD kWh⁻¹ (Industrial base) | 20.0 | 0.020 | 1.90% | 0.06–0.15 USD kWh⁻¹ -> 12.0–30.0 USD ton⁻¹ |
| Jacketed cooling water circulation & recirculation pump electricity | 150 kWh ton⁻¹ (0.15 kWh kg⁻¹) | 0.10 USD kWh⁻¹ (Industrial base) | 15.0 | 0.015 | 1.43% | 0.06–0.15 USD kWh⁻¹ -> 9.0–22.5 USD ton⁻¹ |
| Process water, sanitary CIP cleaning & wastewater treatment (3.5 L kg⁻¹) | 3.5 m³ water ton⁻¹ coffee | 10.00 USD m⁻³ (Water + CIP) | 35.0 | 0.035 | 3.33% | ±15% (8.5–11.5 USD m⁻³) -> 29.8–40.3 USD ton⁻¹ |
| Direct operating labor & quality assurance monitoring | 12.0 operator-hours ton⁻¹ | 15.00 USD h⁻¹ labor rate | 180.0 | 0.180 | 17.14% | ±15% (12.8–17.3 USD h⁻¹) -> 153–207 USD ton⁻¹ |
| Routine maintenance, horn tip replacement & consumables | Scheduled horn & seal upkeep | Fixed per-ton allocation | 50.0 | 0.050 | 4.76% | ±20% horn wear rate -> 40.0–60.0 USD ton⁻¹ |
| **Total Direct Operating Expenditure (Direct OpEx, RF60 Process)** | **Per metric ton roasted-bean equiv.** | **Baseline scenario** | **1,050.0** | **1.050** | **100.00%** | **Combined range: 898.0–1,215.5 USD ton⁻¹ (Payback: 0.88–1.18 yr)** |

**S4C. Ten-year discounted cash flow (DCF) analysis and investment profitability metrics (in thousand USD, kUSD)**

Financial modeling assumptions: Annual capacity = 50 tons yr⁻¹ (80% utilization in Year 1; 100% in Years 2–10). Working capital (WC = 25.0 kUSD in Year 1) and terminal equipment salvage value (16.3 kUSD) are recovered in Year 10 (+41.3 kUSD). While retail valuation of Grand Reserve specialty coffee (SCA score = 91.8) reaches 52.0 USD kg⁻¹ (vs. 9.8 USD kg⁻¹ baseline, Fig. 11g), the plant-gate cash flow model applies a conservative wholesale B2B processing price of 12.80 USD kg⁻¹ (vs. 6.50 USD kg⁻¹ green-bean procurement cost + 1.05 USD kg⁻¹ direct co-fermentation OpEx) to avoid retail mark-up inflation. At an 8% discount rate and 15% corporate tax rate, the facility achieves a Net Present Value (NPV) of 1,287.8 kUSD and a dynamic capital payback period of 1.02 years (ranging from 0.88 to 1.18 years across sensitivity scenarios in S4B).

| **Project Year** | **Capacity Utilization** | **CapEx & Working Capital (kUSD)** | **Gross Process Revenue (kUSD)** | **Raw Bean + Direct OpEx (kUSD)** | **Depreciation (kUSD)** | **Corporate Tax (15%, kUSD)** | **Net Cash Flow NCF (kUSD)** | **Discounted CF DCF (8%, kUSD)** | **Cumulative DCF & Payback (kUSD)** |
| --- | --- | --- | --- | --- | --- | --- | --- | --- | --- |
| Year 0 | Construction | -185.0 | 0.0 | 0.0 | 0.0 | 0.0 | -185.0 | -185.0 | -185.0 |
| Year 1 | 80% | -25.0 (WC) | 512.0 | -302.0 | -20.6 | -28.4 | 156.6 | 145.0 | -40.0 |
| Year 2 | 100% | 0.0 | 640.0 | -377.5 | -20.6 | -36.3 | 226.2 | 193.9 | +153.9 |
| Year 3 | 100% | 0.0 | 640.0 | -377.5 | -20.6 | -36.3 | 226.2 | 179.6 | +333.5 |
| Year 4 | 100% | 0.0 | 640.0 | -377.5 | -20.6 | -36.3 | 226.2 | 166.3 | +499.8 |
| Year 5 | 100% | 0.0 | 640.0 | -377.5 | -20.6 | -36.3 | 226.2 | 154.0 | +653.8 |
| Year 6 | 100% | 0.0 | 640.0 | -377.5 | -20.6 | -36.3 | 226.2 | 142.6 | +796.4 |
| Year 7 | 100% | 0.0 | 640.0 | -377.5 | -20.6 | -36.3 | 226.2 | 132.0 | +928.4 |
| Year 8 | 100% | 0.0 | 640.0 | -377.5 | -20.6 | -36.3 | 226.2 | 122.2 | +1,050.6 |
| Year 9 | 100% | 0.0 | 640.0 | -377.5 | -20.6 | -36.3 | 226.2 | 113.2 | +1,163.8 |
| Year 10 | 100% | +41.3 (Rec) | 640.0 | -377.5 | -20.6 | -36.3 | 267.5 | 124.0 | +1,287.8 |
| **Cumulative / KPI** | **490 tons total** | **-168.7 Net CapEx** | **6,272.0** | **-3,699.5** | **-205.7** | **-355.1** | **2,049.7** | **NPV = 1,287.8 kUSD** | **Payback = 1.02 yr (0.88–1.18 yr)** |
